# Purifying Selection and Interspecific Differentiation at the *Myf5* Locus Inform Hybrid Identification and Genetic Management of Thai Clariid Resources

**DOI:** 10.3390/genes17080920

**Published:** 2026-08-04

**Authors:** Phonemany Thammachak, Ton Huu Duc Nguyen, Rinrapat Nitipatpornpanya, Anh Huynh Luu, Edem Uduak Linus, Thitipong Panthum, Kednapat Sriphairoj, Sittichai Hatachote, Satid Chatchaiphan, Chaiwut Grudpan, Jarungjit Grudpan, Suphada Kiriratnikom, Jiraboon Prasanpan, Orathai Sawatdichaikul, Worapong Singchat, Kornsorn Srikulnath

**Affiliations:** 1 Animal Genomics and Bioresource Research Unit (AGB Research Unit), Faculty of Science, Kasetsart University, Bangkok 10900, Thailand; phonemamy.t@ku.th (P.T.); nguyenhuuduc.t@ku.th (T.H.D.N.); rinrapat.ni@ku.th (R.N.); anhhuynh.l@ku.th (A.H.L.); edemlinus@unical.edu.ng (E.U.L.); thitipong.pant@ku.ac.th (T.P.); 2 Department of Genetics, Faculty of Science, Kasetsart University, Chatuchak, Bangkok 10900, Thailand; 3 Interdisciplinary Graduate Program in Bioscience, Faculty of Science, Kasetsart University, 50 Ngamwongwan, Chatuchak, Bangkok 10900, Thailand; 4 Faculty of Biology Education, School of Education, Can Tho University, 3/2 Street, Ninh Kieu Ward, Can Tho 900000, Vietnam; 5 Plant Genetics and Biotechnology Unit, Department of Genetics and Biotechnology, University of Calabar, Calabar PMB 1115, Nigeria; 6 Faculty of Natural Resources and Agro-Industry, Kasetsart University, Chalermphrakiat Sakon Nakhon Province Campus, Sakon Nakhon 47000, Thailand; kednapat.sr@ku.th (K.S.); sittichai.h@ku.th (S.H.); 7 Department of Aquaculture, Faculty of Fisheries, Kasetsart University, Bangkok 10900, Thailand; satid.ch@ku.th; 8 Department of Fisheries, Faculty of Agriculture, Ubon Ratchathani University, Ubon Ratchathani 34190, Thailand; chaiwut.k@ubu.ac.th (C.G.); jarungjit.g@ubu.ac.th (J.G.); 9 Department of Fisheries Science and Aquatic Resources, Faculty of Science and Digital Innovation, Thaksin University, Phatthalung 93210, Thailand; suphada.k@tsu.ac.th; 10 Kalasin Fish Hatchery (Betagro Public Company Limited), Kalasin 46000, Thailand; jiraboonp@betagro.com; 11 Department of Nutrition and Health, Institute of Food Research and Product Development, Kasetsart University, Bangkok 10900, Thailand; orathai.saw@ku.th; 12 Biodiversity Center, Kasetsart University (BDCKU), Bangkok 10900, Thailand

**Keywords:** genetic introgression, marker-assisted selection, myogenesis, hatchery stocks, inbreeding depression

## Abstract

**Background/Objectives**: Clariid catfishes are a cornerstone of Southeast Asian aquaculture, where intensive breeding and interspecific hybridization have significantly decreased genetic diversity. To address the limited understanding of growth-governing molecular determinants, we characterized the genomic architecture and evolutionary dynamics of the myogenic factor 5 (*Myf5*) gene, a master regulator of myogenesis, in 511 individuals from 17 catfish populations in Thailand. **Methods**: A total of 511 individuals representing 17 catfish populations were analyzed. A 334-bp fragment of exon 1 of the *Myf5* gene was sequenced using Illumina short-read technology. Genetic diversity indices were calculated to evaluate sequence variation, population structure, and selective constraints acting on the locus. **Results**: Sequence analysis of a 334-bp segment of exon 1 using Illumina short-read technology identified 17 haplotypes and 92 variable sites, which revealed moderate allelic richness despite low overall nucleotide diversity (*π* ≈ 0.008). Evolutionary analyses indicated that the *Myf5* locus is governed by stringent purifying selection (*ω* = 0.063), wherein site-specific constraints preserve the stability of the α-helix folding architecture, which is essential for myogenic regulation. Population genetic analyses revealed substantial differentiation among taxa (56% among-population variation), while Bayesian clustering successfully resolved a hierarchical structure (*K* = 2) that distinguished *Clarias gariepinus*, *Clarias macrocephalus*, and *Clarias batrachus*. These findings demonstrate that species-specific *Myf5* haplotypes serve as diagnostic markers for identifying parental species and assisting in the identification of F_1_ hybrid stocks. **Conclusions**: Overall, this study provides the molecular markers for species identification, broodstock authentication, hybrid detection, and sustainable genetic management of Thai Clariid resources, which are critical for balancing production efficiency with the conservation of native biodiversity in Southeast Asia.

## 1. Introduction

Clariid catfishes, most notably African catfish (*Clarias gariepinus*), an introduced species in Thailand, together with the native bighead catfish (*Clarias macrocephalus*) and walking catfish (*Clarias batrachus*), constitute the primary aquaculture taxa across Southeast Asia and play a vital role in food security and economic infrastructure of Thailand. These species are essential sources of high-quality protein and polyunsaturated fatty acids, with a national production of approximately 100,000 tons and a market value exceeding US$150 million in 2020 [1]. The long-term sustainability of this industry, which is increasingly focused on preserving genetic resources, depends on its ability to monitor and manage the genetic health of both wild and hatchery-reared stocks [2]. Significant interspecific variation in growth performance has been consistently documented, with *C. gariepinus* demonstrating superior growth rates to *C. macrocephalus* and *C. batrachus* [3,4,5,6]. To exploit these advantageous traits, a hybridization program between female *C. macrocephalus* and male *C. gariepinus* was initiated in 1987 to integrate rapid growth and enhanced disease resistance. By 2004, these hybrids reportedly accounted for >90% of the total catfish production in Thailand [4,5,7]. Although the recent availability of high-quality chromosome-level genome assemblies has provided a robust genomic framework for this genus [8,9,10], the specific molecular drivers of these growth disparities remain only partially understood. Although these selective breeding strategies have substantially improved productivity, they have concurrently contributed to a decline in genetic diversity. The repeated use of limited broodstock lines of limited size has increased inbreeding/outbreeding risk, leading to reduced survival and reproductive capacity [4]. Moreover, the extensive application of interspecific hybridization poses a threat to native species, particularly *C. macrocephalus* and *C. batrachus*, through genetic introgression, which may lead to population displacement [6,11]. Although genes associated with muscle growth are beginning to be identified owing to advances in comparative transcriptome analyses [12], a critical gap remains in our understanding of specific master regulatory genes, such as myogenic factor 5 (*Myf5*), which serve as key regulators of myogenic development and may contribute to species- and population-specific variation in growth traits.

The myogenic regulatory factor (MRF) family, including *Myf5*, *MyoD*, myogenin, and *MRF4*, is central to the regulation of skeletal muscle growth. These proteins function as basic helix–loop–helix (bHLH) transcription factors that dimerize with E-proteins and bind to E-box sequences for activating muscle-specific gene expression [13]. Among MRFs, *Myf5* is typically expressed at the earliest stage of myogenesis during somitogenesis, where it plays a pivotal role in the determination and proliferation of myogenic precursor cells [14]. DNA sequence analyses indicate that *Myf5* is highly conserved among teleosts and encodes a protein with a characteristic bHLH domain responsible for DNA binding and transcriptional regulation [15,16,17,18,19,20,21]. In fishes, skeletal muscle growth occurs through both hyperplasia and hypertrophy, which are tightly coordinated by MRFs, including *Myf5* [13,22]. Beyond its developmental role, *Myf5* expression is affected by nutritional factors and environmental stressors, such as dietary carbohydrate levels, amino acid supplementation, and ammonia stress, highlighting its significance in physiological adaptation and muscle growth regulation [23,24,25,26]. Considering these essential functions, *Myf5* is expected to be under stringent evolutionary constraints, which are typically quantified through the *dN*/*dS* ratio (*ω*). However, a significant knowledge gap persists regarding the molecular evolution and allelic diversity of *Myf5* among *Clarias* species and populations, where the interplay between intensive selection in aquaculture and genetic drift in wild habitats may shape unique genomic signatures. *Myf5* was selected as the focal candidate locus for this study because it combines strong functional relevance with potentially informative interspecific and population-level sequence variation. As an early regulator of myogenic commitment, *Myf5* is expected to retain conserved coding regions under functional constraint, while polymorphic sites outside the most constrained residues may still preserve lineage-specific genetic signals. Such candidate functional loci are not substitutes for genome-wide neutral markers, but they can provide complementary information for species discrimination, assessment of allelic differentiation, and evaluation of introgression in closely related taxa. Resolving the complex population architecture of Thai catfish requires an integrated approach that extends beyond single-gene studies. The widespread distribution of hybrids and potential for overlapping genetic variations at microgeographic scales often obscure clear species boundaries. In addition to the focal analysis of *Myf5*, we evaluated population subdivision using a three-locus nuclear dataset comprising *Myf5*, myostatin b (*Mstnb*), and growth hormone 1 (*GH1*) gene loci. These loci were included as complementary candidate-gene markers rather than as substitutes for genome-wide neutral SNPs. Importantly, multilocus analyses were restricted to individuals for which genotypes at all three loci could be matched using consistent specimen identifiers. This multi-locus framework is essential for the sustainable management of both wild and cultured genetic resources, which currently face threats from hybridization and reduced genetic diversity.

Despite the recognized importance of *Myf5* in myogenesis, a significant knowledge gap exists regarding its high-resolution allelic diversity and the molecular signatures of adaptation across wild and cultured Thai *Clarias* populations. This is particularly pronounced in the context of interspecific hybridization, where a lack of comparative genomic data on *Myf5* limits the ability to delineate species-specific lineages and resolve complex population structures. Therefore, in this study, we addressed the question of how molecular variation within the *Myf5* locus reflects evolutionary selective pressure and contributes to the hierarchical genetic architecture of clariid catfishes. The exon 1 fragment was selected following comparative screening of available genomic sequences, which indicated that this region contains a relatively high density of informative polymorphic sites while remaining within a functionally relevant coding region. This combination made the fragment suitable for testing whether locus-specific variation could provide complementary resolution of species identity and population differentiation. We hypothesized that although the *Myf5* gene is subjected to stringent purifying selection to preserve its essential myogenic regulatory function, it harbors sufficient population-specific polymorphisms to differentiate between native species and their hybrids. Our primary purpose in this study was to characterize the genomic architecture of the *Myf5* exon 1 region in 511 individuals, allowing us to evaluate the distribution of genetic diversity within and among species. In this experimental framework, the independent variables were 17 distinct sampling locations, and 11 cultured and six wild populations of four population types (*C. gariepinus*, *C. macrocephalus*, *C. batrachus*, and their hybrids). The dependent variables included several key genetic indices such as allelic and haplotype variations, population differentiation parameters, and evolutionary signatures. By integrating Bayesian clustering with phylogenetic reconstruction, we aimed to determine whether *Myf5* variation could support species discrimination, assessment of population structure, and monitoring of parental contributions in hybrid stocks. The findings are expected to provide a genetic baseline for broodstock identification, hybrid monitoring, and conservation-oriented management of native catfish populations in Thailand. Accordingly, the dataset generated in this study was used to address three related objectives: to assess whether variation at the *Myf5* locus can distinguish closely related *Clarias* species and hybrid individuals, to compare genetic diversity and population differentiation among wild and hatchery-derived populations, and to evaluate how integration of *Myf5* data with *Mstnb* and *GH1* data improves the resolution of population structure. Together, these analyses link locus-specific variation with the broader challenges of introgression monitoring, broodstock management, and conservation of native catfish genetic resources.

## 2. Materials and Methods

### 2.1. Specimen Acquisition and Genomic DNA Isolation

**Figure 1:**
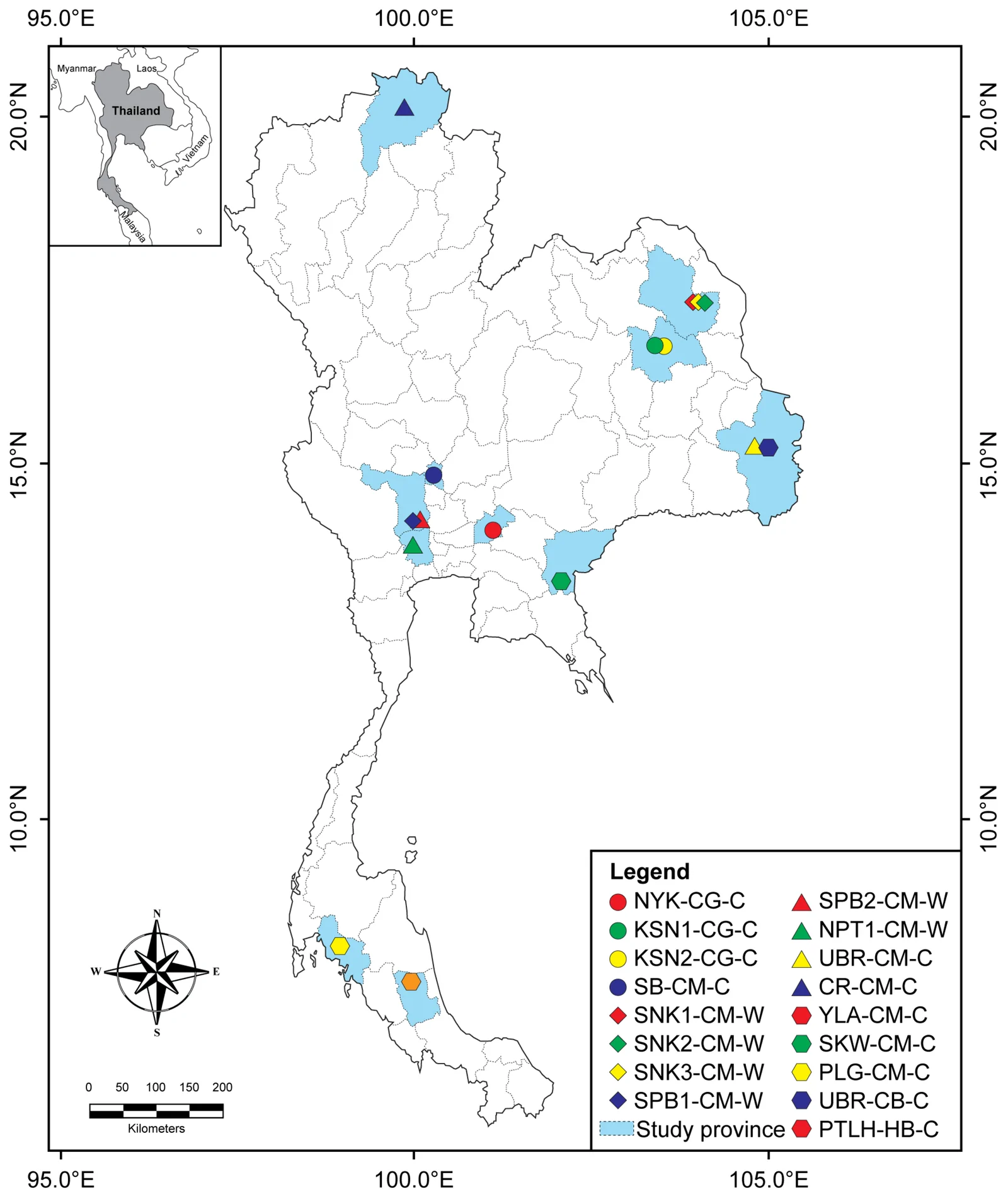
Distribution of sampling sites in Thailand. Province abbreviations are: NYK, Nakhon Nayok; KSN, Kalasin; SB, Sing Buri; SNK, Sakon Nakhon; SPB, Suphan Buri; NPT, Nakhon Pathom; UBR, Ubon Ratchathani; CR, Chiang Rai; YLA, Yala; SKW, Sa Kaeo; PLG, Phang Nga; and PTLH, Phatthalung. Species abbreviations are: CG, *Clarias gariepinus*; CM, *C. macrocephalus*; CB, *C. batrachus*; and HB, hybrid catfish. The final letter denotes the sampling source, where C indicates cultured populations and W indicates wild populations. Numerals distinguish different sampling locations within the same province (e.g., KSN1 and KSN2).

In total, 511 catfish specimens, comprising both cultured and wild individuals, were collected from 17 distinct sampling sites across Thailand (Figure 1; Table S1), including 11 cultured populations and 6 wild populations. The study dataset included three populations of African catfish, twelve populations of bighead catfish, one population of walking catfish, and one hybrid population, sourced from various provinces nationwide (Figure 1; Table S1). The sampling effort among taxa was uneven because of differences in specimen availability. Specifically, *C. macrocephalus* and *C. gariepinus* were represented by 12 and three populations, respectively, whereas *C. batrachus* and the hybrid group were each represented by a single population. Therefore, estimates for the latter groups should be interpreted as population-specific observations rather than species-wide estimates. For each specimen, a small fin-tissue fragment (approximately 0.3 × 0.3 cm) was excised from the caudal fin and preserved in 95% ethanol in 1.5 mL microcentrifuge tubes, which were subsequently stored at 4 °C until further analysis. All sampling procedures were performed with explicit authorization from farm owners or relevant local authorities, and all individuals were promptly released back into their culture systems or natural habitats following tissue collection. These experimental protocols, conducted in strict accordance with institutional regulations and the Animal Research Reporting of In Vivo Experiments (ARRIVE) guidelines (https://arriveguidelines.org), were approved by the Animal Experiment Committee of Kasetsart University (Approval Nos. ACKU65-SCI-003, ACKU66-SCI-006, and ACCU66-SCI-014). Genomic DNA was isolated using a standard salting-out protocol described by Supikamolseni et al. [27]. To ensure the integrity of downstream analyses, the quality and concentration of extracted DNA were evaluated by 1% agarose gel electrophoresis and spectrophotometric quantification using a NanoDrop 2000 (Thermo Fisher Scientific, Wilmington, DE, USA).

### 2.2. Selection of Target Loci and Candidate Gene Characterization

The *Myf5* gene was identified by aligning in-house whole-genome sequencing (WGS) data with homologous sequences from various teleosts, which were retrieved from public data repositories (Table S3). The initial comparative analysis delineated a genomic architecture comprising three exons and two introns. Exon 1 exhibited the highest nucleotide variability. As this specific region contained a dense concentration of informative polymorphic sites that provided the resolution required for robust haplotypic diversity and phylogenetic analyses, it was designated as the primary target locus for this study. Sequence evaluations were performed to ensure that the selected exons, representing the most divergent segments of the gene, were optimal for distinguishing the studied catfish populations.

### 2.3. PCR Amplification and Illumina-TM Short-Read Sequencing

A partial region of the *Myf5* gene exon 1 was amplified by polymerase chain reaction (PCR). The amplification employed the primer pair Myf5_Catfish_CGA_F1 (5′-TCATCTCTCCTCTCCTCCAC-3′) and Myf5_Catfish_CGA_R1 (5′-TGTAGTTGATGGCGTTCCTC-3′), which we designed based on the chromosome 12 sequence of African catfish (*C. gariepinus*; accession number NC071111.1). An 8-bp sample-specific barcode sequence was incorporated at the 5′ end of the forward primer (Macrogen Inc., Seoul, Republic of Korea). PCR assays were conducted in a total reaction volume of 15 μL, comprising 50 ng of genomic DNA, 1× standard Apsalagen reaction buffer, 1.5 mM MgCl_2_, 0.2 mM of each dNTP, 0.5 μM of each primer, and 0.5 U Taq DNA polymerase (Apsalagen Co., Ltd., Bangkok, Thailand).

PCR amplification was carried out under the following thermal cycling conditions: an initial denaturation at 95 °C for 10 min; followed by 40 cycles comprising denaturation at 95 °C for 30 s, annealing at 60 °C for 30 s, and extension at 72 °C for 30 s; and a final extension step at 72 °C for 5 min. The amplified products were then examined by electrophoresis on a 1.5% agarose gel. To minimize the risk of false haplotype detection, each sample was independently amplified in triplicate. In total, 92 samples per pool set were amplified using distinct barcode-labelled primers, followed by grouping into nine separate pools and sequencing using a paired-end short-read approach on the Illumina NovaSeq™ 6000 platform (Novogene Co., Ltd., Singapore).

### 2.4. Raw Data Filtering and Bioinformatics Quality Assessment

The quality of the 334-bp paired-end reads was evaluated using FastQC version 0.11.9 [28], which allowed for the implementation of a stringent quality score threshold (q > 20). Following this initial assessment, individual amplicon sequences of *Myf5* haplotypes were processed using the AmpliSAS pipeline [29] (https://onlinelibrary.wiley.com/doi/full/10.1111/1755-0998.12453, accessed on 18 March 2026), which facilitated clustering, filtering, and assignment of haplotypes to each specimen based on read counts. To eliminate potential sequencing artifacts and exclude samples with insufficient coverage, a minimum amplicon depth of 100 reads was required. In accordance with the diploid nature of catfish species [30], the maximum number of haplotypes per individual was restricted to two, which ensured the accurate scoring of haplotypes. True alleles at a given position within the nucleotide sequence were identified by applying a degree-of-change criterion based on the sequencing depth, whereas all other parameters were maintained at default settings following the recommendations of Lighten et al. [31]. Genotypes were classified as homozygous when the dominant haplotype frequency exceeded 80%, whereas individuals with two comparable haplotypes were designated as heterozygous. Each identified haplotype was further compared with *Myf5* sequences archived in the National Center for Biotechnology Information (NCBI) database using the BLASTn algorithm (https://blast.ncbi.nlm.nih.gov/Blast.cgi, accessed on 18 March 2026), with a minimum sequence identity of 90% required for species-level identification. Finally, all sequences were aligned and mapped to the reference *Myf5* gene of African catfish (accession number: XM_053509129), and subsequently translated into amino acid sequences using Geneious Prime version 2025.0.2 (https://www.geneious.com) to screen for the presence of premature stop codons.

### 2.5. Genetic Diversity Assessment and Statistical Analysis of Myf5 Allelic Variation

To establish a detailed profile of the nucleotide diversity of the *Myf5* gene, based on aligned 334-bp sequences of exon 1, we performed DNA polymorphism analyses using DnaSP version 6.12 [32]. For each population, we estimated several key diversity indices, including the number of segregating sites (*S*), number of alleles (*h*), allelic diversity (*Hd*), nucleotide diversities (*π*), and the average number of nucleotide differences (*k*). We also calculated additional genetic diversity parameters, including the allelic frequency, observed number of alleles (*N*_a_), number of effective alleles (*N*_e_), and both observed (*H*_o_) and expected (*H*_e_) heterozygosity using GenAlEx version 6.5 [33]. This software was also used to assess the fixation index (*F*) and deviations from Hardy–Weinberg equilibrium (HWE), which are essential for identifying potential selective pressures or non-random mating patterns within *Clarias* populations. To explore the genetic architecture and differentiation among these populations, we examined *F*-statistics (*F*_IS_ and *F*_ST_) and allelic richness (*AR*) using FSTAT version 2.9.3 [34] as described by Budi et al. [35]. We also performed an Analysis of Molecular Variance (AMOVA) using Arlequin version 3.5.2.2 [36] to determine the distribution of genetic variation across different hierarchical levels.

### 2.6. Phylogenetic Reconstruction and Evolutionary Divergence of Myf5 Lineages

Nucleotide sequences of the *Myf5* gene from flathead grey mullet (*Mugil cephalus*, XM047575926) and representative catfish species *Trichomycterus rosablanca* (XM063007419), *Silurus meridionalis* (XM046873826), *Pangasianodon hypophthalmus* (XM026909928), *Neoarius graeffei* (XM060903546), *Hemibagrus wyckioides* (XM058417451), *Ictalurus punctatus* (XM017493744), *Ictalurus furcatus* (XM053650830), *Tachysurus fulvidraco* (XM027155249), *Tachysurus vachellii* (XM060889611), *C. gariepinus* (XM053509129), and *C. batrachus* (KX258952) were obtained from the NCBI database using BLASTn (https://blast.ncbi.nlm.nih.gov/Blast.cgi, accessed on 18 March 2026), by applying thresholds of >70% sequence identity and >85% query coverage. The retrieved sequences were aligned separately using MAFFT v7.490 (https://mafft.cbrc.jp/alignment/software, accessed on 18 March 2026), using the alignment strategies typically employed for protein-coding genes. The optimal nucleotide substitution model was determined using ModelFinder (https://iqtree.github.io/ModelFinder, accessed on 18 March 2026) based on the Bayesian Information Criterion (BIC), which identified the GTR + G model as the best fit [37]. This model was subsequently applied to all downstream phylogenetic analyses.

To determine the phylogenetic relationships of the gene sequences, we used the Maximum Likelihood (ML) and Bayesian Inference (BI) methods. ML analyses were conducted using IQ-TREE v1.6.12 (https://iqtree.github.io, accessed on 18 March 2026), which used 10,000 ultrafast bootstrap replicates to ensure robust branch support estimates. BI analyses were performed using MrBayes v3.2.6 [38] (https://nbisweden.github.io/MrBayes, accessed on 20 March 2026), implemented within the Geneious Prime 2025.0.2 platform (https://www.geneious.com). This process involved running five Markov chain Monte Carlo (MCMC) chains for 2,000,000 generations, with sampling at every 5000 generations. To ensure parameter convergence, the initial 200,000 generations (10%) were discarded as burn-in. The resulting phylogeny was rooted using *M. cephalus*, a non-Siluriformes teleost species, which was designated a priori as the outgroup. For the final visualization and editing of phylogenetic trees, we utilized the Interactive Tree of Life (iTOL) v5 [39] (https://itol.embl.de/), which provides a comprehensive suite for graphical tree management. Pairwise genetic distances between sequences were calculated in MEGA 11 (https://www.megasoftware.net/) using the Kimura two-parameter (K2P) model.

### 2.7. Evaluation of Selective Pressures and Locus-Specific Evolutionary Constraints

To evaluate the selective pressures acting on the *Myf5* gene in Thai catfishes, we calculated the *dN*/*dS* ratio (*ω*), which represents the balance between non-synonymous (*d*_N_) and synonymous (*d*_S_) substitution rates per site. These values were estimated using the Nei–Gojobori method [40] along with a Jukes–Cantor correction, which was implemented in MEGA version 10.2.2 [41]. In this framework, an *ω* value near 1 indicates neutral evolution, whereas values significantly greater than 1 suggest positive selection and those below 1 indicate purifying selection. We also utilized the Branch-Site Unrestricted Statistical Test for Episodic Diversification (BUSTED), which was implemented on the Datamonkey server (https://www.datamonkey.org), to evaluate whether specific lineages, treated as foreground branches, exhibited elevated *ω* estimates. Statistical significance (*p* < 0.05) was established through a Likelihood Ratio Test (LRT) that compared an unconstrained model, where at least one site on at least one branch may experience diversifying selection (*ω* > 1), against a null model where such selection is strictly prohibited (*ω* ≤ 1) [42]. To further define the specific mode of selection, we performed several neutrality tests, including Tajima’s *D*, Fu and Li’s F*, and Fu and Li’s D*, using DnaSP version 6.12 [32]. The raw *p*-values that were derived from all neutrality tests across the diverse study populations were adjusted using the Benjamini–Hochberg False Discovery Rate (FDR) method, which accounts for the potential inflation of Type I errors due to multiple comparisons. This correction procedure was implemented in R version 4.5.2 [43] using the stats package (https://stat.ethz.ch/R-manual/R-devel/library/stats/html/00Index.html, accessed on 18 March 2026), where statistical significance was strictly determined using a *q*-value threshold of *q* < 0.05. Moreover, we investigated potential selective sweeps by plotting the *H*_e_ and *F*_IS_ values for the *Myf5* gene, which were derived from the genotyping data. Following the criteria described by Reddy et al. [44], the presence of high *F*_IS_ and low *H*_e_ suggests purifying or sweeping selection, whereas a combination of low *F*_IS_ and high *H*_e_ indicates neutral or balancing selection. Codon-level selection analyses were also conducted using the Datamonkey platform (https://www.datamonkey.org) with three complementary statistical models: (i) MEME (Mixed-Effects Model of Evolution), which detects episodic diversifying selection at individual codon sites [45]; (ii) FEL (Fixed-Effects Likelihood), which identifies sites under pervasive selection across the entire phylogeny [46]; and (iii) FUBAR (Fast Unconstrained Bayesian Approximation), which utilizes a Bayesian framework to infer codons evolving under persistent positive or purifying selection [47]. The statistical significance for these analyses was established using a threshold of *p* ≤ 0.01 for likelihood-based methods and a posterior probability (PP) of ≥ 0.9 for the Bayesian approach.

### 2.8. Multiple Sequence Alignment and Comparative Analysis of Myf5 Residues

Reference sequences of *Myf5* genes from different species (*M. cephalus* (XM047575926), *T. rosablanca* (XM063007419), *S. meridionalis* (XM046873826), *P. hypophthalmus* (XM026909928), *N. graeffei* (XM060903546), *H. wyckioides* (XM058417451), *I. punctatus* (XM017493744), *I. furcatus* (XM053650830), *T. fulvidraco* (XM027155249), *T. vachellii* (XM060889611), *C. gariepinus* (XM053509129), and *C. batrachus* (KX258952)) were retrieved from NCBI Protein database (https://blast.ncbi.nlm.nih.gov/Blast.cgi, accessed on 18 March 2026). The BLASTp analysis was conducted using thresholds of >60% sequence identity and >85% query coverage [48,49]. The amino acid residues of the *Myf5* haplotypes identified in this study and the reference sequences of other catfish species were aligned using ClustalW in Geneious Prime version 2025.0.2 (https://www.geneious.com).

After trimming the sequence alignment to 100 residues, we constructed a Bayesian phylogenetic tree using MrBayes v3.2.6, which employs MCMC analysis for 2,000,000 generations. This analysis, which utilized four chains and sampled every 1000 generations with a 25% burn-in, assessed branch support through posterior probabilities derived from two independent runs. To characterize the protein architecture, we predicted secondary structures using the PSIPRED Workbench (https://bioinf.cs.ucl.ac.uk/psipred, accessed on 18 March 2026). As a master myogenic regulatory factor, Myf5 directs early determination of muscle precursor cells. Therefore, amino acid substitutions within conserved regions, which may impair DNA-binding or interactions with other regulatory partners, can disrupt muscle cell proliferation and differentiation. Such modifications are expected to influence the efficiency of myogenic commitment, which ultimately modulates the basal growth regulatory pathway by either enhancing activity or altering cellular processing [50].

### 2.9. AlphaFold 3-Based Tertiary Structure Prediction and Functional Domain Alignment

The amino acid sequences that were derived from the partial exon 1 region of the *Myf5* gene were used to predict three-dimensional (3D) protein structures through the AlphaFold 3 prediction server (https://alphafoldserver.com/, accessed on 18 June 2026), which represents a cutting-edge platform for highly accurate molecular modeling [51]. The resulting structural models were visualized using BIOVIA Discovery Studio (Dassault Systèmes, San Diego, CA, USA) and subsequently evaluated for stereochemical quality using PROCHECK [52], where a Ramachandran plot analysis was conducted to examine the backbone integrity and conformational stability of the predicted residues. High-confidence models were then aligned and compared with a reference protein sequence obtained from the UniProt database (https://www.uniprot.org/) to identify conserved structural domains that may indicate functional similarities.

### 2.10. Assessment of Gene Pool Structure Through Integrated Polymorphisms of Myf5 with Mstnb and GH1

We characterized the genetic structure of the sampled populations using STRUCTURE v2.3.4 [53], following the multi-locus methodology optimized by Budi et al. [54]. To enhance genomic power of resolution, we integrated *Myf5* sequence data with data from the myostatin b (*Mstnb*) and growth hormone 1 (*GH1*) genes. The *Mstnb* and *GH1* sequences used in this study were not derived from independent public datasets, but rather from our previously published datasets generated from the same individuals analyzed here. These sequences have been deposited in public repositories under accession numbers PX961328–PX961335 (*Mstnb*) and PQ877069–PQ877074 (*GH1*) and were retrieved for integrated multilocus analyses [55,56]. The optimal number of genetic clusters (*K*) was determined using the Δ*K* method, based on changes in the log probability of the data, ln Pr(X|K), as implemented in the Δ*K* method, which was implemented via StructureSelector [57].

To complement Bayesian inference, we executed a Discriminant Analysis of Principal Components (DAPC) using the ADEGENET 2.0 package [58] in R v4.3.2 [43], which facilitated identification of genetic subdivisions without assuming Hardy–Weinberg equilibrium. Pairwise genetic distances (*p*-distance) for the 334-bp *Myf5* exon 1 sequences were calculated using MEGA 12 [59]. The resulting distance matrix served as the input for Principal Coordinate Analysis (PCoA) performed in R using the vegan package (https://cran.r-project.org/web/packages/vegan/index.html, accessed on 18 June 2026), where outputs were rendered using ggplot2 (https://cran.r-project.org/web/packages/ggplot2/index.html, accessed on 18 June 2026). In this visualization, individuals were color-coded according to species to illustrate the correlation between sequence divergence and population clustering [43].

## 3. Results

### 3.1. Analysis of Myf5 Locus Architecture and Interspecific Genetic Variation

Initial screening of in-house whole-genome sequencing data against public databases identified the *Myf5* gene as one of the most polymorphic loci based on SNP density (Table S3). Sequence characterization revealed a gene structural architecture comprising three exons and two introns. A comparative genomic sequence analysis using entries from public data repositories that included representative catfishes and distinct teleosts demonstrated an uneven distribution of nucleotide variation, with exons 1 and 2 emerging as a disproportionately polymorphic region. This 334-bp segment contained 92 variable sites (Table S4), providing a high density of informative markers essential for effective haplotypic differentiation. Moreover, the observed transition/transversion ratio (12/11) and presence of unique species-specific SNPs within this region confirmed its suitability for high-resolution genomic analysis.

### 3.2. Patterns of Molecular Variation and Genetic Differentiation Within and Among Clarias Lineages

We characterized a 334-bp partial fragment of the *Myf5* gene, which corresponds to the terminal region of exon 1, from 511 individuals representing 17 distinct populations (Table S2). Compared with the *C. gariepinus* reference sequence (XM_053509129), this analysis revealed 17 haplotypes (Table S3). Among these, *Clarias_Myf5TH1* emerged as the most prevalent variant, occurring in 13 of 17 sampled populations. By contrast, several populations harbored private haplotypes with highly restricted distributions. For instance, we identified haplotypes unique to specific wild and cultured populations, including SNK3-CM-W (*Clarias_Myf5TH4* and *Clarias_Myf5TH6*), UBR-CB-C (*Clarias_Myf5TH9* and *Clarias_Myf5TH10*), and SNK2-CM-W (*Clarias_Myf5TH14* and *Clarias_Myf5TH15*). Moreover, *Clarias_Myf5TH11*, *Clarias_Myf5TH16*, and *Clarias_Myf5TH17* were restricted to a single population each, specifically SBR-CM-C, KSN2-CG-C, and YLA-CM-C, respectively. At the species level, we identified several diagnostic haplotypes confined to specific taxa (Table S3). Two haplotypes (*Clarias_Myf5TH13* and *Clarias_Myf5TH17*) were found exclusively in *C. gariepinus*, whereas five haplotypes (*Clarias_Myf5TH4*, *Clarias_Myf5TH6*, *Clarias_Myf5TH11*, *Clarias_Myf5TH14*, and *Clarias_Myf5TH15*) were specific to *C. macrocephalus*. One haplotype (*Clarias_Myf5TH10*) was only detected in the single sampled *C. batrachus* population. Because *C. batrachus* and the hybrid group were each represented by only one population, these observations should not be interpreted as evidence of species-wide specificity and require validation using additional populations throughout the species’ ranges.

Genetic variation at the *Myf5* exon 1 locus, which displayed substantial heterogeneity across the 17 sampled populations, was characterized at the sequence level to evaluate population-specific diversity patterns (Table S3). The number of segregating sites (*S*) ranged from 1 to 13, with the highest values observed in populations SNK1-CM-W1, SNK2-CM-W, and SNK3-CM-W. Similarly, the number of haplotypic (*h*) varied between 2 and 8, with the highest levels observed in SNK2-CM-W and SNK3-CM-W; notably, four populations (SPB1-CM-W, SPB2-CM-W, SKW-CM-C, and PLG-CM-C) were found to be monomorphic. Although the polymorphic populations exhibited uniformly high allelic diversity (*Hd* = 1.000), the average number of nucleotide differences (*k*) showed marked spatial variation, ranging from 1.000 in UBR-CM-C, CR-CM-C, and YLA-CM-C to 7.000 in NPT-CM-W (Table S4). At the haplotypic level, the *N*_a_ and *π* across populations were 3.353 ± 0.549 and 0.008 ± 0.007, respectively, indicating a relatively low nucleotide diversity despite a moderate level of *AR* (Table 1). The mean *AR* was estimated to be 3.35 ± 2.20, while the *N*_e_ averaged 1.80 ± 0.87. The *H*_o_ and *H*_e_ heterozygosities were 0.387 ± 0.081 and 0.325 ± 0.065, respectively, representing a statistically significant difference (*p* < 0.05) across the dataset. Within the African catfish lineage, the KSN2-CG-C population exhibited the highest *H*_e_, whereas the NYK-CG-C population exhibited the lowest variation. Among the bighead catfish populations, SNK1-CM-W had the highest *H*_e_ (Table 1).

Haplotype frequencies in most populations were consistent with Hardy–Weinberg equilibrium (*p* > 0.05), although significant deviations (*p* < 0.01) were detected in populations KSN2-CG-C, SNK1-CM-W, and SNK3-CM-W. *F*_IS_ showed spatial variation, where positive values were observed for KSN2-CG-C and SNK1-CM-W, and negative values were detected in the remaining populations (Table S5). *F*_ST_ values ranged from −0.333 to 0.948, with a mean *F*_IS_ of −0.17 ± 0.19 (Table 1). AMOVA revealed that the majority of genetic variation (56%) was partitioned among populations, followed by variation among individuals (24%) and within individuals (20%) (Table S6). Notably, the overall difference between mean *H*_o_ and *H*_e_ was statistically significant, whereas pairwise comparisons at the population level remained inconclusive owing to allele fixation (*H*_o_ = 0) in several groups and the inherent limitations of analyses based on a single genetic locus (Tables S7–S9).

### 3.3. Molecular Phylogeny and Diversification Patterns of the Myf5 Locus in Clarias

**Figure 2:**
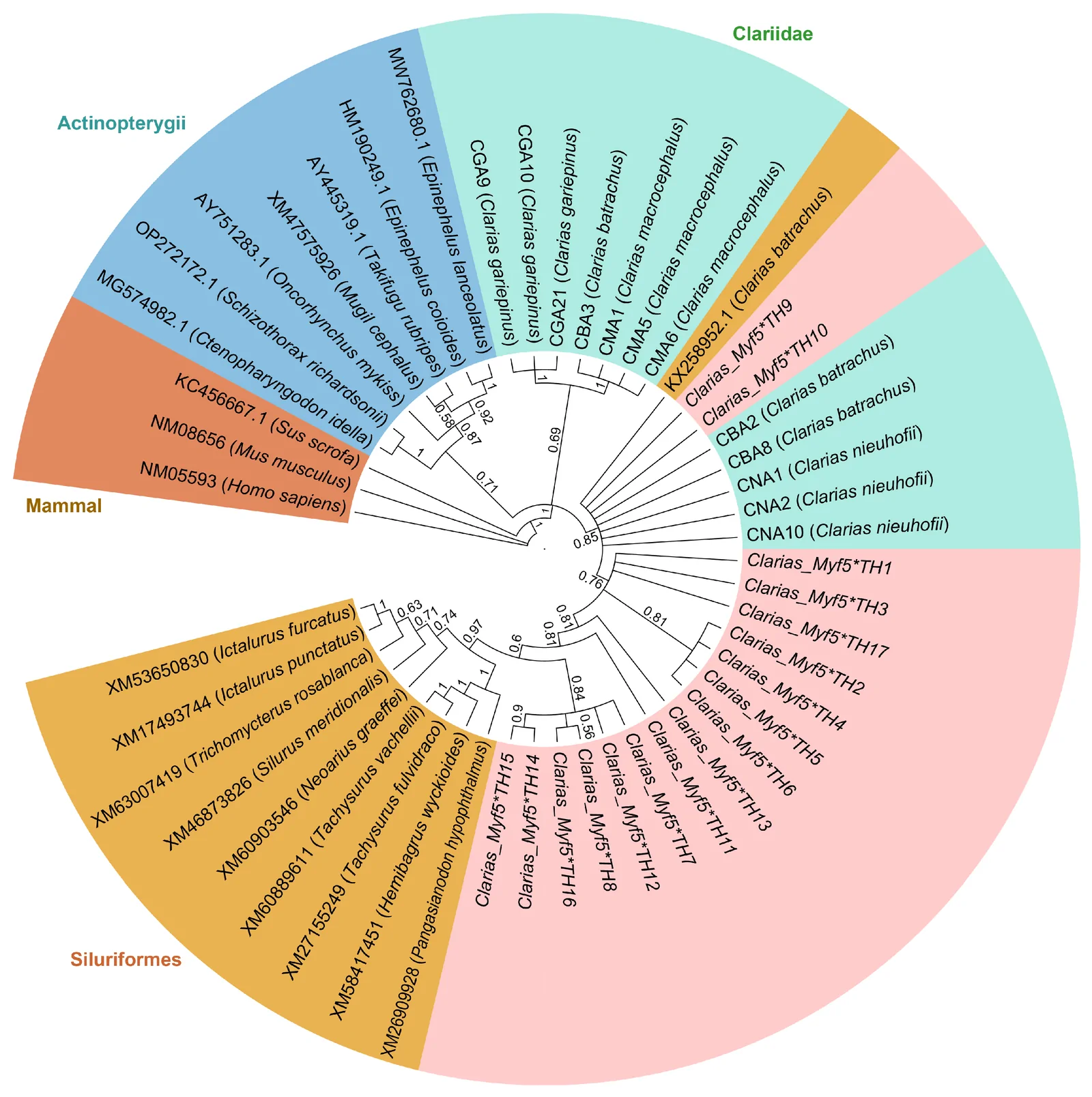
Phylogenetic tree of *Myf5* genes. This tree uses 46 fish sequences, with 3 mammalian sequences as the outgroup. Each sector represents the following: orange, mammals; blue, Actinopterygii; green, Clariidae; yellow, Siluriformes species. The target haplotype (1–17) sequences form a distinct and well-supported monophyletic group. These sequences cluster closely with other *Clarias* species, indicating a high level of genetic conservation within the genus. By contrast, non-clariid Siluriformes form separate lineages, reflecting their evolutionary divergence from *Clarias*. The numbers at the nodes indicate Bayesian posterior probabilities.

Bayesian phylogenetic inference derived from *Myf5* gene sequences (Figure 2) demonstrated that all examined specimens of the genus *Clarias* formed a robust monophyletic group (posterior probability = 1.0) that was distinctly separated from other taxa within order Siluriformes. Most internal nodes received moderate to strong posterior probability support, ranging from 0.56 to 1.00. Several haplotype groups formed highly supported subclades (PP = 0.90–1.00), including the clusters comprising Clarias_Myf5*TH2–Clarias_Myf5*TH6 and Clarias_Myf5*TH14–Clarias_Myf5*TH15, whereas other relationships were supported by moderate posterior probability values (0.56–0.85). The phylogenetic topology showed that the newly identified haplotypes were distributed across multiple lineages rather than forming a single monophyletic cluster. In addition, several haplotypes grouped closely with published *Clarias Myf5* sequences, while others formed distinct clades without previously reported homologs, suggesting the presence of lineage-specific genetic variants. No clear species-specific clustering was observed among *C. gariepinus*, *C. macrocephalus*, and *C. batrachus*, indicating a high degree of sequence conservation retention of ancestral polymorphism within the genus. Additionally, the relatively short DNA sequence analyzed may have contained insufficient phylogenetic information to clearly resolve species-specific clustering. Overall, the phylogenetic analysis demonstrated considerable genetic diversity among the identified *Myf5* haplotypes while supporting their evolutionary relationships within *Clarias*.

### 3.4. Molecular Signatures of Selective Sweeps and Adaptive Divergence at the Myf5 Locus

Selective pressures acting on the catfish *Myf5* gene were assessed using the non-synonymous-to-synonymous substitution ratio (*dN/dS*, *ω*), which consistently remained below 1 across all examined populations. This pattern, which indicates that the gene is subject to purifying selection, suggests a high degree of functional conservation in *Myf5* sequences among African, bighead, and walking catfish populations (Table 2).

**Figure 3:**
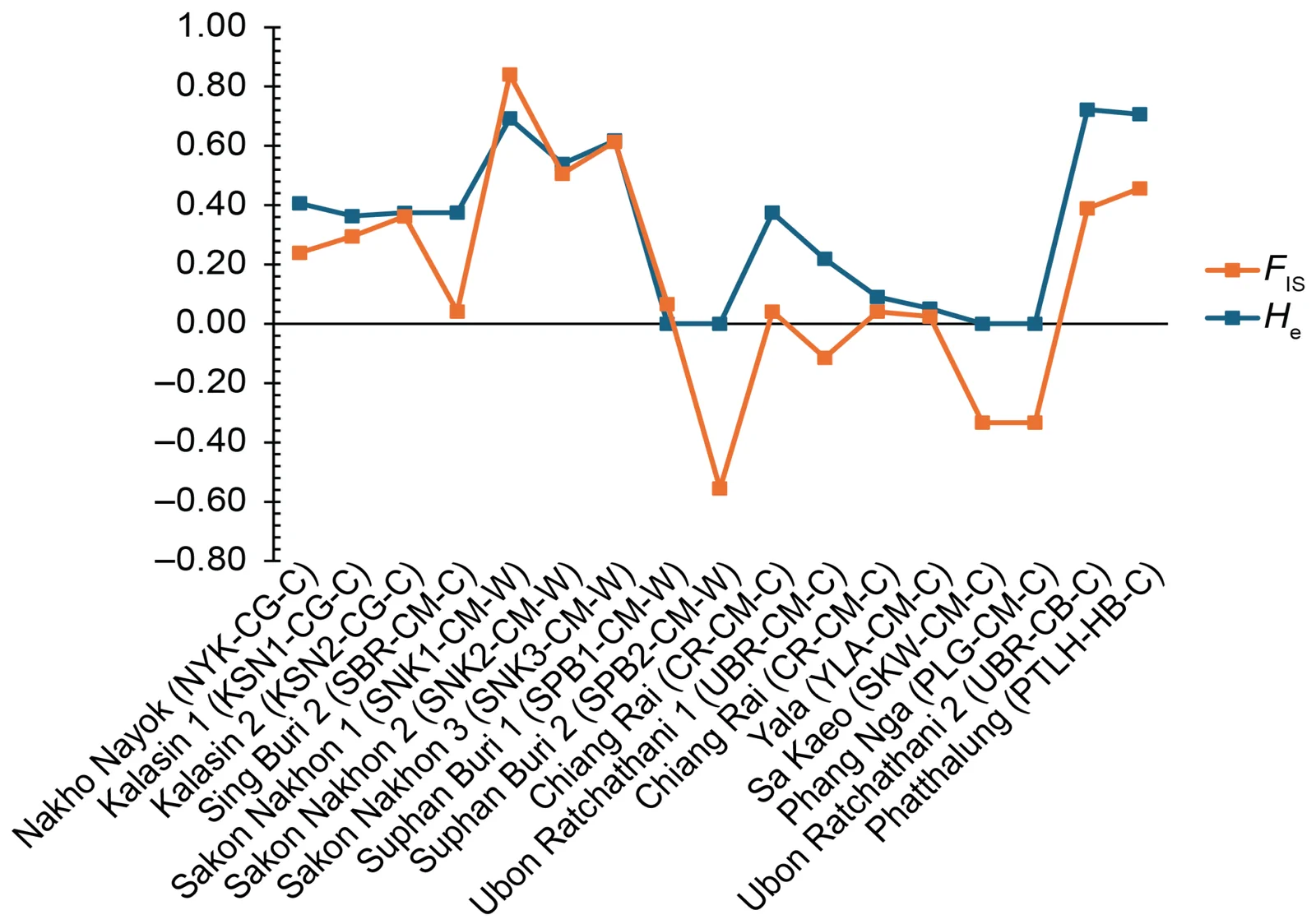
Comparison of expected heterozygosity (*H*_e_) against inbreeding coefficients (*F*_IS_) based on the *Myf5* gene for 17 catfish populations.

The mean *ω* value was estimated at 0.063 (range: 0.036–0.158), further supporting an overall trend toward purifying selection, with the highest *ω* value (0.158) observed in *C. batrachus* populations (UBR-CB-C). In nine populations, *ω* could not be estimated because of extremely low *dS* levels, which hindered reliable calculation of the ratio. Likelihood ratio testing further confirmed that the observed *ω* values were not statistically significant (LRT = 0.000, *p* = 0.500), which indicates that the locus remains largely governed by purifying selection or neutral selection (Table 2). The analysis of selective sweep signals, based on the relationship between *H*_e_ and *F*_IS_, revealed signatures of directional selection in specific populations, such as SKN1 and SPB1 (Figure 3). These populations were characterized by low *H*_e_ and elevated *F*_IS_ values, which are indicative of recent allelic fixation under selective pressure. By contrast, populations SPB1-CM-W, UBR-CM-C, and UBR-CB-C exhibited *F*_IS_ values that exceeded *H*_e_, indicating the presence of potential selective sweeps or ongoing purifying selection (Figure 3).

Neutrality tests revealed spatial heterogeneity in the genetic patterns of selection across the study area. Tajima’s *D* values ranged from −0.893 to −0.194 and were consistently negative, even though they did not reach statistical significance. Similarly, Fu and Li’s D* and F* statistics were non-significant in all examined populations. Following the application of the FDR correction for multiple comparisons, which was used to ensure statistical stringency across the diverse populations, no group exhibited statistically significant deviations (*q* > 0.05). This outcome indicates that there is no robust evidence of recent, severe population bottlenecks or strong selective sweeps that would be sufficient to override the broader purifying selection that governs this locus (Table 3).

Codon-specific selection analyses conducted using the Datamonkey platform (https://www.datamonkey.org) provided no evidence of positive selection acting on *Myf5*. The MEME model, which was optimized to detect episodic diversifying selection at individual sites, did not identify any codons undergoing transient adaptive changes (Table S10). By contrast, the FEL approach identified a specific codon position (site 52) under statistically significant purifying selection (Table S11). These findings were further corroborated by the FUBAR analysis, which revealed widespread signatures of purifying selection across most codon sites, whereas no positions showed support for diversifying selection (Table S12). These results collectively indicate that the *Myf5* gene shows a high degree of sequence conservation, reflecting the dominant influence of purifying selection throughout its evolutionary trajectory.

### 3.5. Structural Conservation and Functional Architecture of Myf5 Protein Orthologs

Comparative analysis of the 103-residue Myf5 protein sequence revealed identities ranging from 91% to 98% between the 17 haplotypes and representative species within order Siluriformes. Within the Thai catfish populations, we identified 5 missense mutations and 11 synonymous substitutions (Table S13). The highest sequence similarity (98%) was observed between the *Clarias_Myf5TH1* haplotype and *C. gariepinus* (XM_053509129), whereas the lowest similarity (91%) was observed between *Clarias_Myf5TH6* and *S. meridionalis* (XM_046873826), reflecting the substantial evolutionary divergence between these lineages (Figure S1). The high degree of sequence conservation suggests that Myf5 protein residues are under strong selective constraints across both the genus *Clarias* and related catfish taxa, indicating the preservation of essential regulatory functions. Bayesian phylogenetic reconstruction based on amino acid sequences further revealed a polyphyletic distribution of *Myf5* variants across the seven catfish species. Although multiple ancestral lineages likely contributed to the observed pattern, back-mutations to ancestral haplotypes may also have occurred and cannot be ruled out (Figure S2). Additionally, secondary structure predictions for *C. gariepinus*, *C. macrocephalus*, and *C. batrachus* indicated that α-helices and random coils are the primary structural elements that form the fundamental folding architecture of *Myf5* (Figure S3).

### 3.6. Structural Characterization and Comparative Modeling of Myf5 Protein Variants

The amino acid sequences that correspond to the *Myf5* gene exhibited high similarity to the *C. gariepinus* reference sequence (Accession No. XM053509129), with sequence identities that ranged from 91% to 98%. Comparative analysis of the 103-residue segment, which is situated within the functionally critical basic helix-loop-helix (bHLH) domain, confirmed that these *Myf5* protein segments maintain high conservation across diverse catfish lineages. The tertiary (3D) structures of the predicted peptide variants were characterized and clustered into three representative models (Figure S4), which were validated through Ramachandran plot analysis. Among these, Model 2 exhibited the highest stereochemical quality, where 76.0% of residues resided in the most favored regions, followed by Model 1 (73%) and Model 3 (72%). These results indicate that the region is evolutionarily conserved, with limited genetic variation that does not significantly alter the global protein shape, which suggests that any observed structural variance is primarily influenced by the specific amino acid composition of each haplotype. All models were structurally aligned with a reference bHLH domain (Accession: A0A8J4U0D1; Figure S5), which confirmed the functional identity of the analyzed protein region.

### 3.7. Integrative Genomic Assessment of Growth-Related Loci (Myf5, Mstnb, and GH1) for Population Subdivision

**Figure 4:**
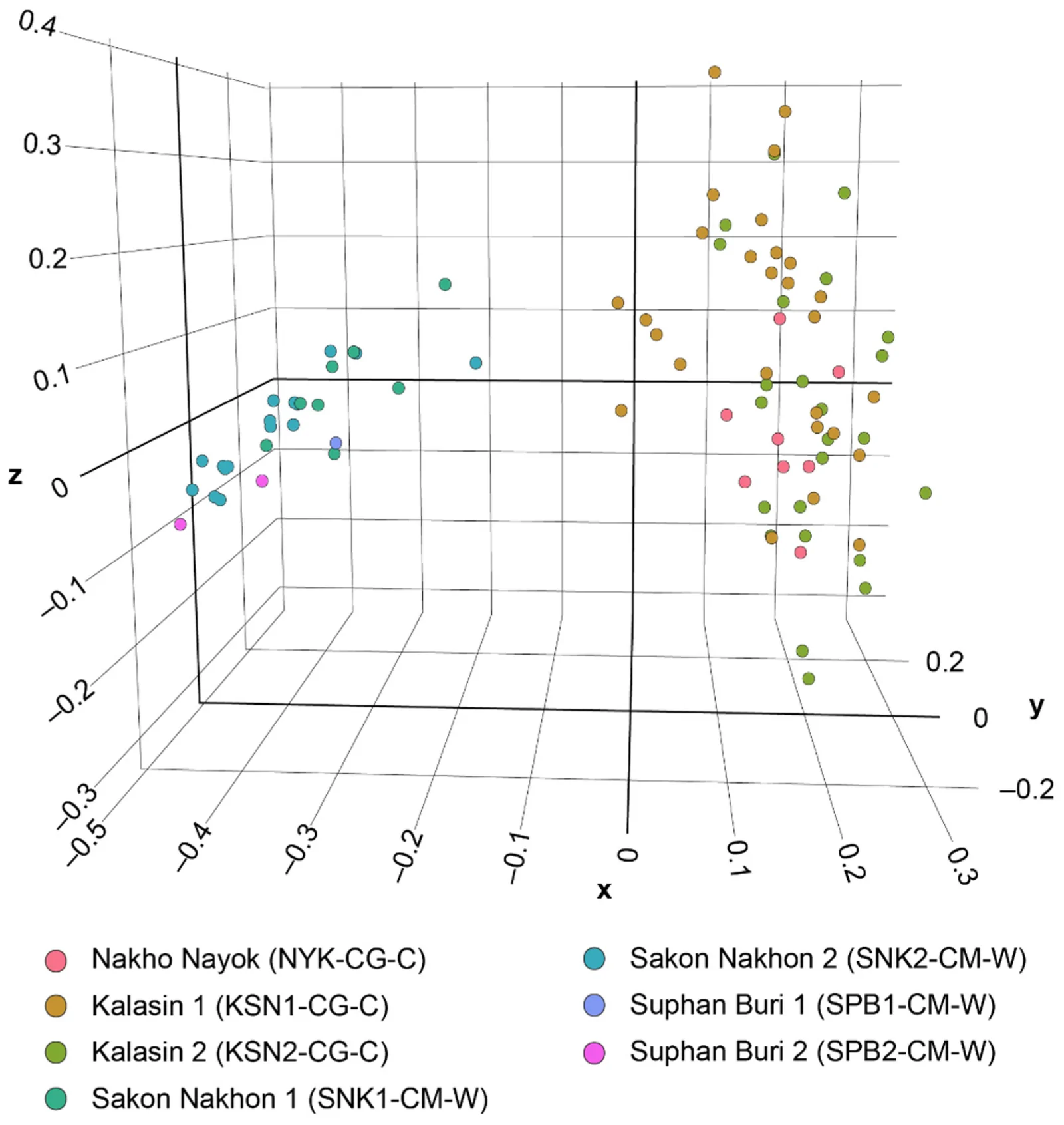
Principal component analysis (PCoA) of integrated genomic variation based on the *Myf5*, *Mstnb*, and *GH1* loci among the catfish populations in Thailand. Each population is plotted using different colors.

The PCoA and DAPC multivariate analyses of integrated multilocus genomic variation (*Myf5, Mstnb,* and *GH1*) indicated an absence of distinct individual clustering within the broader dataset, suggesting a high degree of overlapping genetic variation at the microgeographic scale. However, samples from the Sakon Nakhon and Suphan Buri populations formed a partially distinct cluster along the first principal coordinate axis, whereas the remaining populations were largely distributed on the opposite side of the ordination space, suggesting moderate geographic differentiation despite the overall weak population structure (Figure 4 and Figure S6).

**Figure 5:**
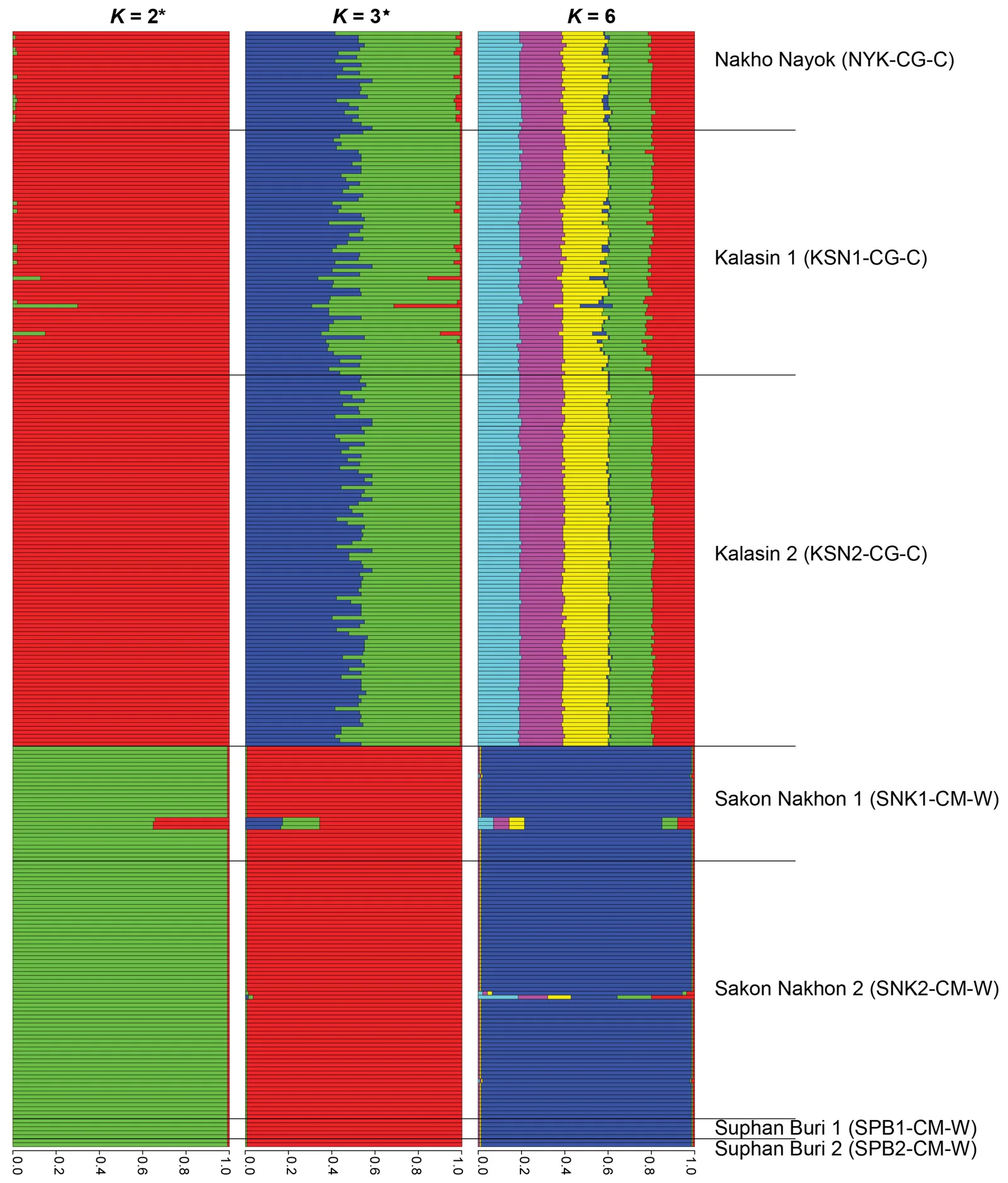
Bayesian clustering analysis of three African catfish and four bighead catfish populations in Thailand. The *x*-axis shows the proportion of membership (posterior probability) in each genetic cluster represented by different color bars, while the *y*-axis shows the individuals within each population. The optimal number of clusters was observed at *K* = 3 based on Evanno’s Δ*K* and ln Pr (X|*K*) criteria. The most probable number of K-values is represented by an asterisk (*) and star (⋆), according to Evanno’s Δ*K* and ln Pr (X|*K*) strategies, respectively.

By contrast, Bayesian clustering analysis, which utilized a multi-locus dataset incorporating *Myf5* with *Mstnb* and *GH1* sequences, identified *K* = 3 as the optimal number of clusters while consistently supporting a primary division into two major lineages (Figure 5). The *Mstnb* and *GH1* data, derived from previous genomic assessments (Accession No: NC071104; Accession No: NC071127), partitioned the samples into two distinct clades corresponding to African catfish (*C. gariepinus*) and bighead catfish (*C. macrocephalus*). Although the fundamental species-level division remained stable, hierarchical substructuring became apparent as *K* increased, enabling a highly nuanced resolution of the population architecture within each species (Figure S7). Specifically, Evanno’s Δ*K* method identified *K* = 2 as the most pronounced level of secondary structure, which reflects a strong differentiation occurring between the two primary species-clades. This hierarchical pattern, which was consistently observed across the representative sampling locations, implies that although the primary species lineages are well-preserved, localized genetic signatures begin to emerge at the sub-population level.

## 4. Discussion

Characterization of DNA sequence variation at the *Myf5* locus across 511 *Clarias* individuals from 17 populations revealed 92 variable sites and 17 haplotypes. A robust signature of purifying selection (*ω* = 0.063) underscored the functional conservation of the gene, whereas multi-locus integration with *Mstnb* and *GH1* supported hierarchical population subdivision, with *K* = 2 representing species-level differentiation and *K* = 3 representing a major secondary level of structure. Thus, the present dataset addresses the objectives of this study at complementary biological levels. Variation at the *Myf5* locus revealed species- and population-associated haplotypes, the comparison of wild and hatchery-derived samples identified uneven patterns of genetic diversity and differentiation, and the integrated multilocus analysis provided additional resolution of broad species-level and population-level structure. These findings are therefore most directly relevant to species and hybrid identification, monitoring of genetic differentiation, and future assessment of introgression between cultured and natural populations.

### 4.1. Structural Conservation and Allelic Divergence of Myf5 in Clarias Lineages

Comparative genomic analyses indicated that the *Myf5* gene in *Clarias* species retains a conserved architecture consisting of three exons and two introns. Within this framework, the 334-bp segment of exon 1 showed a high density of 92 variable sites, supporting its utility for evaluating population-level sequence variation. Although we identified 17 novel haplotypic variants, nucleotide diversity remained notably low (*π* ≈ 0.008), with only five missense mutations potentially affecting the amino acid composition. This result suggests that *Myf5* evolved under a conservative selective regime with limited adaptive diversification compared with those of other growth-related genes, such as myostatin, which exhibits higher polymorphism and lineage-specific positive selection in teleosts [60], or growth hormone genes that show greater structural variability [61]. The relative conservation of *Myf5* likely reflects its role as a developmental gatekeeper, where functional disruption may impose substantial pleiotropic constraints on embryonic myogenesis [62,63]. Haplotypic frequency distributions varied among populations, with Clarias_Myf5*TH1 occurring at high frequencies across multiple locations and species. This suggests the retention of an ancestral haplotype or its maintenance through weak positive selection under aquaculture conditions, which is known to increase the frequency of haplotypes associated with desirable traits, such as growth performance [64,65]. However, population-level frequencies alone are insufficient to distinguish between ancestral retention and adaptive significance without direct evidence of fitness. Haplotype distributions differed among the sampled taxa. Within the present Thai dataset, two haplotypes were observed for *C. gariepinus*, five were observed for *C. macrocephalus*, and one (*Clarias_Myf5TH10*) was observed for *C. batrachus*. These exclusive occurrences indicate candidate taxon-associated variation, but should not be interpreted as definitive species-specificity. In particular, because *C. batrachus* and the hybrid group were each represented by only one population, haplotypes detected exclusively in these samples may reflect population-level or local variation rather than species-wide differentiation. Haplotypes confined to a single sampling locality should therefore be regarded as population-specific within the present dataset. Broader and more balanced sampling across Thailand and other parts of the species’ ranges will be required to determine whether these variants are consistently associated with particular taxa or are geographically restricted. Accordingly, these haplotypes should currently be considered candidate components of a multi-locus panel for broodstock identification and hybrid monitoring rather than validated species diagnostic markers [66,67]. These diagnostic variants provide useful molecular markers that may assist hatchery managers in preserving the genetic integrity of native broodstock and supporting the sustainable management of clariid resources.

The observed genetic structure also reflects the unique history of catfish aquaculture in Thailand. The clear separation among *C. gariepinus*, *C. macrocephalus*, and *C. batrachus*, together with the hierarchical subdivision revealed by the multilocus analyses, suggests that species identity remains largely preserved despite decades of intensive hatchery production and hybridization. At the phylogeographic scale, these patterns indicate that evolutionary divergence among species remains the dominant source of genetic differentiation, whereas localized substructure within *C. macrocephalus* probably reflects restricted broodstock exchange and regional demographic history. From an aquaculture perspective, the species-specific *Myf5* haplotypes identified here provide practical molecular markers for broodstock authentication, hybrid verification, and monitoring unintended introgression in hatchery stocks. These findings also have direct implications for fisheries management and conservation in Thailand by supporting the maintenance of genetically distinct broodstock, for monitoring the movement of hatchery fish into natural populations, and preserving the genetic integrity of native *C. macrocephalus* and *C. batrachus*, which may be increasingly threatened by extensive hybrid production involving *C. gariepinus*.

### 4.2. Pervasive Purifying Selection and Structural Constraints Preserve the Functional Integrity of the Myf5 Myogenic Regulator

The results of the selection analyses were consistent with purifying selection acting on the analyzed *Myf5* exon 1 region, which is evidenced by a consistently low *dN*/*dS* ratio (*ω*), averaging 0.063 across all examined populations. This pattern is consistent with evolutionary conservation of *Myf5*, a key regulator of myogenesis that initiates the specification and commitment of myogenic precursor cells during early somitogenesis. In the myogenic regulatory cascade, Myf5 functions as an early transcriptional activator that acts upstream of MyoD and downstream of Pax3/Pax7 [68,69]. As any non-synonymous substitution in such a critical developmental gatekeeper can be deleterious, the observed genetic patterns suggest that most mutations are removed from the population by selection to preserve the regulatory efficacy of the protein. Detailed codon-based analyses performed using the Datamonkey platform further resolved this selective landscape, in which the FEL and FUBAR models detected pervasive purifying selection across most nucleotide sites. Specifically, FEL analysis identified site 52 as being under statistically significant purifying selection, suggesting strong functional constraints within the basic helix-loop-helix (bHLH) domain and adjacent regions (Table S12). Secondary structure predictions and 3D modeling using AlphaFold 3 suggested that site 52 is located within a conserved α-helix that forms the protein’s core folding architecture. The high stereochemical quality of these models, which was validated through Ramachandran analysis, indicates that helix stability is essential for the dimerization and DNA-binding functions of the bHLH motif [70]. Moreover, structural alignment with a reference domain (A0A8J4U0D1) confirmed that the limited variation across lineages does not disrupt the global protein shape. This preservation of the tertiary architecture underscores the effect of robust purifying selection that maintains the regulatory integrity of *Myf5* during catfish myogenesis. This high level of conservation in exon 1 is consistent with the patterns observed in other teleosts, where functionally important domains remain highly conserved, whereas neutral divergence accumulates in non-critical regions [66,71]. This predominantly neutral-to-purifying evolutionary pattern highlights the functional distinction between upstream developmental regulators that establish a myogenic lineage and downstream effector genes that are more responsive to domestication-driven selection [72]. For example, myostatin paralogs (*Mstn1* and *Mstn2*) in teleost fishes often exhibit diversifying selection associated with interspecific variation in muscle growth, whereas *Myf5* remains influenced by purifying selection [73,74]. Despite the lack of locus-wide positive selection (Table S11), some populations exhibited reduced heterozygosity and elevated *F*_IS_ values, patterns potentially consistent with demographic effects or localized selection such as in SNK1-CM-W and Kalasin-derived samples. These patterns, characterized by reduced *H*_e_ and elevated *F*_IS_, are consistent with intensive breeding practices in major Thai hatchery systems where directional selection for growth has been applied across multiple generations [75,76].

From a functional perspective, the five missense mutations detected, including the Ser52Pro substitution, warrant further investigation (Table S14). Because proline residues can influence α-helical conformations, the Ser52Pro substitution may warrant future functional investigation. This change could subtly alter the folding dynamics of the protein, potentially affecting the transcriptional regulation of downstream targets such as myogenin [77]. Moreover, our analysis of hybrid individuals revealed only parental haplotypes, which reflects the controlled production of first-generation hybrids in aquaculture systems where heterosis is exploited without establishing new lineages. These findings further emphasize the importance of maintaining genetically diverse broodstock, particularly because the use of limited numbers of broodstock in selective breeding programs can reduce genetic diversity. Although *Myf5* is unlikely to represent a major quantitative trait locus for the growth differences between *C. gariepinus* and *C. macrocephalus*, its allelic variation contributes to the genomic resources available for monitoring genetic health and managing broodstock diversity.

### 4.3. High-Resolution Population Architecture and the Efficacy of Multi-Locus Integration

The integration of *Myf5* genotypes with multi-locus data substantially improved the resolution of the population structure in *Clarias* when compared with that obtained using single-locus analyses. AMOVA showed that 56% of the genetic variation was partitioned among populations, indicating significant isolation or species-level divergence despite microgeographic overlaps. This elevated differentiation exceeds the typical 20–40% reported for the microsatellite and SNP datasets in *Clarias* [75,78], which likely reflects high variability at the *Myf5* locus, the consideration of variation at the haplotypic level, and the combined effects of strong purifying selection that maintains species-specific functional haplotypes and limited introgression at this developmental locus. *Myf5* is a robust candidate diagnostic marker for identifying parental origin and first-generation hybrids, consistent with the utility of conserved nuclear loci in other fish taxa [79,80]. Bayesian clustering identified *K* = 2 as the optimal partition, which corresponded to the primary divergence between *C. gariepinus* and *C. macrocephalus*, whereas *K* = 3 revealed finer substructures associated with geographic origin and hatchery history. This bipartite structure aligns with the deep phylogenetic separation of these species, estimated to date back to the Miocene by molecular clock analyses [81,82]. Additionally, the consistently negative Tajima’s D*, and F* statistics, which remained statistically non-significant (*q* > 0.05) after FDR correction, suggest that the observed genetic patterns result from demographic processes, such as population expansion or genetic drift, rather than localized adaptive evolution. The absence of significant *q*-values further indicates that no recent bottlenecks or selective sweeps were strong enough to override the pervasive purifying selection that governs the *Myf5* locus, which effectively maintains the genomic stability and functional integrity of this myogenic regulator across Thai clariid populations. Despite the strong interspecific resolution, PCoA and DAPC revealed a substantial overlap among individuals, particularly in hatchery-derived populations. This pattern is consistent with the extensive use of hybridization between *C. macrocephalus* and *C. gariepinus* in Thai aquaculture, which dominates national production [76]. The distinct clustering of *C. batrachus* across Bayesian and ordination analyses represents a key outcome of this multi-locus approach, where clear genetic separation highlights a specific vulnerability to introgression from numerically dominant farmed lineages [83]. These findings support the development of a standardized multi-locus genotyping panel incorporating *Myf5* and additional nuclear markers, which would enhance species assignment accuracy and hybrid detection [84,85]. In aquaculture, such tools could enable the verification of broodstock identity and detection of unauthorized hybridization [75]. In the context of conservation, these markers would facilitate landscape-scale monitoring of gene flow from farmed to wild populations, enabling the early detection of introgression and supporting the maintenance of the genetic integrity of native populations. Such information could also help identify conservation priorities and guide broodstock management to preserve genetic diversity. In aquaculture, implementation of cost-effective amplicon sequencing can support routine genomics-based monitoring for species identification, broodstock authentication, hybrid verification, and the maintenance of genetic diversity in selective breeding programs [86,87].

### 4.4. Genomic Applications and Study Limitations for Clariid Management

The results of this study have significant implications for the sustainability of the Thai catfish industry, which is currently facing challenges related to reduced genetic diversity and stock homogenization. The elevated *F*_IS_ values observed in specific populations, such as SNK1-CM-W (0.148), highlight the immediate risk of random genetic drift and inbreeding depression that often results from reliance on limited broodstock lines in intensive hatchery systems. To address these issues, the species-diagnostic haplotypes identified at the *Myf5* locus, such as the unique *Clarias_Myf5TH10* variant in *C. batrachus*, provide a practical basis for developing low-cost molecular tools. Haplotype-specific PCR assays based on these variants can be implemented using standard laboratory equipment to enable routine verification of species identity and hybrid status [79,87,88]. These approaches address persistent issues of mislabeling and can be integrated with traceability systems under certification frameworks, such as the Aquaculture Stewardship Council [89]. Moreover, the 17 *Myf5* haplotypes identified here could be evaluated as components of multi-locus panels for species identification, broodstock verification, and hybrid monitoring. Their relevance to growth performance remains unknown because no phenotypic measurements or genotype–phenotype association analyses were included in the present study. The present study demonstrates haplotypic differentiation among taxa and populations but it did not test associations between *Myf5* genotypes and growth rate, body weight, feed efficiency, disease resistance, or other economically important traits. Therefore, the identified haplotypes should currently be regarded as candidate diagnostic or stock-management markers rather than markers for trait-based selection. From a conservation perspective, our findings indicate the ongoing erosion of native *Clarias* genetic diversity, where the clear genetic distinctiveness of *C. macrocephalus* and *C. batrachus* emphasizes their vulnerability to genetic swamping from hybrid escapees. Therefore, conservation strategies should prioritize the effective confinement of hybrid stocks in aquaculture operations to prevent escape and introgression into natural populations, while also combining the protection of natural populations with ex-situ approaches, such as gene banking and cryopreservation, to preserve rare haplotypes at functionally relevant loci [90,91]. Using these diagnostic variants, the genetic purity of native stocks that are increasingly being displaced by numerically dominant cultured lineages can be protected.

Several limitations constrain the interpretation of these results. First, this analysis focused on a partial 334-bp segment of exon 1, which excludes regulatory elements and non-coding regions that may play significant roles in gene expression. Second, the absence of corresponding phenotypic data prevents the direct assessment of genotype–phenotype relationships, limiting our ability to make definitive functional inferences regarding growth performance. Third, the hybrid sampling was restricted to a single population, which may not have captured the full diversity of hybridization practices across different Thai aquaculture systems [92]. It should be noted that the present study focused on sequence variation within a candidate functional gene rather than genome-wide neutral markers. Consequently, the observed population structure reflects variation at the investigated loci and should not be interpreted as representing genome-wide diversity. Future studies integrating genome-wide SNP datasets with functional candidate genes, including members of the MRF gene family, will provide a more comprehensive understanding of population history, adaptive differentiation, and introgression in Thai catfish populations.

An additional limitation concerns the geographic scope and uneven taxonomic representation of the sampling design. All specimens were collected in Thailand, with 12 populations of *C. macrocephalus* and three populations of *C. gariepinus*, but only one population each of *C. batrachus* and hybrid catfish. Consequently, haplotype frequencies, private haplotypes, diversity indices, population structure, and the apparent diagnostic value of individual *Myf5* variants should be regarded as a baseline for Thai populations rather than as range-wide estimates. The strong functional constraint inferred for *Myf5* may be less geographically dependent than the population-level patterns because *Myf5* is a conserved regulator of myogenesis; nevertheless, this interpretation also requires direct validation using non-Thai *Myf5* sequences. Regional studies using other marker systems demonstrate that clariid population patterns can differ substantially among countries. *C. macrocephalus* populations from the Vietnamese Mekong Delta showed relatively high diversity and gene flow, whereas comparisons among Cambodia, Viet Nam, and Peninsular Malaysia revealed pronounced regional structures and distinct evolutionary units [93,94,95]. Studies in Viet Nam also reported little evidence of introgression from *C. gariepinus* into native *C. macrocephalus* [96], contrasting with previous observations from Thailand. Similarly, mitochondrial analyses of *C. batrachus* across Southeast Asia identified geographically structured lineages and region-specific haplotypes. These comparisons indicate that regional aquaculture practices, broodstock movement, drainage connectivity, and demographic history may influence genetic patterns. Because the published studies used microsatellites, *ISSR* markers, or mitochondrial sequences rather than *Myf5*, direct numerical comparisons among diversity indices are not appropriate. Balanced sampling from neighboring countries and other parts of the species’ ranges will therefore be necessary to determine whether the *Myf5* haplotypes identified here are geographically restricted or consistently diagnostic across Southeast Asia.

Future research should thus prioritize a comprehensive characterization of the *Myf5* gene through whole-gene resequencing and genome-wide association studies in phenotyped populations to identify quantitative trait loci for growth [86,87]. Additionally, long-term monitoring of wild populations using standardized genotyping panels is essential to quantify introgression dynamics and support evidence-based management of hybrid impacts on native genetic resources [97,98]. Although mitochondrial markers such as *COI*, *Cyt b*, or *16S* rRNA are widely used for phylogeographic reconstruction and species identification, they only reflect maternal inheritance and therefore provide limited resolution for detecting hybrid ancestry. Future studies integrating mitochondrial DNA with genome-wide nuclear markers will provide a more comprehensive understanding of both maternal lineage history and biparental introgression across Southeast Asian *Clarias* populations.

## 5. Conclusions

This study successfully characterized the genomic architecture and allelic diversity of the *Myf5* exon 1 region across 511 individuals, providing high-resolution data for evaluating the distribution of genetic diversity within Thai clariid populations. Our findings support the primary hypothesis that although the *Myf5* gene is subjected to stringent purifying selection (*ω* = 0.063) to preserve its essential myogenic regulatory function, it harbors sufficient population-specific polymorphisms to differentiate between native species and their hybrids. The identification of 92 variable sites and 17 novel haplotypes demonstrated that lineage-level diversification and neutral divergence coexist with intense functional constraints that maintain the structural integrity of the protein’s α-helix folding architecture. The resolution of a hierarchical population structure (*K* = 3) and partitioning of 56% of the genetic variation among populations confirmed that *Myf5* variation effectively reflects deep evolutionary divisions, where *C. gariepinus*, *C. macrocephalus*, and *C. batrachus* remain genetically distinct. From an applied perspective, the discovery of species-diagnostic haplotypes establishes *Myf5* as a robust molecular marker for monitoring genetic introgression and detecting first-generation hybrids, which are critical requirements for the sustainable management of Thai aquaculture resources. Although the predominantly neutral-to-purifying pattern of selection suggests that *Myf5* is unlikely to be a major direct driver of interspecific growth differences, its utility in marker-assisted selection and the detection of localized inbreeding provides a vital genomic baseline. Overall, this study provides candidate diagnostic tools for species, hybrid, and broodstock management, but does not establish *Myf5* variants as markers for growth-related selection. Beyond providing a genomic resource, the present dataset establishes a baseline for monitoring genetic integrity in Thai clariid populations. These findings support evidence-based broodstock management, conservation planning, and sustainable aquaculture by facilitating the identification of species-specific lineages and genetic introgression.

## Figures and Tables

**Table 1:** Nucleotide sequence diversity in catfish populations based on *Myf5* sequences.

Species	Population	Code	*N* ^1^	*N*_a_ ^2^	*N*_e_ ^3^	*AR* ^4^	*H*_o_ ^5^	*H*_e_ ^6^	*F* ^7^	*F*_IS_ ^8^	Π ^9^	HWE ^10^
*C. gariepinus*	Nakho Nayok	NYK-CG-C	26	3.000	1.68	3.00	0.500	0.406	−0.231	−0.17	0.009 ^ns^	0.409 ^ns^
	Kalasin 1	KSN1-CG-C	70	5.000	1.57	5.00	0.429	0.363	−0.180	−0.07	0.009 ^ns^	0.877 ^ns^
	Kalasin 2	KSN2-CG-C	95	5.000	1.60	5.00	0.368	0.374	0.015	−0.01	0.005 ^ns^	0.001 **
	Mean	-	185	4.333 ± 0.943	1.62 ± 0.05	4.33 ± 0.94	0.432 ± 0.054	0.381 ± 0.018	−0.132 ± 0.106	−0.08 ± 0.06	0.008 ± 0.002	-
*C. macrocephalus*	Sing Buri 2	SBR-CM-C	2	2.000	1.60	2.00	0.500	0.375	−0.333	−0.33	0.014 ^ns^	0.637 ^ns^
	Sakon Nakhon 1	SNK1-CM-W	30	6.000	3.26	6.00	0.567	0.693	0.182	0.15	0.016 ^ns^	0.001 **
	Sakon Nakhon 2	SNK2-CM-W	136	7.000	2.17	7.00	0.596	0.539	−0.105	−0.03	0.015 ^ns^	0.677 ^ns^
	Sakon Nakhon 3	SNK3-CM-W	63	8.000	2.62	8.00	0.730	0.618	−0.181	−0.01	0.014 ^ns^	0.001 **
	Suphan Buri 1	SPB1-CM-W	5	1.000	1.00	1.00	0.000	0.000	N/A	0.07	-	-
	Suphan Buri 2	SPB2-CM-W	3	1.000	1.00	1.00	0.000	0.000	N/A	−0.56	-	-
	Nakhon Pathum	NPT-CM-W	2	2.000	1.60	2.00	0.500	0.375	−0.333	−0.33	0.021 ^ns^	0.637 ^ns^
	Ubon Ratchathani 1	UBR-CM-C	4	2.000	1.28	2.00	0.250	0.219	−0.143	−0.33	0.003 ^ns^	0.174 ^ns^
	Chiang Rai	CR-CM-C	21	2.000	1.10	2.00	0.095	0.091	−0.050	−0.05	0.003 ^ns^	0.819 ^ns^
	Yala	YLA-CM-C	19	2.000	1.05	2.00	0.053	0.051	−0.027	−0.03	0.003 ^ns^	0.906 ^ns^
	Sa Kaeo	SKW-CM-C	18	1.000	1.00	1.00	0.000	0.000	N/A	−0.33	-	-
	Phang Nga	PLG-CM-C	3	1.000	1.00	1.00	0.000	0.000	N/A	−0.33	-	-
	Mean	-	306	2.917 ± 2.431	1.56 ± 0.72	2.92 ± 2.43	0.274 ± 0.271	0.247 ± 0.252	−0.124 ± 0.158	−0.18 ± 0.21	0.007 ± 0.008	-
*C. batrachus*	Ubon Ratchathani 1	UBR-CB-C	3	4.000	3.60	4.00	1.000	0.722	−0.385	−0.33	0.010 ^ns^	0.775 ^ns^
Hybrid catfish	Phatthalung	PTLH-HB-C	11	5.000	3.41	5.00	1.000	0.707	−0.415	−0.25	0.016 ^ns^	0.138 ^ns^
	Overall mean value	-	511	3.353 ± 2.195	1.80 ± 0.87	3.35 ± 2.20	0.387 ± 0.325	0.325 ± 0.260	−0.168 ± 0.158	−0.17 ± 0.19	0.008 ± 0.007	-

^1^ Number of individuals (*N*); ^2^ number of different alleles (*N*_a_); ^3^ number of effective alleles (*N*_e_); ^4^ expected heterozygosity (*H*_e_); ^5^ observed heterozygosity (*H*_o_); ^6^ allelic richness per locus and population (*AR*); ^7^ fixation index (*F*); ^8^ inbreeding coefficient related to subpopulations (*F*_IS_); ^9^ nucleotide diversity (*π*); ^10^ Hardy–Weinberg Equilibrium (HWE); ** *p* < 0.001; ^ns^ not significant.

**Table 2:** Rates of synonymous (*d*_S_) and non-synonymous (*d*_N_) substitutions in nucleotide sequences of the *Myf5* gene in catfish populations.

Species	Population	Code	*N* ^1^	*dS* (±SE)	*dN* (±SE)	*ω* (*dN*/*dS*)	LRT ^2^	*p*-Value
*C. gariepinus*	Nakho Nayok	NYK-CG-C	26	0.010 ± 0.005	0.000 ± 0.000	-	0.000	0.5
	Kalasin 1	KSN1-CG-C	70	0.016 ± 0.007	0.001 ± 0.001	0.063	0.000	0.5
	Kalasin 2	KSN2-CG-C	95	0.007 ± 0.005	0.000 ± 0.000	-	0.000	0.5
	Mean	-	185	0.011 ± 0.004	0.001 ± 0.000	0.091	0.000	0.5
*C. macrocephalus*	Sing Buri 2	SBR-CM-C	2	0.055 ± 0.023	0.002 ± 0.003	0.036	0.000	0.5
	Sakon Nakhon 1	SNK1-CM-W	30	0.028 ± 0.010	0.002 ± 0.001	0.071	3.816	0.074
	Sakon Nakhon 2	SNK2-CM-W	136	0.015 ± 0.007	0.001 ± 0.001	0.067	2.673	0.131
	Sakon Nakhon 3	SNK3-CM-W	63	0.023 ± 0.009	0.002 ± 0.001	0.087	4.301	0.058
	Suphan Buri 1	SPB1-CM-W	5	0.000 ± 0.000	0.000 ± 0.000	-	-	-
	Suphan Buri 2	SPB2-CM-W	3	0.000 ± 0.000	0.000 ± 0.000	-	-	-
	Nakhon Pathum	NPT-CM-W	2	0.052 ± 0.021	0.003 ± 0.003	0.058	-	-
	Ubon Ratchathani 1	UBR-CM-C	4	0.005 ± 0.005	0.000 ± 0.000	-	-	-
	Chiang Rai	CR-CM-C	21	0.000 ± 0.000	0.001 ± 0.001	-	-	-
	Yala	YLA-CM-C	19	0.000 ± 0.000	0.001 ± 0.001	-	-	-
	Sa Kaeo	SKW-CM-C	18	0.000 ± 0.000	0.000 ± 0.000	-	-	-
	Phang Nga	PLG-CM-C	3	0.000 ± 0.000	0.000 ± 0.000	-	-	-
	Mean	-	306	0.015 ± 0.020	0.001 ± 0.001	0.067	5.102	0.038
*C. batrachus*	Ubon Ratchathani 1	UBR-CB-C	3	0.019 ± 0.011	0.003 ± 0.002	0.158	0.000	0.5
Hybrid catfish	Phatthalung	PTLH-HB-C	11	0.050 ± 0.019	0.002 ± 0.002	0.040	0.000	0.5
	Overall mean value	-	511	0.016 ± 0.019	0.001 ± 0.001	0.063	4.731	0.046

^1^ Number of individuals (*N*); ^2^ Likelihood Ratio Test (*LRT*).

**Table 3:** The results of neutrality test for the *Myf5* gene in catfish populations.

Species	Population	Code	Tajima’s *D*	Fu and Li’s *D*	Fu and Li’s *F*
*C. gariephinus*	Nakhon Nayok	NYK-CG-C	−0.809 ^ns^	−0.809 ^ns^	−0.777 ^ns^
	Kalasin 1	KSN1-CG-C	−0.809 ^ns^	−0.809 ^ns^	−0.777 ^ns^
	Kalasin 2	KSN2-CG-C	−1.893 ^ns^	−1.893 ^ns^	−1.611 ^ns^
	Mean	-	−0.191 ^ns^	−0.191 ^ns^	−0.198 ^ns^
*C. macrocephalus*	Sing Buri	SB-CM-C	−0.687 ^ns^	−0.687 ^ns^	−0.676 ^ns^
	Sakon Nakhon 1	SNK1-CM-W	−0.564 ^ns^	0.789 ^ns^	−0.802 ^ns^
	Sakon Nakhon 2	SNK2-CM-W	−0.724 ^ns^	0.578 ^ns^	−0.679 ^ns^
	Sakon Nakhon 3	SNK3-CM-W	−0.194 ^ns^	−0.194 ^ns^	−0.117 ^ns^
	Suphan Buri 1	SPB1-CM-W	-	-	-
	Suphan Buri 2	SPB2-CM-W	-	-	-
	Nakhon Pathom	NPT-CM-W	-	-	-
	Ubon Ratchathani 1	UBR-CM-C	-	-	-
	Chiang Rai	CR-CM-C	-	-	-
	Yala	YLA-CM-C	-	-	-
	Sa Kaeo	SKW-CM-C	-	-	-
	Phang Nga	PLG-CM-C	-	-	-
	Mean	-	−0.051 ^ns^	−0.381 ^ns^	−0.335 ^ns^
*C*. *batrachus*	Ubon Ratchathani 2	UBR-CB-C	−0.956 ^ns^	−0.956 ^ns^	−0.904 ^ns^
Hybrid	Phatthalung	PTLH-HB-C	−0.895 ^ns^	−0.895 ^ns^	−0.943 ^ns^
	Overall mean value	-	−0.263 ^ns^	−0.189 ^ns^	−0.071 ^ns^

^ns^, not significant. Statistical significance evaluated at *q* < 0.05 following Benjamini–Hochberg false discovery rate (FDR) correction. No significant deviations were observed.

## Data Availability

All sequences were deposited in the DNA Data Bank of Japan (DDBJ) (https://www.ddbj.nig.ac.jp/) (accession numbers: LC928348_LC928364, deposited on 21 April 2026).

## Supplementary Materials

The following supporting information can be downloaded at: https://www.mdpi.com/article/10.3390/genes17080920/s1, Figure S1: Alignment of Myf5 protein sequences of catfish populations in Thailand and the reference sequences; Figure S2: Bayesian phylogenetic tree based on Myf5 amino acid residues in catfish species; Figure S3: Secondary structure prediction of Myf5 protein from catfish species in Thailand; Figure S4: Alignment of amino acid sequences of *Myf5* gene haplotypes from exon 1 with 3D structures of their basic helix-loop-helix (bHLH) domain retrieved from UniProt database; Figure S5: Alignment of amino acid sequences of exon 1 of the Myf5 gene haplotypes with the reference sequences from Uniprot (A0A8J4U0D1) and *C. gariepinus* (XP_053365104); Figure S6: Discriminant analysis of principal components (DAPC) of catfish populations in Thailand. Each population is plotted using different colors; Figure S7: Different population structure patterns of catfish populations in Thailand generated by model-based Bayesian clustering algorithms implemented in STRUCTURE based on (A) plot of Evanno’s Δ*K* and (B) plot of Pr(X|*K*) strategy; Table S1: Summary of catfish individuals sampled in this study; Table S2: Catfish specimens used in this study; Table S3: List of teleost and *Myf5*-related sequences retrieved from public databases for comparative genomic analysis; Table S4: Variable sites in sequences of *Myf5* haplotypes from catfish populations in Thailand; Table S5: Genetic diversity indices of catfish populations in Thailand based on *Myf5* sequences; Table S6: Genetic differentiation among catfish populations in Thailand based on *Myf5* sequences; Table S7: Analysis of molecular variance (AMOVA) results for catfish populations in Thailand; Table S8: Comparison of observed (*H*_o_) and expected heterozygosity (*H*_e_) of the *Myf5* gene in catfish populations; Table S9: Comparison of the expected heterozygosity (*H*_e_) of the *Myf5* gene between catfish populations; Table S10: Comparison of the observed heterozygosity (*H*_o_) of the *Myf5* gene between catfish populations; Table S11: Detailed site-by-site results from the Mixed-Effects Model of Evolution (MEME) analysis; Table S12: Detailed site-by-site results from the Fixed-Effects Likelihood (FEL) analysis; Table S13: Codon sites under selection identified using Fast, Unconstrained Bayesian AppRoximation (FUBAR) analysis; Table S14: Types of single-nucleotide polymorphism (SNPs) and their locations in the partial fragment of the exon 1 of *Myf5* gene of catfish populations in Thailand compared with the reference sequence (Accession number XM053509129).

## Abbreviations

The following abbreviations are used in this manuscript:

DAPC	Discriminant Analysis of Principal Components
FEL	Fixed-Effects Likelihood
FUBAR	Fast Unconstrained Bayesian Approximation
K2P	Kimura Two-Parameter model
ML	Maximum Likelihood
MEME	Mixed-Effects Model of Evolution
*Myf5*	Myogenic factor 5
PCoA	Principal Coordinate Analysis
PCR	Polymerase Chain Reaction
