# Supplementary material for: Purifying Selection and Interspecific Differentiation at the Myf5 Locus Inform Hybrid Identification and Genetic Management of Thai Clariid Resources
Source: Genes (Basel). 2026 Aug 4;17(8):920. doi: 10.3390/genes17080920 (PMC13512027; doi:10.3390/genes17080920)
Supplement: Supplementary file 1 [file genes-17-00920-s001.zip › genes-4429654-supplementary.pdf]

Supplementary Legends

Supplementary Figure

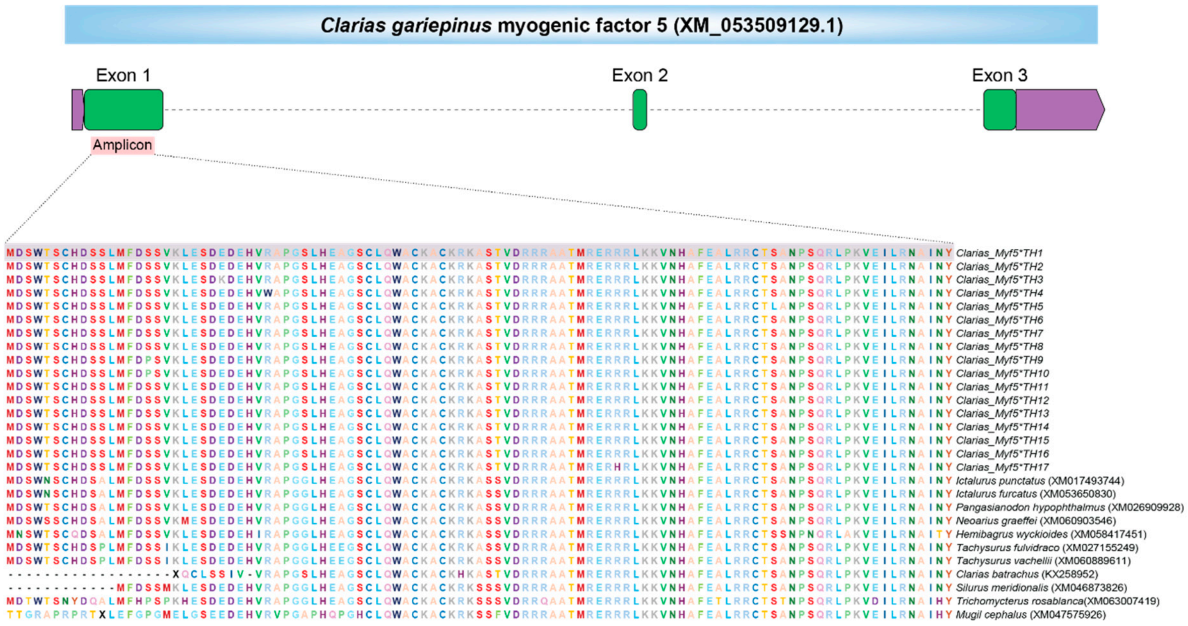

Figure S1 *Myf5* protein sequence alignment of the catfish populations in Thailand and reference sequences

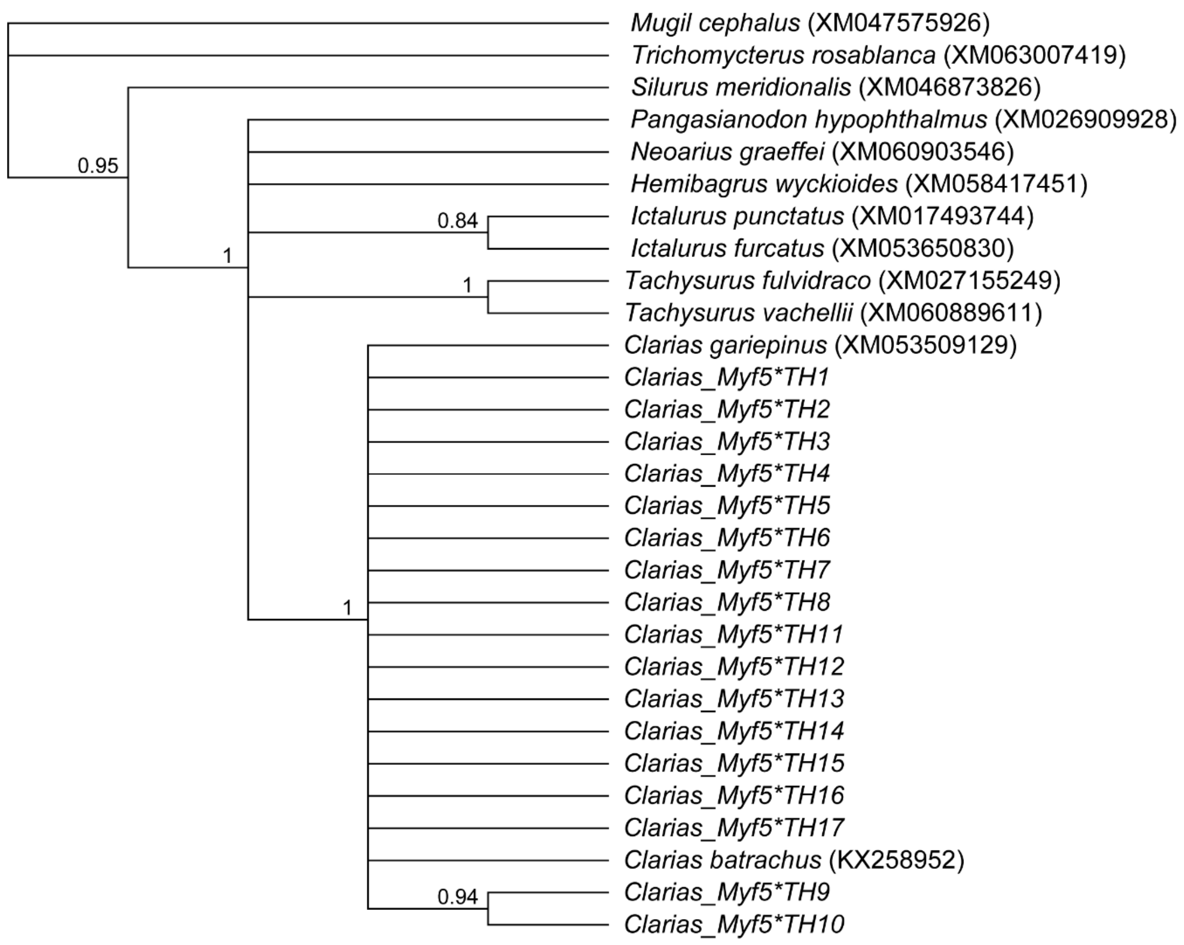

Figure S2 Bayesian phylogenetic tree based on *Myf5* gene amino acid residues in catfish species

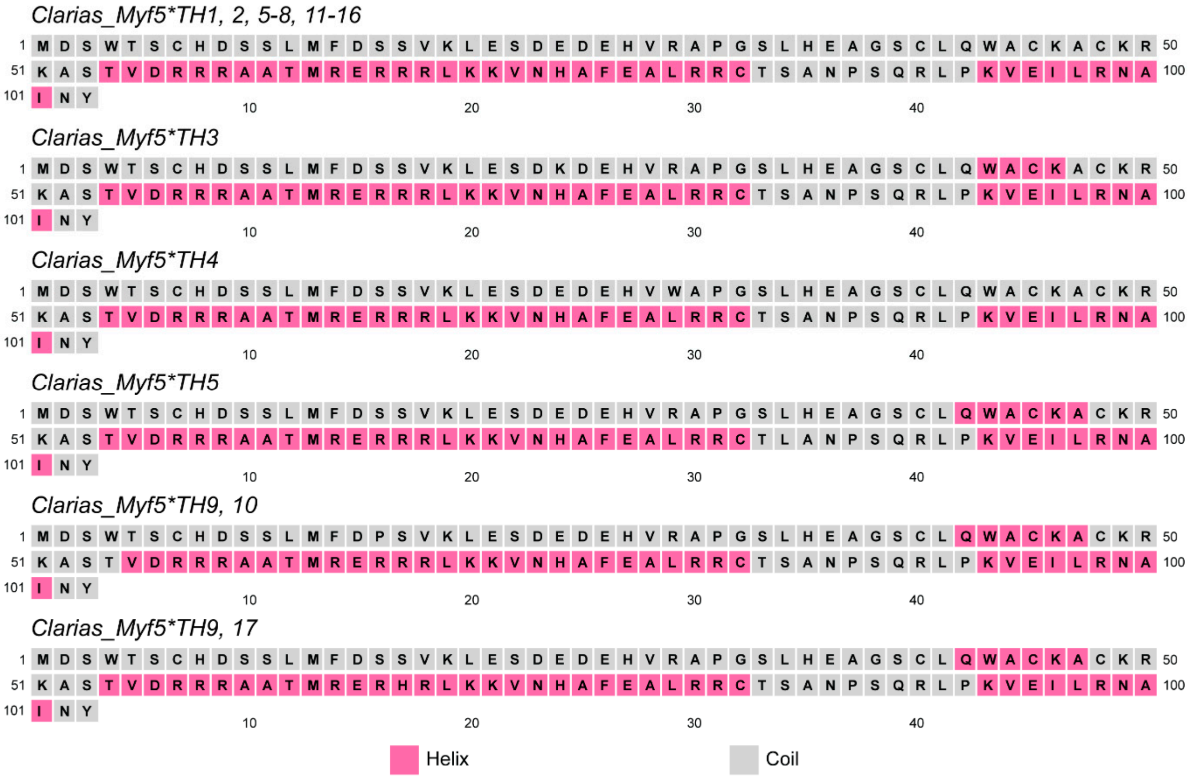

Figure S3 secondary structure production of *Myf5* protein from catfish species in Thailand

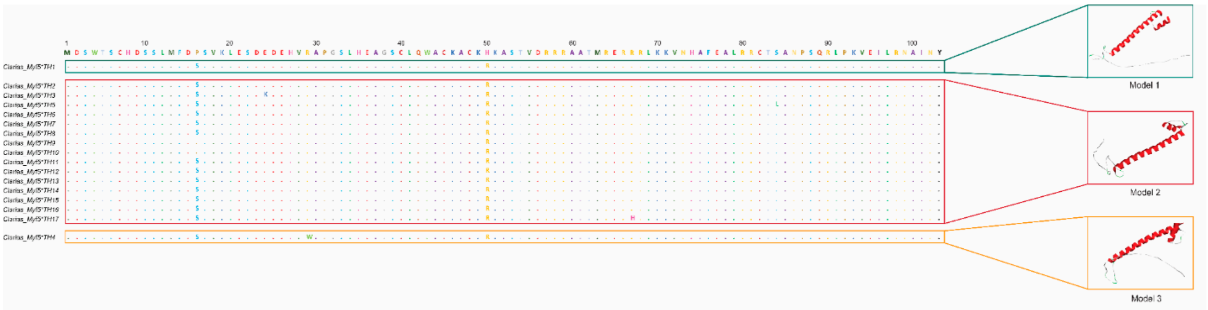

Figure S4 Alignment of amino acid sequences of *Myf5* haplotypes from exon 1 with their 3D structures of Basic helix-loop-helix(bHLH) domain retrieved from UniProt database

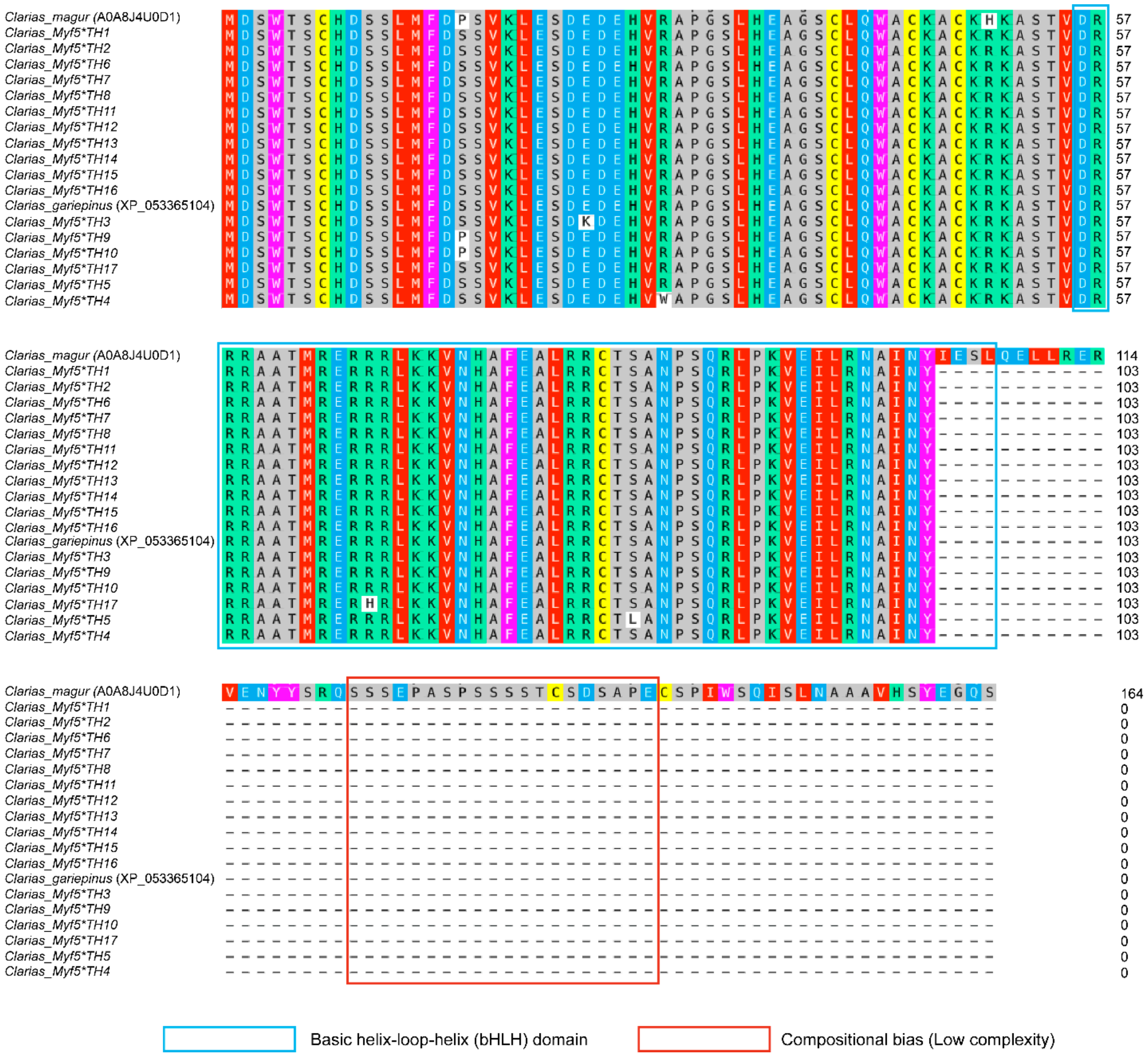

Figure S5 Alignment of amino acid sequences of exon 1 of the *Myf5* haplotypes with the reference sequences from Uniprot (A0A8J4U0D1) and *Clarias gariepinus* (XP_053365104)

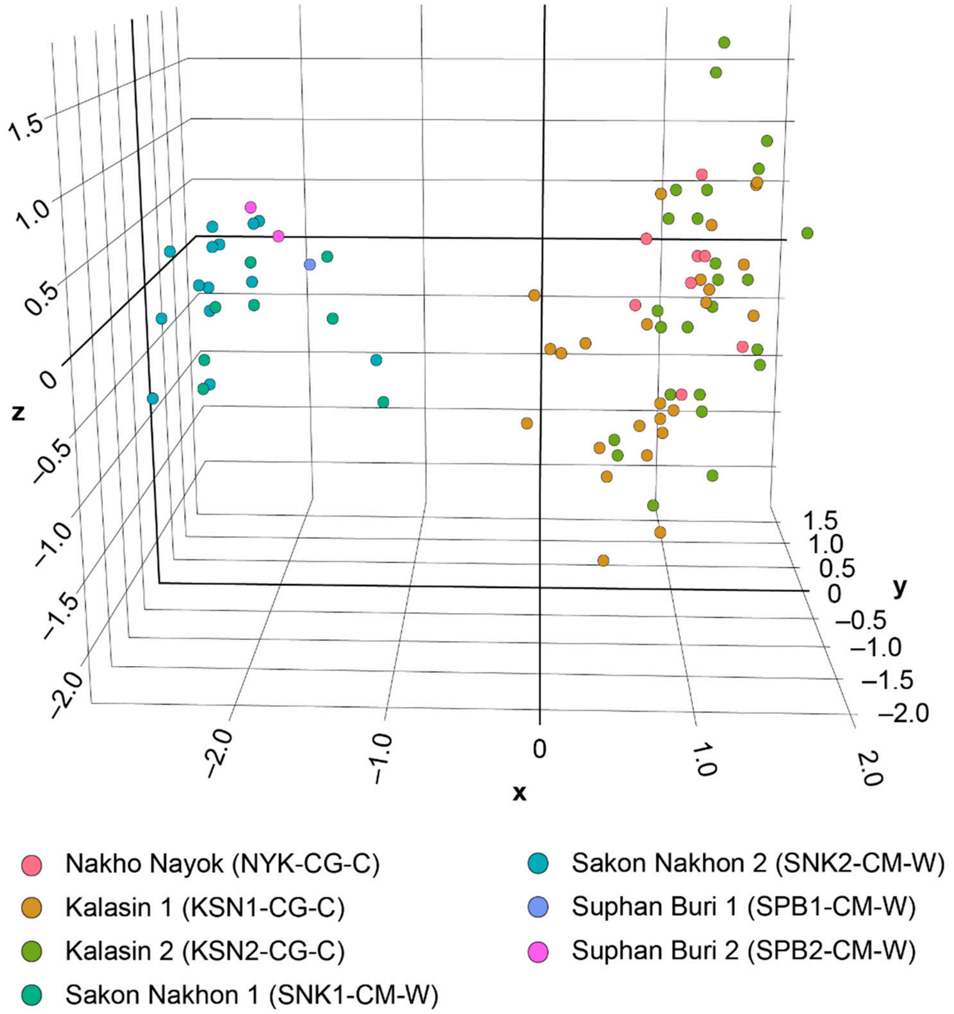

Figure S6 Discriminant analysis of principal components (DAPC) of catfish populations in Thailand. Each population is plotted by different colors.

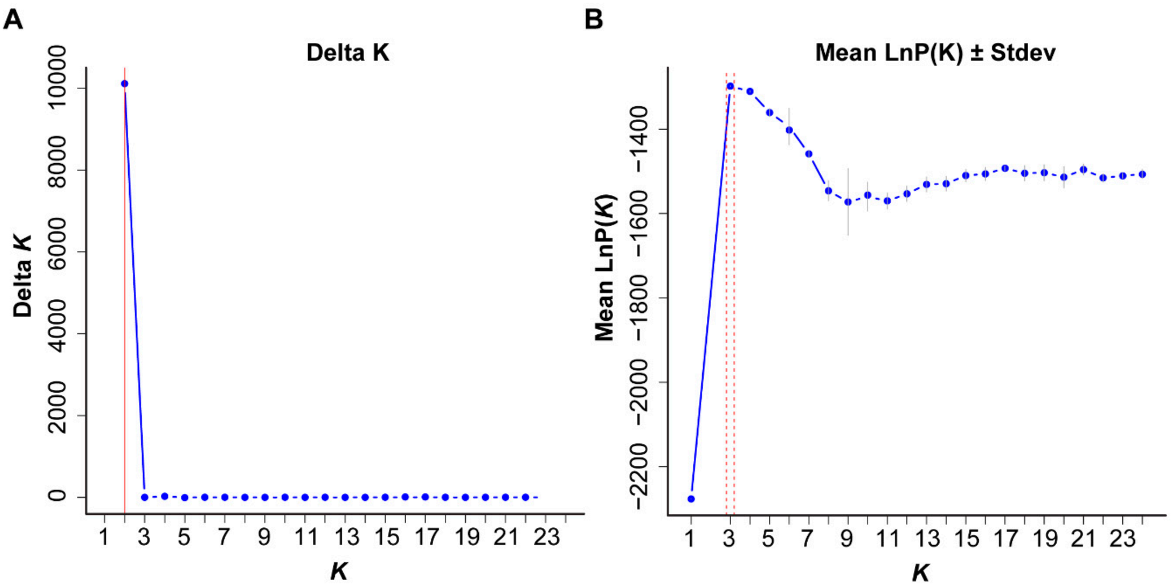

Figure S7 Different population structure patterns of catfish populations in Thailand generated by the model-based Bayesian clustering algorithms implemented in STRUCTURE based on (A) Plot of Evanno's Δ*K* and (B) Plot of Pr(X|*K*) strategy

Supplementary Table

Table S1 Summary of catfish individuals sampled in this study

No.	Abbreviation/Code	Coordinate	Locality
1	NYK-CG-C_1	14°05’58”N, 101°09’39”E	Nakhon Nayok
2	NYK-CG-C_2	14°05’58”N, 101°09’39”E	Nakhon Nayok
3	NYK-CG-C_3	14°05’58”N, 101°09’39”E	Nakhon Nayok
4	NYK-CG-C_4	14°05’58”N, 101°09’39”E	Nakhon Nayok
5	NYK-CG-C_5	14°05’58”N, 101°09’39”E	Nakhon Nayok
6	NYK-CG-C_6	14°05’58”N, 101°09’39”E	Nakhon Nayok
7	NYK-CG-C_7	14°05’58”N, 101°09’39”E	Nakhon Nayok
8	NYK-CG-C_8	14°05’58”N, 101°09’39”E	Nakhon Nayok
9	NYK-CG-C_9	14°05’58”N, 101°09’39”E	Nakhon Nayok
10	NYK-CG-C_10	14°05’58”N, 101°09’39”E	Nakhon Nayok
11	NYK-CG-C_11	14°05’58”N, 101°09’39”E	Nakhon Nayok
12	NYK-CG-C_12	14°05’58”N, 101°09’39”E	Nakhon Nayok
13	NYK-CG-C_13	14°05’58”N, 101°09’39”E	Nakhon Nayok
14	NYK-CG-C_14	14°05’58”N, 101°09’39”E	Nakhon Nayok
15	NYK-CG-C_15	14°05’58”N, 101°09’39”E	Nakhon Nayok
16	NYK-CG-C_16	14°05’58”N, 101°09’39”E	Nakhon Nayok
17	NYK-CG-C_17	14°05’58”N, 101°09’39”E	Nakhon Nayok
18	NYK-CG-C_18	14°05’58”N, 101°09’39”E	Nakhon Nayok
19	NYK-CG-C_19	14°05’58”N, 101°09’39”E	Nakhon Nayok
20	NYK-CG-C_20	14°05’58”N, 101°09’39”E	Nakhon Nayok
21	NYK-CG-C_21	14°05’58”N, 101°09’39”E	Nakhon Nayok
22	NYK-CG-C_22	14°05’58”N, 101°09’39”E	Nakhon Nayok
23	NYK-CG-C_23	14°05’58”N, 101°09’39”E	Nakhon Nayok
24	NYK-CG-C_24	14°05’58”N, 101°09’39”E	Nakhon Nayok
25	NYK-CG-C_25	14°05’58”N, 101°09’39”E	Nakhon Nayok
26	NYK-CG-C_26	14°05’58”N, 101°09’39”E	Nakhon Nayok
27	KSN1-CG-C_1	16°39’29"N, 103°29’10"E	Kalasin
28	KSN1-CG-C_2	16°39’29"N, 103°29’10"E	Kalasin
29	KSN1-CG-C_3	16°39’29"N, 103°29’10"E	Kalasin
30	KSN1-CG-C_4	16°39’29"N, 103°29’10"E	Kalasin
31	KSN1-CG-C_5	16°39’29"N, 103°29’10"E	Kalasin
32	KSN1-CG-C_6	16°39’29"N, 103°29’10"E	Kalasin
33	KSN1-CG-C_7	16°39’29"N, 103°29’10"E	Kalasin
34	KSN1-CG-C_8	16°39’29"N, 103°29’10"E	Kalasin
35	KSN1-CG-C_9	16°39’29"N, 103°29’10"E	Kalasin
36	KSN1-CG-C_10	16°39’29"N, 103°29’10"E	Kalasin
37	KSN1-CG-C_11	16°39’29"N, 103°29’10"E	Kalasin
38	KSN1-CG-C_12	16°39’29"N, 103°29’10"E	Kalasin
39	KSN1-CG-C_13	16°39’29"N, 103°29’10"E	Kalasin
40	KSN1-CG-C_14	16°39’29"N, 103°29’10"E	Kalasin
41	KSN1-CG-C_15	16°39’29"N, 103°29’10"E	Kalasin
42	KSN1-CG-C_16	16°39’29"N, 103°29’10"E	Kalasin
43	KSN1-CG-C_17	16°39’29"N, 103°29’10"E	Kalasin
44	KSN1-CG-C_18	16°39’29"N, 103°29’10"E	Kalasin

No.	Abbreviation/Code	Coordinate	Locality
45	KSN1-CG-C_19	16˚39’29"N, 103˚29’10"E	Kalasin
46	KSN1-CG-C_20	16˚39’29"N, 103˚29’10"E	Kalasin
47	KSN1-CG-C_21	16˚39’29"N, 103˚29’10"E	Kalasin
48	KSN1-CG-C_22	16˚39’29"N, 103˚29’10"E	Kalasin
49	KSN1-CG-C_23	16˚39’29"N, 103˚29’10"E	Kalasin
50	KSN1-CG-C_24	16˚39’29"N, 103˚29’10"E	Kalasin
51	KSN1-CG-C_25	16˚39’29"N, 103˚29’10"E	Kalasin
52	KSN1-CG-C_26	16˚39’29"N, 103˚29’10"E	Kalasin
53	KSN1-CG-C_27	16˚39’29"N, 103˚29’10"E	Kalasin
54	KSN1-CG-C_28	16˚39’29"N, 103˚29’10"E	Kalasin
55	KSN1-CG-C_29	16˚39’29"N, 103˚29’10"E	Kalasin
56	KSN1-CG-C_30	16˚39’29"N, 103˚29’10"E	Kalasin
57	KSN1-CG-C_31	16˚39’29"N, 103˚29’10"E	Kalasin
58	KSN1-CG-C_32	16˚39’29"N, 103˚29’10"E	Kalasin
59	KSN1-CG-C_33	16˚39’29"N, 103˚29’10"E	Kalasin
60	KSN1-CG-C_34	16˚39’29"N, 103˚29’10"E	Kalasin
61	KSN1-CG-C_35	16˚39’29"N, 103˚29’10"E	Kalasin
62	KSN1-CG-C_36	16˚39’29"N, 103˚29’10"E	Kalasin
63	KSN1-CG-C_37	16˚39’29"N, 103˚29’10"E	Kalasin
64	KSN1-CG-C_38	16˚39’29"N, 103˚29’10"E	Kalasin
65	KSN1-CG-C_39	16˚39’29"N, 103˚29’10"E	Kalasin
66	KSN1-CG-C_40	16˚39’29"N, 103˚29’10"E	Kalasin
67	KSN1-CG-C_41	16˚39’29"N, 103˚29’10"E	Kalasin
68	KSN1-CG-C_42	16˚39’29"N, 103˚29’10"E	Kalasin
69	KSN1-CG-C_43	16˚39’29"N, 103˚29’10"E	Kalasin
70	KSN1-CG-C_44	16˚39’29"N, 103˚29’10"E	Kalasin
71	KSN1-CG-C_45	16˚39’29"N, 103˚29’10"E	Kalasin
72	KSN1-CG-C_46	16˚39’29"N, 103˚29’10"E	Kalasin
73	KSN1-CG-C_47	16˚39’29"N, 103˚29’10"E	Kalasin
74	KSN1-CG-C_48	16˚39’29"N, 103˚29’10"E	Kalasin
75	KSN1-CG-C_49	16˚39’29"N, 103˚29’10"E	Kalasin
76	KSN1-CG-C_50	16˚39’29"N, 103˚29’10"E	Kalasin
77	KSN1-CG-C_51	16˚39’29"N, 103˚29’10"E	Kalasin
78	KSN1-CG-C_52	16˚39’29"N, 103˚29’10"E	Kalasin
79	KSN1-CG-C_53	16˚39’29"N, 103˚29’10"E	Kalasin
80	KSN1-CG-C_54	16˚39’29"N, 103˚29’10"E	Kalasin
81	KSN1-CG-C_55	16˚39’29"N, 103˚29’10"E	Kalasin
82	KSN1-CG-C_56	16˚39’29"N, 103˚29’10"E	Kalasin
83	KSN1-CG-C_57	16˚39’29"N, 103˚29’10"E	Kalasin
84	KSN1-CG-C_58	16˚39’29"N, 103˚29’10"E	Kalasin
85	KSN1-CG-C_59	16˚39’29"N, 103˚29’10"E	Kalasin
86	KSN1-CG-C_60	16˚39’29"N, 103˚29’10"E	Kalasin
87	KSN1-CG-C_61	16˚39’29"N, 103˚29’10"E	Kalasin
88	KSN1-CG-C_62	16˚39’29"N, 103˚29’10"E	Kalasin
89	KSN1-CG-C_63	16˚39’29"N, 103˚29’10"E	Kalasin
90	KSN1-CG-C_64	16˚39’29"N, 103˚29’10"E	Kalasin
91	KSN1-CG-C_65	16˚39’29"N, 103˚29’10"E	Kalasin

No.	Abbreviation/Code	Coordinate	Locality
92	KSN1-CG-C_66	16˚39’29"N, 103˚29’10"E	Kalasin
93	KSN1-CG-C_67	16˚39’29"N, 103˚29’10"E	Kalasin
94	KSN1-CG-C_68	16˚39’29"N, 103˚29’10"E	Kalasin
95	KSN1-CG-C_69	16˚39’29"N, 103˚29’10"E	Kalasin
96	KSN1-CG-C_70	16˚39’29"N, 103˚29’10"E	Kalasin
97	KSN2-CG-C_1	16˚39’29"N, 103˚29’10"E	Kalasin
98	KSN2-CG-C_2	16˚39’29"N, 103˚29’10"E	Kalasin
99	KSN2-CG-C_3	16˚39’29"N, 103˚29’10"E	Kalasin
100	KSN2-CG-C_4	16˚39’29"N, 103˚29’10"E	Kalasin
101	KSN2-CG-C_5	16˚39’29"N, 103˚29’10"E	Kalasin
102	KSN2-CG-C_6	16˚39’29"N, 103˚29’10"E	Kalasin
103	KSN2-CG-C_7	16˚39’29"N, 103˚29’10"E	Kalasin
104	KSN2-CG-C_8	16˚39’29"N, 103˚29’10"E	Kalasin
105	KSN2-CG-C_9	16˚39’29"N, 103˚29’10"E	Kalasin
106	KSN2-CG-C_10	16˚39’29"N, 103˚29’10"E	Kalasin
107	KSN2-CG-C_11	16˚39’29"N, 103˚29’10"E	Kalasin
108	KSN2-CG-C_12	16˚39’29"N, 103˚29’10"E	Kalasin
109	KSN2-CG-C_13	16˚39’29"N, 103˚29’10"E	Kalasin
110	KSN2-CG-C_14	16˚39’29"N, 103˚29’10"E	Kalasin
111	KSN2-CG-C_15	16˚39’29"N, 103˚29’10"E	Kalasin
112	KSN2-CG-C_16	16˚39’29"N, 103˚29’10"E	Kalasin
113	KSN2-CG-C_17	16˚39’29"N, 103˚29’10"E	Kalasin
114	KSN2-CG-C_18	16˚39’29"N, 103˚29’10"E	Kalasin
115	KSN2-CG-C_19	16˚39’29"N, 103˚29’10"E	Kalasin
116	KSN2-CG-C_20	16˚39’29"N, 103˚29’10"E	Kalasin
117	KSN2-CG-C_21	16˚39’29"N, 103˚29’10"E	Kalasin
118	KSN2-CG-C_22	16˚39’29"N, 103˚29’10"E	Kalasin
119	KSN2-CG-C_23	16˚39’29"N, 103˚29’10"E	Kalasin
120	KSN2-CG-C_24	16˚39’29"N, 103˚29’10"E	Kalasin
121	KSN2-CG-C_25	16˚39’29"N, 103˚29’10"E	Kalasin
122	KSN2-CG-C_26	16˚39’29"N, 103˚29’10"E	Kalasin
123	KSN2-CG-C_27	16˚39’29"N, 103˚29’10"E	Kalasin
124	KSN2-CG-C_28	16˚39’29"N, 103˚29’10"E	Kalasin
125	KSN2-CG-C_29	16˚39’29"N, 103˚29’10"E	Kalasin
126	KSN2-CG-C_30	16˚39’29"N, 103˚29’10"E	Kalasin
127	KSN2-CG-C_31	16˚39’29"N, 103˚29’10"E	Kalasin
128	KSN2-CG-C_32	16˚39’29"N, 103˚29’10"E	Kalasin
129	KSN2-CG-C_33	16˚39’29"N, 103˚29’10"E	Kalasin
130	KSN2-CG-C_34	16˚39’29"N, 103˚29’10"E	Kalasin
131	KSN2-CG-C_35	16˚39’29"N, 103˚29’10"E	Kalasin
132	KSN2-CG-C_36	16˚39’29"N, 103˚29’10"E	Kalasin
133	KSN2-CG-C_37	16˚39’29"N, 103˚29’10"E	Kalasin
134	KSN2-CG-C_38	16˚39’29"N, 103˚29’10"E	Kalasin
135	KSN2-CG-C_39	16˚39’29"N, 103˚29’10"E	Kalasin
136	KSN2-CG-C_40	16˚39’29"N, 103˚29’10"E	Kalasin
137	KSN2-CG-C_41	16˚39’29"N, 103˚29’10"E	Kalasin
138	KSN2-CG-C_42	16˚39’29"N, 103˚29’10"E	Kalasin

No.	Abbreviation/Code	Coordinate	Locality
139	KSN2-CG-C_43	16°39’29"N, 103°29’10"E	Kalasin
140	KSN2-CG-C_44	16°39’29"N, 103°29’10"E	Kalasin
141	KSN2-CG-C_45	16°39’29"N, 103°29’10"E	Kalasin
142	KSN2-CG-C_46	16°39’29"N, 103°29’10"E	Kalasin
143	KSN2-CG-C_47	16°39’29"N, 103°29’10"E	Kalasin
144	KSN2-CG-C_48	16°39’29"N, 103°29’10"E	Kalasin
145	KSN2-CG-C_49	16°39’29"N, 103°29’10"E	Kalasin
146	KSN2-CG-C_50	16°39’29"N, 103°29’10"E	Kalasin
147	KSN2-CG-C_51	16°39’29"N, 103°29’10"E	Kalasin
148	KSN2-CG-C_52	16°39’29"N, 103°29’10"E	Kalasin
149	KSN2-CG-C_53	16°39’29"N, 103°29’10"E	Kalasin
150	KSN2-CG-C_54	16°39’29"N, 103°29’10"E	Kalasin
151	KSN2-CG-C_55	16°39’29"N, 103°29’10"E	Kalasin
152	KSN2-CG-C_56	16°39’29"N, 103°29’10"E	Kalasin
153	KSN2-CG-C_57	16°39’29"N, 103°29’10"E	Kalasin
154	KSN2-CG-C_58	16°39’29"N, 103°29’10"E	Kalasin
155	KSN2-CG-C_59	16°39’29"N, 103°29’10"E	Kalasin
156	KSN2-CG-C_60	16°39’29"N, 103°29’10"E	Kalasin
157	KSN2-CG-C_61	16°39’29"N, 103°29’10"E	Kalasin
158	KSN2-CG-C_62	16°39’29"N, 103°29’10"E	Kalasin
159	KSN2-CG-C_63	16°39’29"N, 103°29’10"E	Kalasin
160	KSN2-CG-C_64	16°39’29"N, 103°29’10"E	Kalasin
161	KSN2-CG-C_65	16°39’29"N, 103°29’10"E	Kalasin
162	KSN2-CG-C_66	16°39’29"N, 103°29’10"E	Kalasin
163	KSN2-CG-C_67	16°39’29"N, 103°29’10"E	Kalasin
164	KSN2-CG-C_68	16°39’29"N, 103°29’10"E	Kalasin
165	KSN2-CG-C_69	16°39’29"N, 103°29’10"E	Kalasin
166	KSN2-CG-C_70	16°39’29"N, 103°29’10"E	Kalasin
167	KSN2-CG-C_71	16°39’29"N, 103°29’10"E	Kalasin
168	KSN2-CG-C_72	16°39’29"N, 103°29’10"E	Kalasin
169	KSN2-CG-C_73	16°39’29"N, 103°29’10"E	Kalasin
170	KSN2-CG-C_74	16°39’29"N, 103°29’10"E	Kalasin
171	KSN2-CG-C_75	16°39’29"N, 103°29’10"E	Kalasin
172	KSN2-CG-C_76	16°39’29"N, 103°29’10"E	Kalasin
173	KSN2-CG-C_77	16°39’29"N, 103°29’10"E	Kalasin
174	KSN2-CG-C_78	16°39’29"N, 103°29’10"E	Kalasin
175	KSN2-CG-C_79	16°39’29"N, 103°29’10"E	Kalasin
176	KSN2-CG-C_80	16°39’29"N, 103°29’10"E	Kalasin
177	KSN2-CG-C_81	16°39’29"N, 103°29’10"E	Kalasin
178	KSN2-CG-C_82	16°39’29"N, 103°29’10"E	Kalasin
179	KSN2-CG-C_83	16°39’29"N, 103°29’10"E	Kalasin
180	KSN2-CG-C_84	16°39’29"N, 103°29’10"E	Kalasin
181	KSN2-CG-C_85	16°39’29"N, 103°29’10"E	Kalasin
182	KSN2-CG-C_86	16°39’29"N, 103°29’10"E	Kalasin
183	KSN2-CG-C_87	16°39’29"N, 103°29’10"E	Kalasin
184	KSN2-CG-C_88	16°39’29"N, 103°29’10"E	Kalasin
185	KSN2-CG-C_89	16°39’29"N, 103°29’10"E	Kalasin

No.	Abbreviation/Code	Coordinate	Locality
186	KSN2-CG-C_90	16˚39’29"N, 103˚29’10"E	Kalasin
187	KSN2-CG-C_91	16˚39’29"N, 103˚29’10"E	Kalasin
188	KSN2-CG-C_92	16˚39’29"N, 103˚29’10"E	Kalasin
189	KSN2-CG-C_93	16˚39’29"N, 103˚29’10"E	Kalasin
190	KSN2-CG-C_94	16˚39’29"N, 103˚29’10"E	Kalasin
191	KSN2-CG-C_95	16˚39’29"N, 103˚29’10"E	Kalasin
192	SBR-CM-C_1	14˚58’13"N, 100˚18’41"E	Sing Buri
193	SBR-CM-C_2	14˚58’13"N, 100˚18’41"E	Sing Buri
194	SNK1-CM-W_1	17°21’14"N, 104°19’16"E	Sakon Nakhon
195	SNK1-CM-W_2	17°21’14"N, 104°19’16"E	Sakon Nakhon
196	SNK1-CM-W_3	17°21’14"N, 104°19’16"E	Sakon Nakhon
197	SNK1-CM-W_4	17°21’14"N, 104°19’16"E	Sakon Nakhon
198	SNK1-CM-W_5	17°21’14"N, 104°19’16"E	Sakon Nakhon
199	SNK1-CM-W_6	17°21’14"N, 104°19’16"E	Sakon Nakhon
200	SNK1-CM-W_7	17°21’14"N, 104°19’16"E	Sakon Nakhon
201	SNK1-CM-W_8	17°21’14"N, 104°19’16"E	Sakon Nakhon
202	SNK1-CM-W_9	17°21’14"N, 104°19’16"E	Sakon Nakhon
203	SNK1-CM-W_10	17°21’14"N, 104°19’16"E	Sakon Nakhon
204	SNK1-CM-W_11	17°21’14"N, 104°19’16"E	Sakon Nakhon
205	SNK1-CM-W_12	17°21’14"N, 104°19’16"E	Sakon Nakhon
206	SNK1-CM-W_13	17°21’14"N, 104°19’16"E	Sakon Nakhon
207	SNK1-CM-W_14	17°21’14"N, 104°19’16"E	Sakon Nakhon
208	SNK1-CM-W_15	17°21’14"N, 104°19’16"E	Sakon Nakhon
209	SNK1-CM-W_16	17°21’14"N, 104°19’16"E	Sakon Nakhon
210	SNK1-CM-W_17	17°21’14"N, 104°19’16"E	Sakon Nakhon
211	SNK1-CM-W_18	17°21’14"N, 104°19’16"E	Sakon Nakhon
212	SNK1-CM-W_19	17°21’14"N, 104°19’16"E	Sakon Nakhon
213	SNK1-CM-W_20	17°21’14"N, 104°19’16"E	Sakon Nakhon
214	SNK1-CM-W_21	17°21’14"N, 104°19’16"E	Sakon Nakhon
215	SNK1-CM-W_22	17°21’14"N, 104°19’16"E	Sakon Nakhon
216	SNK1-CM-W_23	17°21’14"N, 104°19’16"E	Sakon Nakhon
217	SNK1-CM-W_24	17°21’14"N, 104°19’16"E	Sakon Nakhon
218	SNK1-CM-W_25	17°21’14"N, 104°19’16"E	Sakon Nakhon
219	SNK1-CM-W_26	17°21’14"N, 104°19’16"E	Sakon Nakhon
220	SNK1-CM-W_27	17°21’14"N, 104°19’16"E	Sakon Nakhon
221	SNK1-CM-W_28	17°21’14"N, 104°19’16"E	Sakon Nakhon
222	SNK1-CM-W_29	17°21’14"N, 104°19’16"E	Sakon Nakhon
223	SNK1-CM-W_30	17°21’14"N, 104°19’16"E	Sakon Nakhon
224	SNK2-CM-W_1	17°14’05"N, 104°12’28"E	Sakon Nakhon
225	SNK2-CM-W_2	17°14’05"N, 104°12’28"E	Sakon Nakhon
226	SNK2-CM-W_3	17°14’05"N, 104°12’28"E	Sakon Nakhon
227	SNK2-CM-W_4	17°14’05"N, 104°12’28"E	Sakon Nakhon
228	SNK2-CM-W_5	17°14’05"N, 104°12’28"E	Sakon Nakhon
229	SNK2-CM-W_6	17°14’05"N, 104°12’28"E	Sakon Nakhon
230	SNK2-CM-W_7	17°14’05"N, 104°12’28"E	Sakon Nakhon
231	SNK2-CM-W_8	17°14’05"N, 104°12’28"E	Sakon Nakhon
232	SNK2-CM-W_9	17°14’05"N, 104°12’28"E	Sakon Nakhon

No.	Abbreviation/Code	Coordinate	Locality
233	SNK2-CM-W_10	17°14’05"N, 104°12’28"E	Sakon Nakhon
234	SNK2-CM-W_11	17°14’05"N, 104°12’28"E	Sakon Nakhon
235	SNK2-CM-W_12	17°14’05"N, 104°12’28"E	Sakon Nakhon
236	SNK2-CM-W_13	17°14’05"N, 104°12’28"E	Sakon Nakhon
237	SNK2-CM-W_14	17°14’05"N, 104°12’28"E	Sakon Nakhon
238	SNK2-CM-W_15	17°14’05"N, 104°12’28"E	Sakon Nakhon
239	SNK2-CM-W_16	17°14’05"N, 104°12’28"E	Sakon Nakhon
240	SNK2-CM-W_17	17°14’05"N, 104°12’28"E	Sakon Nakhon
241	SNK2-CM-W_18	17°14’05"N, 104°12’28"E	Sakon Nakhon
242	SNK2-CM-W_19	17°14’05"N, 104°12’28"E	Sakon Nakhon
243	SNK2-CM-W_20	17°14’05"N, 104°12’28"E	Sakon Nakhon
244	SNK2-CM-W_21	17°14’05"N, 104°12’28"E	Sakon Nakhon
245	SNK2-CM-W_22	17°14’05"N, 104°12’28"E	Sakon Nakhon
246	SNK2-CM-W_23	17°14’05"N, 104°12’28"E	Sakon Nakhon
247	SNK2-CM-W_24	17°14’05"N, 104°12’28"E	Sakon Nakhon
248	SNK2-CM-W_25	17°14’05"N, 104°12’28"E	Sakon Nakhon
249	SNK2-CM-W_26	17°14’05"N, 104°12’28"E	Sakon Nakhon
250	SNK2-CM-W_27	17°14’05"N, 104°12’28"E	Sakon Nakhon
251	SNK2-CM-W_28	17°14’05"N, 104°12’28"E	Sakon Nakhon
252	SNK2-CM-W_29	17°14’05"N, 104°12’28"E	Sakon Nakhon
253	SNK2-CM-W_30	17°14’05"N, 104°12’28"E	Sakon Nakhon
254	SNK2-CM-W_31	17°14’05"N, 104°12’28"E	Sakon Nakhon
255	SNK2-CM-W_32	17°14’05"N, 104°12’28"E	Sakon Nakhon
256	SNK2-CM-W_33	17°14’05"N, 104°12’28"E	Sakon Nakhon
257	SNK2-CM-W_34	17°14’05"N, 104°12’28"E	Sakon Nakhon
258	SNK2-CM-W_35	17°14’05"N, 104°12’28"E	Sakon Nakhon
259	SNK2-CM-W_36	17°14’05"N, 104°12’28"E	Sakon Nakhon
260	SNK2-CM-W_37	17°14’05"N, 104°12’28"E	Sakon Nakhon
261	SNK2-CM-W_38	17°14’05"N, 104°12’28"E	Sakon Nakhon
262	SNK2-CM-W_39	17°14’05"N, 104°12’28"E	Sakon Nakhon
263	SNK2-CM-W_40	17°14’05"N, 104°12’28"E	Sakon Nakhon
264	SNK2-CM-W_41	17°14’05"N, 104°12’28"E	Sakon Nakhon
265	SNK2-CM-W_42	17°14’05"N, 104°12’28"E	Sakon Nakhon
266	SNK2-CM-W_43	17°14’05"N, 104°12’28"E	Sakon Nakhon
267	SNK2-CM-W_44	17°14’05"N, 104°12’28"E	Sakon Nakhon
268	SNK2-CM-W_45	17°14’05"N, 104°12’28"E	Sakon Nakhon
269	SNK2-CM-W_46	17°14’05"N, 104°12’28"E	Sakon Nakhon
270	SNK2-CM-W_47	17°14’05"N, 104°12’28"E	Sakon Nakhon
271	SNK2-CM-W_48	17°14’05"N, 104°12’28"E	Sakon Nakhon
272	SNK2-CM-W_49	17°14’05"N, 104°12’28"E	Sakon Nakhon
273	SNK2-CM-W_50	17°14’05"N, 104°12’28"E	Sakon Nakhon
274	SNK2-CM-W_51	17°14’05"N, 104°12’28"E	Sakon Nakhon
275	SNK2-CM-W_52	17°14’05"N, 104°12’28"E	Sakon Nakhon
276	SNK2-CM-W_53	17°14’05"N, 104°12’28"E	Sakon Nakhon
277	SNK2-CM-W_54	17°14’05"N, 104°12’28"E	Sakon Nakhon
278	SNK2-CM-W_55	17°14’05"N, 104°12’28"E	Sakon Nakhon
279	SNK2-CM-W_56	17°14’05"N, 104°12’28"E	Sakon Nakhon

No.	Abbreviation/Code	Coordinate	Locality
280	SNK2-CM-W_57	17°14’05"N, 104°12’28"E	Sakon Nakhon
281	SNK2-CM-W_58	17°14’05"N, 104°12’28"E	Sakon Nakhon
282	SNK2-CM-W_59	17°14’05"N, 104°12’28"E	Sakon Nakhon
283	SNK2-CM-W_60	17°14’05"N, 104°12’28"E	Sakon Nakhon
284	SNK2-CM-W_61	17°14’05"N, 104°12’28"E	Sakon Nakhon
285	SNK2-CM-W_62	17°14’05"N, 104°12’28"E	Sakon Nakhon
286	SNK2-CM-W_63	17°14’05"N, 104°12’28"E	Sakon Nakhon
287	SNK2-CM-W_64	17°14’05"N, 104°12’28"E	Sakon Nakhon
288	SNK2-CM-W_65	17°14’05"N, 104°12’28"E	Sakon Nakhon
289	SNK2-CM-W_66	17°14’05"N, 104°12’28"E	Sakon Nakhon
290	SNK2-CM-W_67	17°14’05"N, 104°12’28"E	Sakon Nakhon
291	SNK2-CM-W_68	17°14’05"N, 104°12’28"E	Sakon Nakhon
292	SNK2-CM-W_69	17°14’05"N, 104°12’28"E	Sakon Nakhon
293	SNK2-CM-W_70	17°14’05"N, 104°12’28"E	Sakon Nakhon
294	SNK2-CM-W_71	17°14’05"N, 104°12’28"E	Sakon Nakhon
295	SNK2-CM-W_72	17°14’05"N, 104°12’28"E	Sakon Nakhon
296	SNK2-CM-W_73	17°14’05"N, 104°12’28"E	Sakon Nakhon
297	SNK2-CM-W_74	17°14’05"N, 104°12’28"E	Sakon Nakhon
298	SNK2-CM-W_75	17°14’05"N, 104°12’28"E	Sakon Nakhon
299	SNK2-CM-W_76	17°14’05"N, 104°12’28"E	Sakon Nakhon
300	SNK2-CM-W_77	17°14’05"N, 104°12’28"E	Sakon Nakhon
301	SNK2-CM-W_78	17°14’05"N, 104°12’28"E	Sakon Nakhon
302	SNK2-CM-W_79	17°14’05"N, 104°12’28"E	Sakon Nakhon
303	SNK2-CM-W_80	17°14’05"N, 104°12’28"E	Sakon Nakhon
304	SNK2-CM-W_81	17°14’05"N, 104°12’28"E	Sakon Nakhon
305	SNK2-CM-W_82	17°14’05"N, 104°12’28"E	Sakon Nakhon
306	SNK2-CM-W_83	17°14’05"N, 104°12’28"E	Sakon Nakhon
307	SNK2-CM-W_84	17°14’05"N, 104°12’28"E	Sakon Nakhon
308	SNK2-CM-W_85	17°14’05"N, 104°12’28"E	Sakon Nakhon
309	SNK2-CM-W_86	17°14’05"N, 104°12’28"E	Sakon Nakhon
310	SNK2-CM-W_87	17°14’05"N, 104°12’28"E	Sakon Nakhon
311	SNK2-CM-W_88	17°14’05"N, 104°12’28"E	Sakon Nakhon
312	SNK2-CM-W_89	17°14’05"N, 104°12’28"E	Sakon Nakhon
313	SNK2-CM-W_90	17°14’05"N, 104°12’28"E	Sakon Nakhon
314	SNK2-CM-W_91	17°14’05"N, 104°12’28"E	Sakon Nakhon
315	SNK2-CM-W_92	17°14’05"N, 104°12’28"E	Sakon Nakhon
316	SNK2-CM-W_93	17°14’05"N, 104°12’28"E	Sakon Nakhon
317	SNK2-CM-W_94	17°14’05"N, 104°12’28"E	Sakon Nakhon
318	SNK2-CM-W_95	17°14’05"N, 104°12’28"E	Sakon Nakhon
319	SNK2-CM-W_96	17°14’05"N, 104°12’28"E	Sakon Nakhon
320	SNK2-CM-W_97	17°14’05"N, 104°12’28"E	Sakon Nakhon
321	SNK2-CM-W_98	17°14’05"N, 104°12’28"E	Sakon Nakhon
322	SNK2-CM-W_99	17°14’05"N, 104°12’28"E	Sakon Nakhon
323	SNK2-CM-W_100	17°14’05"N, 104°12’28"E	Sakon Nakhon
324	SNK2-CM-W_101	17°14’05"N, 104°12’28"E	Sakon Nakhon
325	SNK2-CM-W_102	17°14’05"N, 104°12’28"E	Sakon Nakhon
326	SNK2-CM-W_103	17°14’05"N, 104°12’28"E	Sakon Nakhon

No.	Abbreviation/Code	Coordinate	Locality
327	SNK2-CM-W_104	17°14’05"N, 104°12’28"E	Sakon Nakhon
328	SNK2-CM-W_105	17°14’05"N, 104°12’28"E	Sakon Nakhon
329	SNK2-CM-W_106	17°14’05"N, 104°12’28"E	Sakon Nakhon
330	SNK2-CM-W_107	17°14’05"N, 104°12’28"E	Sakon Nakhon
331	SNK2-CM-W_108	17°14’05"N, 104°12’28"E	Sakon Nakhon
332	SNK2-CM-W_109	17°14’05"N, 104°12’28"E	Sakon Nakhon
333	SNK2-CM-W_110	17°14’05"N, 104°12’28"E	Sakon Nakhon
334	SNK2-CM-W_111	17°14’05"N, 104°12’28"E	Sakon Nakhon
335	SNK2-CM-W_112	17°14’05"N, 104°12’28"E	Sakon Nakhon
336	SNK2-CM-W_113	17°14’05"N, 104°12’28"E	Sakon Nakhon
337	SNK2-CM-W_114	17°14’05"N, 104°12’28"E	Sakon Nakhon
338	SNK2-CM-W_115	17°14’05"N, 104°12’28"E	Sakon Nakhon
339	SNK2-CM-W_116	17°14’05"N, 104°12’28"E	Sakon Nakhon
340	SNK2-CM-W_117	17°14’05"N, 104°12’28"E	Sakon Nakhon
341	SNK2-CM-W_118	17°14’05"N, 104°12’28"E	Sakon Nakhon
342	SNK2-CM-W_119	17°14’05"N, 104°12’28"E	Sakon Nakhon
343	SNK2-CM-W_120	17°14’05"N, 104°12’28"E	Sakon Nakhon
344	SNK2-CM-W_121	17°14’05"N, 104°12’28"E	Sakon Nakhon
345	SNK2-CM-W_122	17°14’05"N, 104°12’28"E	Sakon Nakhon
346	SNK2-CM-W_123	17°14’05"N, 104°12’28"E	Sakon Nakhon
347	SNK2-CM-W_124	17°14’05"N, 104°12’28"E	Sakon Nakhon
348	SNK2-CM-W_125	17°14’05"N, 104°12’28"E	Sakon Nakhon
349	SNK2-CM-W_126	17°14’05"N, 104°12’28"E	Sakon Nakhon
350	SNK2-CM-W_127	17°14’05"N, 104°12’28"E	Sakon Nakhon
351	SNK2-CM-W_128	17°14’05"N, 104°12’28"E	Sakon Nakhon
352	SNK2-CM-W_129	17°14’05"N, 104°12’28"E	Sakon Nakhon
353	SNK2-CM-W_130	17°14’05"N, 104°12’28"E	Sakon Nakhon
354	SNK2-CM-W_131	17°14’05"N, 104°12’28"E	Sakon Nakhon
355	SNK2-CM-W_132	17°14’05"N, 104°12’28"E	Sakon Nakhon
356	SNK2-CM-W_133	17°14’05"N, 104°12’28"E	Sakon Nakhon
357	SNK2-CM-W_134	17°14’05"N, 104°12’28"E	Sakon Nakhon
358	SNK2-CM-W_135	17°14’05"N, 104°12’28"E	Sakon Nakhon
359	SNK2-CM-W_136	17°14’05"N, 104°12’28"E	Sakon Nakhon
360	SNK3-CM-W_1	17°14’46"N, 104°23’82"E	Sakon Nakhon
361	SNK3-CM-W_2	17°14’46"N, 104°23’82"E	Sakon Nakhon
362	SNK3-CM-W_3	17°14’46"N, 104°23’82"E	Sakon Nakhon
363	SNK3-CM-W_4	17°14’46"N, 104°23’82"E	Sakon Nakhon
364	SNK3-CM-W_5	17°14’46"N, 104°23’82"E	Sakon Nakhon
365	SNK3-CM-W_6	17°14’46"N, 104°23’82"E	Sakon Nakhon
366	SNK3-CM-W_7	17°14’46"N, 104°23’82"E	Sakon Nakhon
367	SNK3-CM-W_8	17°14’46"N, 104°23’82"E	Sakon Nakhon
368	SNK3-CM-W_9	17°14’46"N, 104°23’82"E	Sakon Nakhon
369	SNK3-CM-W_10	17°14’46"N, 104°23’82"E	Sakon Nakhon
370	SNK3-CM-W_11	17°14’46"N, 104°23’82"E	Sakon Nakhon
371	SNK3-CM-W_12	17°14’46"N, 104°23’82"E	Sakon Nakhon
372	SNK3-CM-W_13	17°14’46"N, 104°23’82"E	Sakon Nakhon
373	SNK3-CM-W_14	17°14’46"N, 104°23’82"E	Sakon Nakhon

No.	Abbreviation/Code	Coordinate	Locality
374	SNK3-CM-W_15	17°14’46"N, 104°23’82"E	Sakon Nakhon
375	SNK3-CM-W_16	17°14’46"N, 104°23’82"E	Sakon Nakhon
376	SNK3-CM-W_17	17°14’46"N, 104°23’82"E	Sakon Nakhon
377	SNK3-CM-W_18	17°14’46"N, 104°23’82"E	Sakon Nakhon
378	SNK3-CM-W_19	17°14’46"N, 104°23’82"E	Sakon Nakhon
379	SNK3-CM-W_20	17°14’46"N, 104°23’82"E	Sakon Nakhon
380	SNK3-CM-W_21	17°14’46"N, 104°23’82"E	Sakon Nakhon
381	SNK3-CM-W_22	17°14’46"N, 104°23’82"E	Sakon Nakhon
382	SNK3-CM-W_23	17°14’46"N, 104°23’82"E	Sakon Nakhon
383	SNK3-CM-W_24	17°14’46"N, 104°23’82"E	Sakon Nakhon
384	SNK3-CM-W_25	17°14’46"N, 104°23’82"E	Sakon Nakhon
385	SNK3-CM-W_26	17°14’46"N, 104°23’82"E	Sakon Nakhon
386	SNK3-CM-W_27	17°14’46"N, 104°23’82"E	Sakon Nakhon
387	SNK3-CM-W_28	17°14’46"N, 104°23’82"E	Sakon Nakhon
388	SNK3-CM-W_29	17°14’46"N, 104°23’82"E	Sakon Nakhon
389	SNK3-CM-W_30	17°14’46"N, 104°23’82"E	Sakon Nakhon
390	SNK3-CM-W_31	17°14’46"N, 104°23’82"E	Sakon Nakhon
391	SNK3-CM-W_32	17°14’46"N, 104°23’82"E	Sakon Nakhon
392	SNK3-CM-W_33	17°14’46"N, 104°23’82"E	Sakon Nakhon
393	SNK3-CM-W_34	17°14’46"N, 104°23’82"E	Sakon Nakhon
394	SNK3-CM-W_35	17°14’46"N, 104°23’82"E	Sakon Nakhon
395	SNK3-CM-W_36	17°14’46"N, 104°23’82"E	Sakon Nakhon
396	SNK3-CM-W_37	17°14’46"N, 104°23’82"E	Sakon Nakhon
397	SNK3-CM-W_38	17°14’46"N, 104°23’82"E	Sakon Nakhon
398	SNK3-CM-W_39	17°14’46"N, 104°23’82"E	Sakon Nakhon
399	SNK3-CM-W_40	17°14’46"N, 104°23’82"E	Sakon Nakhon
400	SNK3-CM-W_41	17°14’46"N, 104°23’82"E	Sakon Nakhon
401	SNK3-CM-W_42	17°14’46"N, 104°23’82"E	Sakon Nakhon
402	SNK3-CM-W_43	17°14’46"N, 104°23’82"E	Sakon Nakhon
403	SNK3-CM-W_44	17°14’46"N, 104°23’82"E	Sakon Nakhon
404	SNK3-CM-W_45	17°14’46"N, 104°23’82"E	Sakon Nakhon
405	SNK3-CM-W_46	17°14’46"N, 104°23’82"E	Sakon Nakhon
406	SNK3-CM-W_47	17°14’46"N, 104°23’82"E	Sakon Nakhon
407	SNK3-CM-W_48	17°14’46"N, 104°23’82"E	Sakon Nakhon
408	SNK3-CM-W_49	17°14’46"N, 104°23’82"E	Sakon Nakhon
409	SNK3-CM-W_50	17°14’46"N, 104°23’82"E	Sakon Nakhon
410	SNK3-CM-W_51	17°14’46"N, 104°23’82"E	Sakon Nakhon
411	SNK3-CM-W_52	17°14’46"N, 104°23’82"E	Sakon Nakhon
412	SNK3-CM-W_53	17°14’46"N, 104°23’82"E	Sakon Nakhon
413	SNK3-CM-W_54	17°14’46"N, 104°23’82"E	Sakon Nakhon
414	SNK3-CM-W_55	17°14’46"N, 104°23’82"E	Sakon Nakhon
415	SNK3-CM-W_56	17°14’46"N, 104°23’82"E	Sakon Nakhon
416	SNK3-CM-W_57	17°14’46"N, 104°23’82"E	Sakon Nakhon
417	SNK3-CM-W_58	17°14’46"N, 104°23’82"E	Sakon Nakhon
418	SNK3-CM-W_59	17°14’46"N, 104°23’82"E	Sakon Nakhon
419	SNK3-CM-W_60	17°14’46"N, 104°23’82"E	Sakon Nakhon
420	SNK3-CM-W_61	17°14’46"N, 104°23’82"E	Sakon Nakhon

No.	Abbreviation/Code	Coordinate	Locality
421	SNK3-CM-W_62	17°14’46"N, 104°23’82"E	Sakon Nakhon
422	SNK3-CM-W_63	17°14’46"N, 104°23’82"E	Sakon Nakhon
423	SPB1-CM-W_1	14°14’57.1"N, 100°11’13.0"E	Suphan Buri
424	SPB1-CM-W_2	14°14’57.1"N, 100°11’13.0"E	Suphan Buri
425	SPB1-CM-W_3	14°14’57.1"N, 100°11’13.0"E	Suphan Buri
426	SPB1-CM-W_4	14°14’57.1"N, 100°11’13.0"E	Suphan Buri
427	SPB1-CM-W_5	14°14’57.1"N, 100°11’13.0"E	Suphan Buri
428	SPB2-CM-W_1	14°14’40.5"N, 100°07’60.0"E	Suphan Buri
429	SPB2-CM-W_2	14°14’40.5"N, 100°07’60.0"E	Suphan Buri
430	SPB2-CM-W_3	14°14’40.5"N, 100°07’60.0"E	Suphan Buri
431	NPT-CM-W_1	13°59’17.7"N, 99°56’37.3"E	Nakhon Pathom
432	NPT-CM-W_2	13°59’17.7"N, 99°56’37.3"E	Nakhon Pathom
433	UBR-CM-C_1	15°14’25"N, 104°51’23"E	Ubon Ratchathani
434	UBR-CM-C_2	15°14’25"N, 104°51’23"E	Ubon Ratchathani
435	UBR-CM-C_3	15°14’25"N, 104°51’23"E	Ubon Ratchathani
436	UBR-CM-C_4	15°14’25"N, 104°51’23"E	Ubon Ratchathani
437	UBR-CB-C_1	15°14’25"N, 104°51’23"E	Ubon Ratchathani
438	UBR-CB-C_2	15°14’25"N, 104°51’23"E	Ubon Ratchathani
439	UBR-CB-C_3	15°14’25"N, 104°51’23"E	Ubon Ratchathani
440	CR-CM-C_1	19°52’36"N, 99°55’42"E	Chiang Rai
441	CR-CM-C_2	19°52’36"N, 99°55’42"E	Chiang Rai
442	CR-CM-C_3	19°52’36"N, 99°55’42"E	Chiang Rai
443	CR-CM-C_4	19°52’36"N, 99°55’42"E	Chiang Rai
444	CR-CM-C_5	19°52’36"N, 99°55’42"E	Chiang Rai
445	CR-CM-C_6	19°52’36"N, 99°55’42"E	Chiang Rai
446	CR-CM-C_7	19°52’36"N, 99°55’42"E	Chiang Rai
447	CR-CM-C_8	19°52’36"N, 99°55’42"E	Chiang Rai
448	CR-CM-C_9	19°52’36"N, 99°55’42"E	Chiang Rai
449	CR-CM-C_10	19°52’36"N, 99°55’42"E	Chiang Rai
450	CR-CM-C_11	19°52’36"N, 99°55’42"E	Chiang Rai
451	CR-CM-C_12	19°52’36"N, 99°55’42"E	Chiang Rai
452	CR-CM-C_13	19°52’36"N, 99°55’42"E	Chiang Rai
453	CR-CM-C_14	19°52’36"N, 99°55’42"E	Chiang Rai
454	CR-CM-C_15	19°52’36"N, 99°55’42"E	Chiang Rai
455	CR-CM-C_16	19°52’36"N, 99°55’42"E	Chiang Rai
456	CR-CM-C_17	19°52’36"N, 99°55’42"E	Chiang Rai
457	CR-CM-C_18	19°52’36"N, 99°55’42"E	Chiang Rai
458	CR-CM-C_19	19°52’36"N, 99°55’42"E	Chiang Rai
459	CR-CM-C_20	19°52’36"N, 99°55’42"E	Chiang Rai
460	CR-CM-C_21	19°52’36"N, 99°55’42"E	Chiang Rai
461	YLA-CM-C_1	6°34’42"N, 101°25’47"E	Yala
462	YLA-CM-C_2	6°34’42"N, 101°25’47"E	Yala
463	YLA-CM-C_3	6°34’42"N, 101°25’47"E	Yala
464	YLA-CM-C_4	6°34’42"N, 101°25’47"E	Yala
465	YLA-CM-C_5	6°34’42"N, 101°25’47"E	Yala
466	YLA-CM-C_6	6°34’42"N, 101°25’47"E	Yala
467	YLA-CM-C_7	6°34’42"N, 101°25’47"E	Yala

No.	Abbreviation/Code	Coordinate	Locality
468	YLA-CM-C_8	6°34’42"N, 101°25’47"E	Yala
469	YLA-CM-C_9	6°34’42"N, 101°25’47"E	Yala
470	YLA-CM-C_10	6°34’42"N, 101°25’47"E	Yala
471	YLA-CM-C_11	6°34’42"N, 101°25’47"E	Yala
472	YLA-CM-C_12	6°34’42"N, 101°25’47"E	Yala
473	YLA-CM-C_13	6°34’42"N, 101°25’47"E	Yala
474	YLA-CM-C_14	6°34’42"N, 101°25’47"E	Yala
475	YLA-CM-C_15	6°34’42"N, 101°25’47"E	Yala
476	YLA-CM-C_16	6°34’42"N, 101°25’47"E	Yala
477	YLA-CM-C_17	6°34’42"N, 101°25’47"E	Yala
478	YLA-CM-C_18	6°34’42"N, 101°25’47"E	Yala
479	YLA-CM-C_19	6°34’42"N, 101°25’47"E	Yala
480	SKW-CM-C_1	13°22’33"N, 102°07’46"E	Sa Kaeo
481	SKW-CM-C_2	13°22’33"N, 102°07’46"E	Sa Kaeo
482	SKW-CM-C_3	13°22’33"N, 102°07’46"E	Sa Kaeo
483	SKW-CM-C_4	13°22’33"N, 102°07’46"E	Sa Kaeo
484	SKW-CM-C_5	13°22’33"N, 102°07’46"E	Sa Kaeo
485	SKW-CM-C_6	13°22’33"N, 102°07’46"E	Sa Kaeo
486	SKW-CM-C_7	13°22’33"N, 102°07’46"E	Sa Kaeo
487	SKW-CM-C_8	13°22’33"N, 102°07’46"E	Sa Kaeo
488	SKW-CM-C_9	13°22’33"N, 102°07’46"E	Sa Kaeo
489	SKW-CM-C_10	13°22’33"N, 102°07’46"E	Sa Kaeo
490	SKW-CM-C_11	13°22’33"N, 102°07’46"E	Sa Kaeo
491	SKW-CM-C_12	13°22’33"N, 102°07’46"E	Sa Kaeo
492	SKW-CM-C_13	13°22’33"N, 102°07’46"E	Sa Kaeo
493	SKW-CM-C_14	13°22’33"N, 102°07’46"E	Sa Kaeo
494	SKW-CM-C_15	13°22’33"N, 102°07’46"E	Sa Kaeo
495	SKW-CM-C_16	13°22’33"N, 102°07’46"E	Sa Kaeo
496	SKW-CM-C_17	13°22’33"N, 102°07’46"E	Sa Kaeo
497	SKW-CM-C_18	13°22’33"N, 102°07’46"E	Sa Kaeo
498	PLG-CM-C_1	8°04’05.4"N, 98°59’29.1"E	Phang Nga
499	PLG-CM-C_2	8°04’05.4"N, 98°59’29.1"E	Phang Nga
500	PLG-CM-C_3	8°04’05.4"N, 98°59’29.1"E	Phang Nga
501	PTLH-HB-C_1	7°49’19"N, 100°06’39"E	Phatthalung
502	PTLH-HB-C_2	7°49’19"N, 100°06’39"E	Phatthalung
503	PTLH-HB-C_3	7°49’19"N, 100°06’39"E	Phatthalung
504	PTLH-HB-C_4	7°49’19"N, 100°06’39"E	Phatthalung
505	PTLH-HB-C_5	7°49’19"N, 100°06’39"E	Phatthalung
506	PTLH-HB-C_6	7°49’19"N, 100°06’39"E	Phatthalung
507	PTLH-HB-C_7	7°49’19"N, 100°06’39"E	Phatthalung
508	PTLH-HB-C_8	7°49’19"N, 100°06’39"E	Phatthalung
509	PTLH-HB-C_9	7°49’19"N, 100°06’39"E	Phatthalung
510	PTLH-HB-C_10	7°49’19"N, 100°06’39"E	Phatthalung
511	PTLH-HB-C_11	7°49’19"N, 100°06’39"E	Phatthalung

Table S2 catfish specimens used in this study

Species	Population	Code	*N**	Location	District	Province	GPS coordinate
C. gariephinus	Nakhon Nayok	NYK-CG-C	26	Jeed Jad Farm	Mueang Nakhon Nayok	Nakhon Nayok	14˚05’58”N, 101˚09’39”E
	Kalasin 1	KSN1-CG-C	70	Kalasin Fish Farm (Betagro), Phu Sing	Mueang Kalasin	Kalasin	16˚39’29"N, 103˚29’10"E
	Kalasin 2	KSN2-CG-C	95	Kalasin Fish Farm (Betagro), Phu Sing	Mueang Kalasin	Kalasin	16˚39’29"N, 103˚29’10"E
C. macrocephalus	Sing Buri	SB-CM-C	2	Phakin fish species	In Buri	Sing Buri	14˚58’13"N, 100˚18’41"E
	Sakon Nakhon 1	SNK1-CM-W	30	Nong Han	Mueang Sakon Nakhon	Sakon Nakhon	17°21’14"N, 104°19’16"E
	Sakon Nakhon 2	SNK2-CM-W	136	Susarn Donkajo	Mueang Sakon Nakhon	Sakon Nakhon	17°14’05"N, 104°12’28"E
	Sakon Nakhon 3	SNK3-CM-W	63	Barnthavat	Mueang Sakon Nakhon	Sakon Nakhon	17°14’46"N, 104°23’82"E
	Suphan Buri 1	SPB1-CM-W	5	Ban Chang Subdistrict	Song Phi Nong	Suphan Buri	14°14’57.1"N, 100°11’13.0"E
	Suphan Buri 2	SPB2-CM-W	3	Khlong Bang Khan Chang, Bang Ta then subdistrict	Song Phi Nong	Suphan Buri	14°14’40.5"N, 100°07’60.0"E
	Nakhon Pathom	NPT1-CM-W	2	Khlong Mon Thong	Kamphaeng Saen	Nakhon Pathom	13°59’17.7"N, 99°56’37.3"E
	Ubon Ratchathani 1	UBR-CM-C	4	Ubon Ratchathani University	Warin Chamrap	Ubon Ratchathani	15°14’25"N, 104°51’23"E
	Chiang Rai	CR-CM-C	21	Bueng Bon	Wiang Chai	Chiang Rai	19°52’36"N, 99°55’42"E
	Yala	YLA-CM-C	19	Khlong Sa Mor	Raman	Yala	6°34’42"N, 101°25’47"E
	Sa Kaeo	SKW-CM-C	18	Khlong Phra Sathueng	Wang Sombun	Sa Kaeo	13°22’33"N, 102°07’46"E

Species	Population	Code	*N**	Location	District	Province	GPS coordinate
	Phang Nga	PLG-CM-C	3	Khlong Khamao	Nuea Khlong	Phang Nga	8°04’05.4"N, 98°59’29.1"E
C. batrachus	Ubon Ratchathani 2	UBR-CB-C	3	Ubon Ratchathani University	Warin Chamrap	Ubon Ratchathani	15°14’25"N, 104°51’23"E
HB	Phatthalung	PTLH-HB-C	11	Thale Noi	Khuan Khanun	Phatthalung	7°49’19"N, 100°06’39"E

**N*, number of samples

Table S3 List of teleost and *Myf5*-related sequences retrieved from public databases used for comparative genomic analysis

No.	Species	Accession number	Clade	Country
1	*Homo sapiens*	NM_005593.3	Mammal	Not specified (RefSeq)
2	*Mus musculus*	NM_008656.5	Mammal	Not specified (RefSeq)
3	*Sus scrofa*	KC456667.1	Mammal	Not specified (RefSeq)
4	*Ctenopharyngodon idella*	MG574982.1	Actinopterygii	China
5	*Schizothorax richardsonii*	OP272172.1	Actinopterygii	Not specified (RefSeq)
6	*Oncorhynchus mykiss*	AY751283.1	Actinopterygii	Not specified (RefSeq)
7	*Mugil cephalus*	XM_047575926.1	Actinopterygii	India
8	*Takifugu rubripes*	AY445319.1	Actinopterygii	Not specified (RefSeq)
9	*Epinephelus coioides*	HM190249.1	Actinopterygii	Not specified (RefSeq)
10	*Epinephelus lanceolatus*	MW762680.1	Actinopterygii	Not specified (RefSeq)
11	*Clarias batrachus*	KX258952.1	Siluriformes	India
12	*Ictalurus furcatus*	XM_053650830.1	Siluriformes	United States
13	*Ictalurus punctatus*	XM_017493744.3	Siluriformes	Not specified (RefSeq)
14	*Trichomycterus rosablanca*	XM_063007419.1	Siluriformes	Colombia
15	*Silurus meridionalis*	XM_046873826.1	Siluriformes	Not specified (RefSeq)
16	*Neoarius graeffei*	XM_060903546.1	Siluriformes	Australia
17	*Tachysurus vachellii*	XM_060889611.1	Siluriformes	China
18	*Tachysurus fulvidraco*	XM_027155249.2	Siluriformes	Not specified (RefSeq)
19	*Hemibagrus wyckioides*	XM_058417451.1	Siluriformes	Not specified (RefSeq)
20	*Pangasianodon hypophthalmus*	XM_026909928.3	Siluriformes	Germany

Tables S4 Variable sites in the sequences of *Myf5* haplotypes of catfish populations in Thailand

Haplotype	Variable nucleotide position (Accession number: XM053509129)																
	24	44	56	60	72	77	92	96	111	137	176	182	224	226	242	251	277
XM053509129	A	A	C	C	T	G	G	G	C	C	T	G	A	G	C	C	C
*Clarias_Myf5*TH1*	G	G	.	.	.	.	T	.	.	.	.	C	G	.	G	G	.
*Clarias_Myf5*TH2*	G	G	.	T	.	.	T	.	.	.	.	C	G	.	G	G	.
*Clarias_Myf5*TH3*	G	G	.	.	.	.	T	A	.	.	.	C	G	.	G	G	.
*Clarias_Myf5*TH4*	G	G	.	T	.	.	T	.	T	.	.	C	G	.	G	G	.
*Clarias_Myf5*TH5*	G	G	.	T	.	.	T	.	.	.	.	C	G	.	G	G	T
*Clarias_Myf5*TH6*	G	G	.	T	.	T	T	.	.	.	.	C	G	.	G	G	.
*Clarias_Myf5*TH7*	.	.	.	.	.	.	.	.	.	.	.	.	.	.	.	.	.
*Clarias_Myf5*TH8*	.	.	.	.	.	.	.	.	.	T	.	.	.	.	.	.	.
*Clarias_Myf5*TH9*	G	G	.	.	C	.	T	.	.	.	C	C	.	.	G	G	.
*Clarias_Myf5*TH10*	G	G	.	.	C	.	T	.	.	.	.	C	.	.	G	G	.
*Clarias_Myf5*TH11*	.	.	.	.	.	.	.	.	.	T	.	.	G	.	G	G	.
*Clarias_Myf5*TH12*	.	.	T	.	.	.	.	.	.	.	.	.	.	.	.	.	.
*Clarias_Myf5*TH13*	.	.	.	.	.	.	.	.	.	.	.	C	G	.	G	G	.
*Clarias_Myf5*TH14*	G	G	.	.	.	.	T	.	.	.	.	.	.	.	.	.	.
*Clarias_Myf5*TH15*	G	G	.	T	.	.	T	.	.	.	.	.	.	.	.	.	.
*Clarias_Myf5*TH16*	.	.	T	.	.	.	.	.	.	T	.	.	.	.	.	.	.
*Clarias_Myf5*TH17*	G	G	.	.	.	.	T	.	.	.	.	C	G	A	G	G	.

Table S5 Genetic diversity indices of catfish populations in Thailand based on the *Myf5* sequences

Species	Population	Code	*S*[1]	*h*[2]	*Hd*[3]	*k*[4]
C. gariephinus	Nakhon Nayok	NYK-CG-C	6	4	1.000	3.000
	Kalasin 1	KSN1-CG-C	6	4	1.000	3.000
	Kalasin 2	KSN2-CG-C	2	4	1.000	1.333
	Mean	-	6	5	1.000	2.800
C. macrocephalus	Sing Buri	SB-CM-C	8	4	1.000	4.667
	Sakon Nakhon 1	SNK1-CM-W	11	6	1.000	5.267
	Sakon Nakhon 2	SNK2-CM-W	11	8	1.000	4.857
	Sakon Nakhon 3	SNK3-CM-W	13	8	1.000	4.821
	Suphan Buri 1	SPB1-CM-W	-	-	-	-
	Suphan Buri 2	SPB2-CM-W	-	-	-	-
	Nakhon Pathom	NPT-CM-W	7	2	1.000	7.000
	Ubon Ratchathani 1	UBR-CM-C	1	2	1.000	1.000
	Chiang Rai	CR-CM-C	1	2	1.000	1.000
	Yala	YLA-CM-C	1	2	1.000	1.000
	Sa Kaeo	SKW-CM-C	-	-	-	-
	Phang Nga	PLG-CM-C	-	-	-	-
	Mean	-	17	15	1.000	5.162
C. batrachus	Ubon Ratchathani 2	UBR-CB-C	5	4	1.000	3.000
**Hybrid**	Phatthalung	PTLH-HB-C	10	5	1.000	5.400
	Overall mean value	-	17	17	1.000	5.368

[1] Polymorphic sites (*S*); [2] Number of haplotypes (*h*); [3] Haplotype diversity (*Hd*); [4] Average number of nucleotide differences (*k*)

Table S6 Genetic differentiation between catfish populations in Thailand based on the *Myf5* sequences

Population 1	Population 2	F_{ST}
NYK-CG-C	KSN1-CG-C	0.013
NYK-CG-C	KSN2-CG-C	0.000
NYK-CG-C	SBR-CM-C	0.476
NYK-CG-C	SNK1-CM-W	0.403
NYK-CG-C	SNK2-CM-W	0.499
NYK-CG-C	SNK3-CM-W	0.434
NYK-CG-C	SPB1-CM-W	0.691
NYK-CG-C	SPB2-CM-W	0.673
NYK-CG-C	NPT-CM-W	0.476
NYK-CG-C	UBR-CM-C	0.629
NYK-CG-C	UBR-CB-C	0.472
NYK-CG-C	CR-CM-C	0.735
NYK-CG-C	YLA-CM-C	0.746
NYK-CG-C	SKW-CM-C	0.764
NYK-CG-C	PLG-CM-C	0.673
NYK-CG-C	PTLH-HB-C	0.336
KSN1-CG-C	KSN2-CG-C	0.010
KSN1-CG-C	SBR-CM-C	0.518
KSN1-CG-C	SNK1-CM-W	0.466
KSN1-CG-C	SNK2-CM-W	0.528
KSN1-CG-C	SNK3-CM-W	0.480
KSN1-CG-C	SPB1-CM-W	0.701
KSN1-CG-C	SPB2-CM-W	0.692
KSN1-CG-C	NPT-CM-W	0.518
KSN1-CG-C	UBR-CM-C	0.655
KSN1-CG-C	UBR-CB-C	0.529
KSN1-CG-C	CR-CM-C	0.717
KSN1-CG-C	YLA-CM-C	0.725
KSN1-CG-C	SKW-CM-C	0.736
KSN1-CG-C	PLG-CM-C	0.692
KSN1-CG-C	PTLH-HB-C	0.413
KSN2-CG-C	SBR-CM-C	0.504
KSN2-CG-C	SNK1-CM-W	0.470
KSN2-CG-C	SNK2-CM-W	0.532
KSN2-CG-C	SNK3-CM-W	0.486
KSN2-CG-C	SPB1-CM-W	0.688
KSN2-CG-C	SPB2-CM-W	0.680
KSN2-CG-C	NPT-CM-W	0.504
KSN2-CG-C	UBR-CM-C	0.646
KSN2-CG-C	UBR-CB-C	0.518
KSN2-CG-C	CR-CM-C	0.701
KSN2-CG-C	YLA-CM-C	0.708
KSN2-CG-C	SKW-CM-C	0.718
KSN2-CG-C	PLG-CM-C	0.680
KSN2-CG-C	PTLH-HB-C	0.397
SBR-CM-C	SNK1-CM-W	0.152
SBR-CM-C	SNK2-CM-W	0.394

Population 1	Population 2	F_{ST}
SBR-CM-C	SNK3-CM-W	0.251
SBR-CM-C	SPB1-CM-W	0.270
SBR-CM-C	SPB2-CM-W	0.132
SBR-CM-C	NPT-CM-W	–0.333
SBR-CM-C	UBR-CM-C	0.630
SBR-CM-C	UBR-CB-C	0.315
SBR-CM-C	CR-CM-C	0.239
SBR-CM-C	YLA-CM-C	0.350
SBR-CM-C	SKW-CM-C	0.601
SBR-CM-C	PLG-CM-C	0.132
SBR-CM-C	PTLH-HB-C	0.007
SNK1-CM-W	SNK2-CM-W	0.037
SNK1-CM-W	SNK3-CM-W	–0.001
SNK1-CM-W	SPB1-CM-W	0.347
SNK1-CM-W	SPB2-CM-W	0.313
SNK1-CM-W	NPT-CM-W	0.152
SNK1-CM-W	UBR-CM-C	0.082
SNK1-CM-W	UBR-CB-C	0.158
SNK1-CM-W	CR-CM-C	0.414
SNK1-CM-W	YLA-CM-C	0.428
SNK1-CM-W	SKW-CM-C	0.447
SNK1-CM-W	PLG-CM-C	0.313
SNK1-CM-W	PTLH-HB-C	0.133
SNK2-CM-W	SNK3-CM-W	0.023
SNK2-CM-W	SPB1-CM-W	0.505
SNK2-CM-W	SPB2-CM-W	0.493
SNK2-CM-W	NPT-CM-W	0.394
SNK2-CM-W	UBR-CM-C	0.010
SNK2-CM-W	UBR-CB-C	0.283
SNK2-CM-W	CR-CM-C	0.513
SNK2-CM-W	YLA-CM-C	0.524
SNK2-CM-W	SKW-CM-C	0.535
SNK2-CM-W	PLG-CM-C	0.493
SNK2-CM-W	PTLH-HB-C	0.316
SNK3-CM-W	SPB1-CM-W	0.395
SNK3-CM-W	SPB2-CM-W	0.377
SNK3-CM-W	NPT-CM-W	0.251
SNK3-CM-W	UBR-CM-C	0.063
SNK3-CM-W	UBR-CB-C	0.234
SNK3-CM-W	CR-CM-C	0.425
SNK3-CM-W	YLA-CM-C	0.436
SNK3-CM-W	SKW-CM-C	0.450
SNK3-CM-W	PLG-CM-C	0.377
SNK3-CM-W	PTLH-HB-C	0.194
SPB1-CM-W	SPB2-CM-W	0.000
SPB1-CM-W	NPT-CM-W	0.270
SPB1-CM-W	UBR-CM-C	0.874
SPB1-CM-W	UBR-CB-C	0.671
SPB1-CM-W	CR-CM-C	–0.033

Population 1	Population 2	F_{ST}
SPB1-CM-W	YLA-CM-C	–0.050
SPB1-CM-W	SKW-CM-C	0.000
SPB1-CM-W	PLG-CM-C	0.000
SPB1-CM-W	PTLH-HB-C	0.250
SPB2-CM-W	NPT-CM-W	0.132
SPB2-CM-W	UBR-CM-C	0.836
SPB2-CM-W	UBR-CB-C	0.583
SPB2-CM-W	CR-CM-C	–0.072
SPB2-CM-W	YLA-CM-C	–0.091
SPB2-CM-W	SKW-CM-C	0.000
SPB2-CM-W	PLG-CM-C	0.000
SPB2-CM-W	PTLH-HB-C	0.207
NPT-CM-W	UBR-CM-C	0.630
NPT-CM-W	UBR-CB-C	0.315
NPT-CM-W	CR-CM-C	0.239
NPT-CM-W	YLA-CM-C	0.350
NPT-CM-W	SKW-CM-C	0.601
NPT-CM-W	PLG-CM-C	0.132
NPT-CM-W	PTLH-HB-C	0.007
UBR-CM-C	UBR-CB-C	0.395
UBR-CM-C	CR-CM-C	0.861
UBR-CM-C	YLA-CM-C	0.898
UBR-CM-C	SKW-CM-C	0.948
UBR-CM-C	PLG-CM-C	0.836
UBR-CM-C	PTLH-HB-C	0.402
UBR-CB-C	CR-CM-C	0.768
UBR-CB-C	YLA-CM-C	0.803
UBR-CB-C	SKW-CM-C	0.856
UBR-CB-C	PLG-CM-C	0.583
UBR-CB-C	PTLH-HB-C	0.233
CR-CM-C	YLA-CM-C	–0.002
CR-CM-C	SKW-CM-C	0.020
CR-CM-C	PLG-CM-C	–0.072
CR-CM-C	PTLH-HB-C	0.343
YLA-CM-C	SKW-CM-C	–0.001
YLA-CM-C	PLG-CM-C	–0.091
YLA-CM-C	PTLH-HB-C	0.365
SKW-CM-C	PLG-CM-C	0.000
SKW-CM-C	PTLH-HB-C	0.404
PLG-CM-C	PTLH-HB-C	0.207

Table S7 The results of Analysis of Variance (AMOVA) for catfish populations in Thailand

**Source**	**df**	**SS**	**Var**	**% of variance**
Among Population	18	10419.645	578.869	56
Among Individual	508	4519.905	8.897	24
Within Individual	610	3655.090	5.992	20
Total	1136	18594.640	593.759	100

(df: degree of freedom *f*; SS: sum of squares; Var: variance components)

Table S8 Comparison of observed (H_o) and expected heterozygosity (H_e) of the *Myf5* gene in catfish populations

Populations	Difference of means	Standard Error	*t*-test	*p*-value
NYK-CG-C	0.094	0	Inf	N/A
KSN1-CG-C	0.066	0	Inf	N/A
KSN2-CG-C	–0.006	0	Inf	N/A
SBR-CM-C	0.125	0	Inf	N/A
SNK1-CM-W	–0.126	0	Inf	N/A
SNK2-CM-W	0.057	0	Inf	N/A
SNK3-CM-W	0.112	0	Inf	N/A
SPB1-CM-W	0.000	0	Inf	N/A
SPB2-CM-W	0.000	0	Inf	N/A
NPT-CM-W	0.125	0	Inf	N/A
UBR-CM-C	0.031	0	Inf	N/A
CR-CM-C	0.004	0	Inf	N/A
YLA-CM-C	0.002	0	Inf	N/A
SKW-CM-C	0.000	0	Inf	N/A
PLG-CM-C	0.000	0	Inf	N/A
UBR-CB-C	0.278	0	Inf	N/A
PTLH-HB-C	0.293	0	Inf	N/A

Inf, infinitive; N/A, not applicable

Table S9 Comparison of expected heterozygosity (H_e) of the *Myf5* gene between catfish populations

Population 1	Population 2	Difference of means	Standard Error	*t*-test	*p*-value
NYK-CG-C	KSN1-CG-C	0.043	0	Inf	N/A
NYK-CG-C	KSN2-CG-C	0.032	0	Inf	N/A
NYK-CG-C	SBR-CM-C	0.031	0	Inf	N/A
NYK-CG-C	SNK1-CM-W	–0.287	0	Inf	N/A
NYK-CG-C	SNK2-CM-W	–0.133	0	Inf	N/A
NYK-CG-C	SNK3-CM-W	–0.212	0	Inf	N/A
NYK-CG-C	SPB1-CM-W	0.406	0	Inf	N/A
NYK-CG-C	SPB2-CM-W	0.406	0	Inf	N/A
NYK-CG-C	NPT-CM-W	0.031	0	Inf	N/A
NYK-CG-C	UBR-CM-C	0.187	0	Inf	N/A
NYK-CG-C	CR-CM-C	0.315	0	Inf	N/A
NYK-CG-C	YLA-CM-C	0.355	0	Inf	N/A
NYK-CG-C	SKW-CM-C	0.406	0	Inf	N/A
NYK-CG-C	PLG-CM-C	0.406	0	Inf	N/A
NYK-CG-C	UBR-CB-C	–0.316	0	Inf	N/A
NYK-CG-C	PTLH-HB-C	–0.301	0	Inf	N/A
KSN1-CG-C	KSN2-CG-C	–0.011	0	Inf	N/A
KSN1-CG-C	SBR-CM-C	–0.012	0	Inf	N/A
KSN1-CG-C	SNK1-CM-W	–0.330	0	Inf	N/A
KSN1-CG-C	SNK2-CM-W	–0.176	0	Inf	N/A
KSN1-CG-C	SNK3-CM-W	–0.255	0	Inf	N/A
KSN1-CG-C	SPB1-CM-W	0.363	0	Inf	N/A
KSN1-CG-C	SPB2-CM-W	0.363	0	Inf	N/A
KSN1-CG-C	NPT-CM-W	–0.012	0	Inf	N/A
KSN1-CG-C	UBR-CM-C	0.144	0	Inf	N/A
KSN1-CG-C	CR-CM-C	0.272	0	Inf	N/A
KSN1-CG-C	YLA-CM-C	0.312	0	Inf	N/A
KSN1-CG-C	SKW-CM-C	0.363	0	Inf	N/A
KSN1-CG-C	PLG-CM-C	0.363	0	Inf	N/A
KSN1-CG-C	UBR-CB-C	–0.359	0	Inf	N/A
KSN1-CG-C	PTLH-HB-C	–0.344	0	Inf	N/A
KSN2-CG-C	SBR-CM-C	–0.001	0	Inf	N/A
KSN2-CG-C	SNK1-CM-W	–0.319	0	Inf	N/A
KSN2-CG-C	SNK2-CM-W	–0.165	0	Inf	N/A
KSN2-CG-C	SNK3-CM-W	–0.244	0	Inf	N/A
KSN2-CG-C	SPB1-CM-W	0.374	0	Inf	N/A
KSN2-CG-C	SPB2-CM-W	0.374	0	Inf	N/A
KSN2-CG-C	NPT-CM-W	–0.001	0	Inf	N/A
KSN2-CG-C	UBR-CM-C	0.155	0	Inf	N/A
KSN2-CG-C	CR-CM-C	0.283	0	Inf	N/A
KSN2-CG-C	YLA-CM-C	0.323	0	Inf	N/A
KSN2-CG-C	SKW-CM-C	0.374	0	Inf	N/A
KSN2-CG-C	PLG-CM-C	0.374	0	Inf	N/A
KSN2-CG-C	UBR-CB-C	–0.348	0	Inf	N/A
KSN2-CG-C	PTLH-HB-C	–0.333	0	Inf	N/A
SBR-CM-C	SNK1-CM-W	–0.318	0	Inf	N/A
SBR-CM-C	SNK2-CM-W	–0.164	0	Inf	N/A
SBR-CM-C	SNK3-CM-W	–0.243	0	Inf	N/A
SBR-CM-C	SPB1-CM-W	0.375	0	Inf	N/A
SBR-CM-C	SPB2-CM-W	0.375	0	Inf	N/A
SBR-CM-C	NPT-CM-W	0.000	0	Inf	N/A
SBR-CM-C	UBR-CM-C	0.156	0	Inf	N/A

Population 1	Population 2	Difference of means	Standard Error	*t*-test	*p*-value
SBR-CM-C	CR-CM-C	0.284	0	Inf	N/A
SBR-CM-C	YLA-CM-C	0.324	0	Inf	N/A
SBR-CM-C	SKW-CM-C	0.375	0	Inf	N/A
SBR-CM-C	PLG-CM-C	0.375	0	Inf	N/A
SBR-CM-C	UBR-CB-C	–0.347	0	Inf	N/A
SBR-CM-C	PTLH-HB-C	–0.332	0	Inf	N/A
SNK1-CM-W	SNK2-CM-W	0.154	0	Inf	N/A
SNK1-CM-W	SNK3-CM-W	0.075	0	Inf	N/A
SNK1-CM-W	SPB1-CM-W	0.693	0	Inf	N/A
SNK1-CM-W	SPB2-CM-W	0.693	0	Inf	N/A
SNK1-CM-W	NPT-CM-W	0.318	0	Inf	N/A
SNK1-CM-W	UBR-CM-C	0.474	0	Inf	N/A
SNK1-CM-W	CR-CM-C	0.602	0	Inf	N/A
SNK1-CM-W	YLA-CM-C	0.642	0	Inf	N/A
SNK1-CM-W	SKW-CM-C	0.693	0	Inf	N/A
SNK1-CM-W	PLG-CM-C	0.693	0	Inf	N/A
SNK1-CM-W	UBR-CB-C	–0.029	0	Inf	N/A
SNK1-CM-W	PTLH-HB-C	–0.014	0	Inf	N/A
SNK2-CM-W	SNK3-CM-W	–0.079	0	Inf	N/A
SNK2-CM-W	SPB1-CM-W	0.539	0	Inf	N/A
SNK2-CM-W	SPB2-CM-W	0.539	0	Inf	N/A
SNK2-CM-W	NPT-CM-W	0.164	0	Inf	N/A
SNK2-CM-W	UBR-CM-C	0.320	0	Inf	N/A
SNK2-CM-W	CR-CM-C	0.448	0	Inf	N/A
SNK2-CM-W	YLA-CM-C	0.488	0	Inf	N/A
SNK2-CM-W	SKW-CM-C	0.539	0	Inf	N/A
SNK2-CM-W	PLG-CM-C	0.539	0	Inf	N/A
SNK2-CM-W	UBR-CB-C	–0.183	0	Inf	N/A
SNK2-CM-W	PTLH-HB-C	–0.168	0	Inf	N/A
SNK3-CM-W	SPB1-CM-W	0.618	0	Inf	N/A
SNK3-CM-W	SPB2-CM-W	0.618	0	Inf	N/A
SNK3-CM-W	NPT-CM-W	0.243	0	Inf	N/A
SNK3-CM-W	UBR-CM-C	0.399	0	Inf	N/A
SNK3-CM-W	CR-CM-C	0.527	0	Inf	N/A
SNK3-CM-W	YLA-CM-C	0.567	0	Inf	N/A
SNK3-CM-W	SKW-CM-C	0.618	0	Inf	N/A
SNK3-CM-W	PLG-CM-C	0.618	0	Inf	N/A
SNK3-CM-W	UBR-CB-C	–0.104	0	Inf	N/A
SNK3-CM-W	PTLH-HB-C	–0.089	0	Inf	N/A
SPB1-CM-W	SPB2-CM-W	0.000	0	Inf	N/A
SPB1-CM-W	NPT-CM-W	–0.375	0	Inf	N/A
SPB1-CM-W	UBR-CM-C	–0.219	0	Inf	N/A
SPB1-CM-W	CR-CM-C	–0.091	0	Inf	N/A
SPB1-CM-W	YLA-CM-C	–0.051	0	Inf	N/A
SPB1-CM-W	SKW-CM-C	0.000	0	Inf	N/A
SPB1-CM-W	PLG-CM-C	0.000	0	Inf	N/A
SPB1-CM-W	UBR-CB-C	–0.722	0	Inf	N/A
SPB1-CM-W	PTLH-HB-C	–0.707	0	Inf	N/A
SPB2-CM-W	NPT-CM-W	–0.375	0	Inf	N/A
SPB2-CM-W	UBR-CM-C	–0.219	0	Inf	N/A
SPB2-CM-W	CR-CM-C	–0.091	0	Inf	N/A
SPB2-CM-W	YLA-CM-C	–0.051	0	Inf	N/A
SPB2-CM-W	SKW-CM-C	0.000	0	Inf	N/A
SPB2-CM-W	PLG-CM-C	0.000	0	Inf	N/A

Population 1	Population 2	Difference of means	Standard Error	*t*-test	*p*-value
SPB2-CM-W	UBR-CB-C	–0.722	0	Inf	N/A
SPB2-CM-W	PTLH-HB-C	–0.707	0	Inf	N/A
NPT-CM-W	UBR-CM-C	0.156	0	Inf	N/A
NPT-CM-W	CR-CM-C	0.284	0	Inf	N/A
NPT-CM-W	YLA-CM-C	0.324	0	Inf	N/A
NPT-CM-W	SKW-CM-C	0.375	0	Inf	N/A
NPT-CM-W	PLG-CM-C	0.375	0	Inf	N/A
NPT-CM-W	UBR-CB-C	–0.347	0	Inf	N/A
NPT-CM-W	PTLH-HB-C	–0.332	0	Inf	N/A
UBR-CM-C	CR-CM-C	0.128	0	Inf	N/A
UBR-CM-C	YLA-CM-C	0.168	0	Inf	N/A
UBR-CM-C	SKW-CM-C	0.219	0	Inf	N/A
UBR-CM-C	PLG-CM-C	0.219	0	Inf	N/A
UBR-CM-C	UBR-CB-C	–0.503	0	Inf	N/A
UBR-CM-C	PTLH-HB-C	–0.488	0	Inf	N/A
CR-CM-C	YLA-CM-C	0.040	0	Inf	N/A
CR-CM-C	SKW-CM-C	0.091	0	Inf	N/A
CR-CM-C	PLG-CM-C	0.091	0	Inf	N/A
CR-CM-C	UBR-CB-C	–0.631	0	Inf	N/A
CR-CM-C	PTLH-HB-C	–0.616	0	Inf	N/A
YLA-CM-C	SKW-CM-C	0.051	0	Inf	N/A
YLA-CM-C	PLG-CM-C	0.051	0	Inf	N/A
YLA-CM-C	UBR-CB-C	–0.671	0	Inf	N/A
YLA-CM-C	PTLH-HB-C	–0.656	0	Inf	N/A
SKW-CM-C	PLG-CM-C	0.000	0	Inf	N/A
SKW-CM-C	UBR-CB-C	–0.722	0	Inf	N/A
SKW-CM-C	PTLH-HB-C	–0.707	0	Inf	N/A
PLG-CM-C	UBR-CB-C	–0.722	0	Inf	N/A
PLG-CM-C	PTLH-HB-C	–0.707	0	Inf	N/A
UBR-CB-C	PTLH-HB-C	0.015	0	Inf	N/A

Inf, infinitive; N/A, not applicable

Table S10 Comparison of observed heterozygosity (H_o) for the *Myf5* gene between catfish populations

Population 1	Population 2	Difference of means	Standard Error	*t*-test	*p*-value
NYK-CG-C	KSN1-CG-C	0.071	0	Inf	N/A
NYK-CG-C	KSN2-CG-C	0.132	0	Inf	N/A
NYK-CG-C	SBR-CM-C	0.000	0	Inf	N/A
NYK-CG-C	SNK1-CM-W	–0.067	0	Inf	N/A
NYK-CG-C	SNK2-CM-W	–0.096	0	Inf	N/A
NYK-CG-C	SNK3-CM-W	–0.230	0	Inf	N/A
NYK-CG-C	SPB1-CM-W	0.500	0	Inf	N/A
NYK-CG-C	SPB2-CM-W	0.500	0	Inf	N/A
NYK-CG-C	NPT-CM-W	0.000	0	Inf	N/A
NYK-CG-C	UBR-CM-C	0.250	0	Inf	N/A
NYK-CG-C	CR-CM-C	0.405	0	Inf	N/A
NYK-CG-C	YLA-CM-C	0.447	0	Inf	N/A
NYK-CG-C	SKW-CM-C	0.500	0	Inf	N/A
NYK-CG-C	PLG-CM-C	0.500	0	Inf	N/A
NYK-CG-C	UBR-CB-C	–0.500	0	Inf	N/A
NYK-CG-C	PTLH-HB-C	–0.500	0	Inf	N/A
KSN1-CG-C	KSN2-CG-C	0.061	0	Inf	N/A
KSN1-CG-C	SBR-CM-C	–0.071	0	Inf	N/A
KSN1-CG-C	SNK1-CM-W	–0.138	0	Inf	N/A
KSN1-CG-C	SNK2-CM-W	–0.167	0	Inf	N/A
KSN1-CG-C	SNK3-CM-W	–0.301	0	Inf	N/A
KSN1-CG-C	SPB1-CM-W	0.429	0	Inf	N/A
KSN1-CG-C	SPB2-CM-W	0.429	0	Inf	N/A
KSN1-CG-C	NPT-CM-W	–0.071	0	Inf	N/A
KSN1-CG-C	UBR-CM-C	0.179	0	Inf	N/A
KSN1-CG-C	CR-CM-C	0.334	0	Inf	N/A
KSN1-CG-C	YLA-CM-C	0.376	0	Inf	N/A
KSN1-CG-C	SKW-CM-C	0.429	0	Inf	N/A
KSN1-CG-C	PLG-CM-C	0.429	0	Inf	N/A
KSN1-CG-C	UBR-CB-C	–0.571	0	Inf	N/A
KSN1-CG-C	PTLH-HB-C	–0.571	0	Inf	N/A
KSN2-CG-C	SBR-CM-C	–0.132	0	Inf	N/A
KSN2-CG-C	SNK1-CM-W	–0.199	0	Inf	N/A
KSN2-CG-C	SNK2-CM-W	–0.228	0	Inf	N/A
KSN2-CG-C	SNK3-CM-W	–0.362	0	Inf	N/A
KSN2-CG-C	SPB1-CM-W	0.368	0	Inf	N/A
KSN2-CG-C	SPB2-CM-W	0.368	0	Inf	N/A
KSN2-CG-C	NPT-CM-W	–0.132	0	Inf	N/A
KSN2-CG-C	UBR-CM-C	0.118	0	Inf	N/A
KSN2-CG-C	CR-CM-C	0.273	0	Inf	N/A
KSN2-CG-C	YLA-CM-C	0.315	0	Inf	N/A
KSN2-CG-C	SKW-CM-C	0.368	0	Inf	N/A
KSN2-CG-C	PLG-CM-C	0.368	0	Inf	N/A
KSN2-CG-C	UBR-CB-C	–0.632	0	Inf	N/A
KSN2-CG-C	PTLH-HB-C	–0.632	0	Inf	N/A
SBR-CM-C	SNK1-CM-W	–0.067	0	Inf	N/A
SBR-CM-C	SNK2-CM-W	–0.096	0	Inf	N/A
SBR-CM-C	SNK3-CM-W	–0.230	0	Inf	N/A
SBR-CM-C	SPB1-CM-W	0.500	0	Inf	N/A
SBR-CM-C	SPB2-CM-W	0.500	0	Inf	N/A
SBR-CM-C	NPT-CM-W	0.000	0	Inf	N/A
SBR-CM-C	UBR-CM-C	0.250	0	Inf	N/A

Population 1	Population 2	Difference of means	Standard Error	*t*-test	*p*-value
SBR-CM-C	CR-CM-C	0.405	0	Inf	N/A
SBR-CM-C	YLA-CM-C	0.447	0	Inf	N/A
SBR-CM-C	SKW-CM-C	0.500	0	Inf	N/A
SBR-CM-C	PLG-CM-C	0.500	0	Inf	N/A
SBR-CM-C	UBR-CB-C	–0.500	0	Inf	N/A
SBR-CM-C	PTLH-HB-C	–0.500	0	Inf	N/A
SNK1-CM-W	SNK2-CM-W	–0.029	0	Inf	N/A
SNK1-CM-W	SNK3-CM-W	–0.163	0	Inf	N/A
SNK1-CM-W	SPB1-CM-W	0.567	0	Inf	N/A
SNK1-CM-W	SPB2-CM-W	0.567	0	Inf	N/A
SNK1-CM-W	NPT-CM-W	0.067	0	Inf	N/A
SNK1-CM-W	UBR-CM-C	0.317	0	Inf	N/A
SNK1-CM-W	CR-CM-C	0.472	0	Inf	N/A
SNK1-CM-W	YLA-CM-C	0.514	0	Inf	N/A
SNK1-CM-W	SKW-CM-C	0.567	0	Inf	N/A
SNK1-CM-W	PLG-CM-C	0.567	0	Inf	N/A
SNK1-CM-W	UBR-CB-C	–0.433	0	Inf	N/A
SNK1-CM-W	PTLH-HB-C	–0.433	0	Inf	N/A
SNK2-CM-W	SNK3-CM-W	–0.134	0	Inf	N/A
SNK2-CM-W	SPB1-CM-W	0.596	0	Inf	N/A
SNK2-CM-W	SPB2-CM-W	0.596	0	Inf	N/A
SNK2-CM-W	NPT-CM-W	0.096	0	Inf	N/A
SNK2-CM-W	UBR-CM-C	0.346	0	Inf	N/A
SNK2-CM-W	CR-CM-C	0.501	0	Inf	N/A
SNK2-CM-W	YLA-CM-C	0.543	0	Inf	N/A
SNK2-CM-W	SKW-CM-C	0.596	0	Inf	N/A
SNK2-CM-W	PLG-CM-C	0.596	0	Inf	N/A
SNK2-CM-W	UBR-CB-C	–0.404	0	Inf	N/A
SNK2-CM-W	PTLH-HB-C	–0.404	0	Inf	N/A
SNK3-CM-W	SPB1-CM-W	0.730	0	Inf	N/A
SNK3-CM-W	SPB2-CM-W	0.730	0	Inf	N/A
SNK3-CM-W	NPT-CM-W	0.230	0	Inf	N/A
SNK3-CM-W	UBR-CM-C	0.480	0	Inf	N/A
SNK3-CM-W	CR-CM-C	0.635	0	Inf	N/A
SNK3-CM-W	YLA-CM-C	0.677	0	Inf	N/A
SNK3-CM-W	SKW-CM-C	0.730	0	Inf	N/A
SNK3-CM-W	PLG-CM-C	0.730	0	Inf	N/A
SNK3-CM-W	UBR-CB-C	–0.270	0	Inf	N/A
SNK3-CM-W	PTLH-HB-C	–0.270	0	Inf	N/A
SPB1-CM-W	SPB2-CM-W	0.000	0	Inf	N/A
SPB1-CM-W	NPT-CM-W	–0.500	0	Inf	N/A
SPB1-CM-W	UBR-CM-C	–0.250	0	Inf	N/A
SPB1-CM-W	CR-CM-C	–0.095	0	Inf	N/A
SPB1-CM-W	YLA-CM-C	–0.053	0	Inf	N/A
SPB1-CM-W	SKW-CM-C	0.000	0	Inf	N/A
SPB1-CM-W	PLG-CM-C	0.000	0	Inf	N/A
SPB1-CM-W	UBR-CB-C	–1.000	0	Inf	N/A
SPB1-CM-W	PTLH-HB-C	–1.000	0	Inf	N/A
SPB2-CM-W	NPT-CM-W	–0.500	0	Inf	N/A
SPB2-CM-W	UBR-CM-C	–0.250	0	Inf	N/A
SPB2-CM-W	CR-CM-C	–0.095	0	Inf	N/A
SPB2-CM-W	YLA-CM-C	–0.053	0	Inf	N/A
SPB2-CM-W	SKW-CM-C	0.000	0	Inf	N/A
SPB2-CM-W	PLG-CM-C	0.000	0	Inf	N/A

Population 1	Population 2	Difference of means	Standard Error	*t*-test	*p*-value
SPB2-CM-W	UBR-CB-C	–1.000	0	Inf	N/A
SPB2-CM-W	PTLH-HB-C	–1.000	0	Inf	N/A
NPT-CM-W	UBR-CM-C	0.250	0	Inf	N/A
NPT-CM-W	CR-CM-C	0.405	0	Inf	N/A
NPT-CM-W	YLA-CM-C	0.447	0	Inf	N/A
NPT-CM-W	SKW-CM-C	0.500	0	Inf	N/A
NPT-CM-W	PLG-CM-C	0.500	0	Inf	N/A
NPT-CM-W	UBR-CB-C	–0.500	0	Inf	N/A
NPT-CM-W	PTLH-HB-C	–0.500	0	Inf	N/A
UBR-CM-C	CR-CM-C	0.155	0	Inf	N/A
UBR-CM-C	YLA-CM-C	0.197	0	Inf	N/A
UBR-CM-C	SKW-CM-C	0.250	0	Inf	N/A
UBR-CM-C	PLG-CM-C	0.250	0	Inf	N/A
UBR-CM-C	UBR-CB-C	–0.750	0	Inf	N/A
UBR-CM-C	PTLH-HB-C	–0.750	0	Inf	N/A
CR-CM-C	YLA-CM-C	0.042	0	Inf	N/A
CR-CM-C	SKW-CM-C	0.095	0	Inf	N/A
CR-CM-C	PLG-CM-C	0.095	0	Inf	N/A
CR-CM-C	UBR-CB-C	–0.905	0	Inf	N/A
CR-CM-C	PTLH-HB-C	–0.905	0	Inf	N/A
YLA-CM-C	SKW-CM-C	0.053	0	Inf	N/A
YLA-CM-C	PLG-CM-C	0.053	0	Inf	N/A
YLA-CM-C	UBR-CB-C	–0.947	0	Inf	N/A
YLA-CM-C	PTLH-HB-C	–0.947	0	Inf	N/A
SKW-CM-C	PLG-CM-C	0.000	0	Inf	N/A
SKW-CM-C	UBR-CB-C	–1.000	0	Inf	N/A
SKW-CM-C	PTLH-HB-C	–1.000	0	Inf	N/A
PLG-CM-C	UBR-CB-C	–1.000	0	Inf	N/A
PLG-CM-C	PTLH-HB-C	–1.000	0	Inf	N/A
UBR-CB-C	PTLH-HB-C	0.000	0	Inf	N/A

Inf, infinitive; N/A, not applicable

Table S11 Detailed site-by-site results from the Mixed Effects Model of Evolution (MEME) analysis

Codon	α	β1	p1	β+	p+	LRT	*p*-value	*q*	Class
1	0.000	0.000	1.000	0.000	0.000	0.000	1.000	1	Invariable
2	0.000	0.000	1.000	0.000	0.000	0.000	1.000	1	Invariable
3	0.000	0.000	1.000	0.000	0.000	0.000	1.000	1	Invariable
4	0.000	0.000	1.000	0.000	0.000	0.000	1.000	1	Invariable
5	0.000	0.000	1.000	0.000	0.000	0.000	1.000	1	Invariable
6	121.322	0.000	1.000	1.606	0.000	0.000	0.667	1	Neutral
7	0.000	0.000	1.000	0.000	0.000	0.000	1.000	1	Invariable
8	0.000	0.000	1.000	0.000	0.000	0.000	1.000	1	Invariable
9	0.000	0.000	1.000	0.000	0.000	0.000	1.000	1	Invariable
10	82.746	0.000	1.000	9.996	0.000	0.000	0.667	1	Neutral
11	0.000	0.000	1.000	0.000	0.000	0.000	1.000	1	Invariable
12	78.487	0.000	0.976	0.436	0.024	0.000	0.667	1	Neutral
13	0.000	0.000	1.000	0.000	0.000	0.000	1.000	1	Invariable
14	0.000	0.000	1.000	0.000	0.000	0.000	1.000	1	Invariable
15	0.000	0.000	1.000	0.000	0.000	0.000	1.000	1	Invariable
16	0.025	0.001	0.010	19.983	0.990	0.446	0.445	1	Neutral
17	82.746	0.000	1.000	9.996	0.000	0.000	0.667	1	Neutral
18	0.000	0.000	1.000	0.000	0.000	0.000	1.000	1	Invariable
19	0.000	0.000	1.000	0.000	0.000	0.000	1.000	1	Invariable
20	0.000	0.000	1.000	0.000	0.000	0.000	1.000	1	Invariable
21	0.000	0.000	1.000	0.000	0.000	0.000	1.000	1	Invariable
22	47.535	0.000	1.000	2.944	0.000	0.000	0.667	1	Neutral
23	0.000	0.000	1.000	0.000	0.000	0.000	1.000	1	Invariable
24	0.016	0.008	0.781	147.595	0.219	0.236	0.514	1	Neutral
25	0.000	0.000	1.000	0.000	0.000	0.000	1.000	1	Invariable
26	0.000	0.000	1.000	0.000	0.000	0.000	1.000	1	Invariable
27	0.000	0.000	1.000	0.000	0.000	0.000	1.000	1	Invariable
28	0.000	0.000	1.000	0.000	0.000	0.000	1.000	1	Invariable
29	0.014	0.007	0.816	146.203	0.184	0.445	0.445	1	Neutral
30	0.000	0.000	1.000	0.000	0.000	0.000	1.000	1	Invariable
31	0.000	0.000	1.000	0.000	0.000	0.000	1.000	1	Invariable
32	0.000	0.000	1.000	0.000	0.000	0.000	1.000	1	Invariable
33	0.000	0.000	1.000	0.000	0.000	0.000	1.000	1	Invariable
34	0.000	0.000	1.000	0.000	0.000	0.000	1.000	1	Invariable
35	0.000	0.000	1.000	0.000	0.000	0.000	1.000	1	Invariable
36	0.000	0.000	1.000	0.000	0.000	0.000	1.000	1	Invariable
37	289.156	0.000	0.727	0.009	0.273	0.000	0.667	1	Neutral
38	0.000	0.000	1.000	0.000	0.000	0.000	1.000	1	Invariable
39	0.000	0.000	1.000	0.000	0.000	0.000	1.000	1	Invariable
40	0.000	0.000	1.000	0.000	0.000	0.000	1.000	1	Invariable
41	0.000	0.000	1.000	0.000	0.000	0.000	1.000	1	Invariable

Codon	α	β1	p1	β+	p+	LRT	*p*-value	*q*	Class
42	0.000	0.000	1.000	0.000	0.000	0.000	1.000	1	Invariable
43	0.000	0.000	1.000	0.000	0.000	0.000	1.000	1	Invariable
44	0.000	0.000	1.000	0.000	0.000	0.000	1.000	1	Invariable
45	0.000	0.000	1.000	0.000	0.000	0.000	1.000	1	Invariable
46	0.000	0.000	1.000	0.000	0.000	0.000	1.000	1	Invariable
47	0.000	0.000	1.000	0.000	0.000	0.000	1.000	1	Invariable
48	0.000	0.000	1.000	0.000	0.000	0.000	1.000	1	Invariable
49	0.000	0.000	1.000	0.000	0.000	0.000	1.000	1	Invariable
50	17.710	0.000	1.000	1.577	0.000	0.000	0.667	1	Neutral
51	0.000	0.000	1.000	0.000	0.000	0.000	1.000	1	Invariable
52	256.698	0.000	0.756	0.780	0.244	0.000	0.667	1	Neutral
53	0.000	0.000	1.000	0.000	0.000	0.000	1.000	1	Invariable
54	0.000	0.000	1.000	0.000	0.000	0.000	1.000	1	Invariable
55	0.000	0.000	1.000	0.000	0.000	0.000	1.000	1	Invariable
56	0.000	0.000	1.000	0.000	0.000	0.000	1.000	1	Invariable
57	0.000	0.000	1.000	0.000	0.000	0.000	1.000	1	Invariable
58	0.000	0.000	1.000	0.000	0.000	0.000	1.000	1	Invariable
59	0.000	0.000	1.000	0.000	0.000	0.000	1.000	1	Invariable
60	0.000	0.000	1.000	0.000	0.000	0.000	1.000	1	Invariable
61	0.000	0.000	1.000	0.000	0.000	0.000	1.000	1	Invariable
62	0.000	0.000	1.000	0.000	0.000	0.000	1.000	1	Invariable
63	0.000	0.000	1.000	0.000	0.000	0.000	1.000	1	Invariable
64	0.000	0.000	1.000	0.000	0.000	0.000	1.000	1	Invariable
65	0.000	0.000	1.000	0.000	0.000	0.000	1.000	1	Invariable
66	140.454	0.000	1.000	3.278	0.000	0.000	0.667	1	Neutral
67	0.004	0.002	0.802	161.906	0.198	0.459	0.441	1	Neutral
68	0.000	0.000	1.000	0.000	0.000	0.000	1.000	1	Invariable
69	0.000	0.000	1.000	0.000	0.000	0.000	1.000	1	Invariable
70	0.000	0.000	1.000	0.000	0.000	0.000	1.000	1	Invariable
71	0.000	0.000	1.000	0.000	0.000	0.000	1.000	1	Invariable
72	105.048	0.000	1.000	12.872	0.000	0.000	0.667	1	Neutral
73	0.000	0.000	1.000	0.000	0.000	0.000	1.000	1	Invariable
74	0.000	0.000	1.000	0.000	0.000	0.000	1.000	1	Invariable
75	105.414	0.000	1.000	11.744	0.000	0.000	0.667	1	Neutral
76	0.000	0.000	1.000	0.000	0.000	0.000	1.000	1	Invariable
77	0.000	0.000	1.000	0.000	0.000	0.000	1.000	1	Invariable
78	0.000	0.000	1.000	0.000	0.000	0.000	1.000	1	Invariable
79	0.000	0.000	1.000	0.000	0.000	0.000	1.000	1	Invariable
80	0.000	0.000	1.000	0.000	0.000	0.000	1.000	1	Invariable
81	0.000	0.000	1.000	0.000	0.000	0.000	1.000	1	Invariable
82	0.000	0.000	1.000	0.000	0.000	0.000	1.000	1	Invariable
83	0.000	0.000	1.000	0.000	0.000	0.000	1.000	1	Invariable
84	0.017	0.009	0.817	155.060	0.183	0.469	0.438	1	Neutral

Codon	α	β1	p1	β+	p+	LRT	*p*-value	*q*	Class
85	0.000	0.000	1.000	0.000	0.000	0.000	1.000	1	Invariable
86	0.000	0.000	1.000	0.000	0.000	0.000	1.000	1	Invariable
87	0.000	0.000	1.000	0.000	0.000	0.000	1.000	1	Invariable
88	0.000	0.000	1.000	0.000	0.000	0.000	1.000	1	Invariable
89	0.000	0.000	1.000	0.000	0.000	0.000	1.000	1	Invariable
90	0.000	0.000	1.000	0.000	0.000	0.000	1.000	1	Invariable
91	0.000	0.000	1.000	0.000	0.000	0.000	1.000	1	Invariable
92	0.000	0.000	1.000	0.000	0.000	0.000	1.000	1	Invariable
93	0.000	0.000	1.000	0.000	0.000	0.000	1.000	1	Invariable
94	0.000	0.000	1.000	0.000	0.000	0.000	1.000	1	Invariable
95	0.000	0.000	1.000	0.000	0.000	0.000	1.000	1	Invariable
96	0.000	0.000	1.000	0.000	0.000	0.000	1.000	1	Invariable
97	0.000	0.000	1.000	0.000	0.000	0.000	1.000	1	Invariable
98	0.000	0.000	1.000	0.000	0.000	0.000	1.000	1	Invariable
99	0.000	0.000	1.000	0.000	0.000	0.000	1.000	1	Invariable
100	0.000	0.000	1.000	0.000	0.000	0.000	1.000	1	Invariable
101	0.000	0.000	1.000	0.000	0.000	0.000	1.000	1	Invariable
102	0.000	0.000	1.000	0.000	0.000	0.000	1.000	1	Invariable
103	0.000	0.000	1.000	0.000	0.000	0.000	1.000	1	Invariable

Table S12 Detailed site-by-site results from the Fixed Effects Likelihood (FEL) analysis

Codon	α	β	α = β	LRT	*p*-value	Total branch length	Class
1	0.000	0.000	0.000	0.000	1.000	0.000	Invariable
2	0.000	0.000	0.000	0.000	1.000	0.000	Invariable
3	0.000	0.000	0.000	0.000	1.000	0.000	Invariable
4	0.000	0.000	0.000	0.000	1.000	0.000	Invariable
5	0.000	0.000	0.000	0.000	1.000	0.000	Invariable
6	8.838	0.002	2.141	5.502	0.019	0.649	Neutral
7	0.000	0.000	0.000	0.000	1.000	0.000	Invariable
8	0.000	0.000	0.000	0.000	1.000	0.000	Invariable
9	0.000	0.000	0.000	0.000	1.000	0.000	Invariable
10	5.913	0.000	1.111	3.256	0.071	0.337	Neutral
11	0.000	0.000	0.000	0.000	1.000	0.000	Invariable
12	5.681	0.000	2.220	3.564	0.059	0.673	Neutral
13	0.000	0.000	0.000	0.000	1.000	0.000	Invariable
14	0.000	0.000	0.000	0.000	1.000	0.000	Invariable
15	0.000	0.000	0.000	0.000	1.000	0.000	Invariable
16	0.000	1.434	1.147	0.447	0.504	0.348	Neutral
17	5.913	0.000	1.113	3.260	0.071	0.338	Neutral
18	0.000	0.000	0.000	0.000	1.000	0.000	Invariable
19	0.000	0.000	0.000	0.000	1.000	0.000	Invariable
20	0.000	0.000	0.000	0.000	1.000	0.000	Invariable
21	0.000	0.000	0.000	0.000	1.000	0.000	Invariable
22	3.393	0.000	1.527	3.170	0.075	0.463	Neutral
23	0.000	0.000	0.000	0.000	1.000	0.000	Invariable
24	0.000	1.507	1.396	0.148	0.700	0.423	Neutral
25	0.000	0.000	0.000	0.000	1.000	0.000	Invariable
26	0.000	0.000	0.000	0.000	1.000	0.000	Invariable
27	0.000	0.000	0.000	0.000	1.000	0.000	Invariable
28	0.000	0.000	0.000	0.000	1.000	0.000	Invariable
29	0.000	1.319	1.070	0.404	0.525	0.325	Neutral
30	0.000	0.000	0.000	0.000	1.000	0.000	Invariable
31	0.000	0.000	0.000	0.000	1.000	0.000	Invariable
32	0.000	0.000	0.000	0.000	1.000	0.000	Invariable
33	0.000	0.000	0.000	0.000	1.000	0.000	Invariable
34	0.000	0.000	0.000	0.000	1.000	0.000	Invariable
35	0.000	0.000	0.000	0.000	1.000	0.000	Invariable
36	0.000	0.000	0.000	0.000	1.000	0.000	Invariable
37	20.624	0.000	3.559	9.757	0.002	1.079	Purifying
38	0.000	0.000	0.000	0.000	1.000	0.000	Invariable
39	0.000	0.000	0.000	0.000	1.000	0.000	Invariable
40	0.000	0.000	0.000	0.000	1.000	0.000	Invariable
41	0.000	0.000	0.000	0.000	1.000	0.000	Invariable
42	0.000	0.000	0.000	0.000	1.000	0.000	Invariable

Codon	α	β	α = β	LRT	*p*-value	Total branch length	Class
43	0.000	0.000	0.000	0.000	1.000	0.000	Invariable
44	0.000	0.000	0.000	0.000	1.000	0.000	Invariable
45	0.000	0.000	0.000	0.000	1.000	0.000	Invariable
46	0.000	0.000	0.000	0.000	1.000	0.000	Invariable
47	0.000	0.000	0.000	0.000	1.000	0.000	Invariable
48	0.000	0.000	0.000	0.000	1.000	0.000	Invariable
49	0.000	0.000	0.000	0.000	1.000	0.000	Invariable
50	1.264	0.000	0.638	1.366	0.243	0.193	Neutral
51	0.000	0.000	0.000	0.000	1.000	0.000	Invariable
52	18.482	0.000	2.528	7.735	0.005	0.767	Purifying
53	0.000	0.000	0.000	0.000	1.000	0.000	Invariable
54	0.000	0.000	0.000	0.000	1.000	0.000	Invariable
55	0.000	0.000	0.000	0.000	1.000	0.000	Invariable
56	0.000	0.000	0.000	0.000	1.000	0.000	Invariable
57	0.000	0.000	0.000	0.000	1.000	0.000	Invariable
58	0.000	0.000	0.000	0.000	1.000	0.000	Invariable
59	0.000	0.000	0.000	0.000	1.000	0.000	Invariable
60	0.000	0.000	0.000	0.000	1.000	0.000	Invariable
61	0.000	0.000	0.000	0.000	1.000	0.000	Invariable
62	0.000	0.000	0.000	0.000	1.000	0.000	Invariable
63	0.000	0.000	0.000	0.000	1.000	0.000	Invariable
64	0.000	0.000	0.000	0.000	1.000	0.000	Invariable
65	0.000	0.000	0.000	0.000	1.000	0.000	Invariable
66	10.265	0.000	1.923	6.576	0.010	0.583	Neutral
67	0.000	1.312	1.105	0.347	0.556	0.335	Neutral
68	0.000	0.000	0.000	0.000	1.000	0.000	Invariable
69	0.000	0.000	0.000	0.000	1.000	0.000	Invariable
70	0.000	0.000	0.000	0.000	1.000	0.000	Invariable
71	0.000	0.000	0.000	0.000	1.000	0.000	Invariable
72	7.392	0.000	0.989	3.988	0.046	0.300	Neutral
73	0.000	0.000	0.000	0.000	1.000	0.000	Invariable
74	0.000	0.000	0.000	0.000	1.000	0.000	Invariable
75	7.454	0.000	1.206	3.597	0.058	0.366	Neutral
76	0.000	0.000	0.000	0.000	1.000	0.000	Invariable
77	0.000	0.000	0.000	0.000	1.000	0.000	Invariable
78	0.000	0.000	0.000	0.000	1.000	0.000	Invariable
79	0.000	0.000	0.000	0.000	1.000	0.000	Invariable
80	0.000	0.000	0.000	0.000	1.000	0.000	Invariable
81	0.000	0.000	0.000	0.000	1.000	0.000	Invariable
82	0.000	0.000	0.000	0.000	1.000	0.000	Invariable
83	0.000	0.000	0.000	0.000	1.000	0.000	Invariable
84	0.000	1.414	1.122	0.445	0.505	0.340	Neutral
85	0.000	0.000	0.000	0.000	1.000	0.000	Invariable

Codon	α	β	α = β	LRT	*p*-value	Total branch length	Class
86	0.000	0.000	0.000	0.000	1.000	0.000	Invariable
87	0.000	0.000	0.000	0.000	1.000	0.000	Invariable
88	0.000	0.000	0.000	0.000	1.000	0.000	Invariable
89	0.000	0.000	0.000	0.000	1.000	0.000	Invariable
90	0.000	0.000	0.000	0.000	1.000	0.000	Invariable
91	0.000	0.000	0.000	0.000	1.000	0.000	Invariable
92	0.000	0.000	0.000	0.000	1.000	0.000	Invariable
93	0.000	0.000	0.000	0.000	1.000	0.000	Invariable
94	0.000	0.000	0.000	0.000	1.000	0.000	Invariable
95	0.000	0.000	0.000	0.000	1.000	0.000	Invariable
96	0.000	0.000	0.000	0.000	1.000	0.000	Invariable
97	0.000	0.000	0.000	0.000	1.000	0.000	Invariable
98	0.000	0.000	0.000	0.000	1.000	0.000	Invariable
99	0.000	0.000	0.000	0.000	1.000	0.000	Invariable
100	0.000	0.000	0.000	0.000	1.000	0.000	Invariable
101	0.000	0.000	0.000	0.000	1.000	0.000	Invariable
102	0.000	0.000	0.000	0.000	1.000	0.000	Invariable
103	0.000	0.000	0.000	0.000	1.000	0.000	Invariable

Table S13 Codon sites under selection identified by Fast, Unconstrained Bayesian AppRoximation (FUBAR) analysis

Codon	α	β	β–α	Prob[α>β]	Prob[α<β]	BayesFactor[α<β]
1	5.518	0.724	–4.794	0.592	0.357	0.700
2	3.642	0.855	–2.787	0.551	0.396	0.824
3	2.263	0.734	–1.530	0.535	0.409	0.871
4	5.518	0.744	–4.773	0.590	0.359	0.706
5	0.770	0.923	0.152	0.461	0.479	1.158
6	33.098	0.733	–32.365	0.995	0.002	0.003
7	0.884	0.666	–0.218	0.494	0.445	1.010
8	3.643	0.826	–2.817	0.553	0.393	0.816
9	3.642	0.855	–2.787	0.551	0.396	0.824
10	17.238	0.727	–16.510	0.910	0.071	0.096
11	2.303	0.734	–1.569	0.537	0.407	0.865
12	22.954	0.860	–22.094	0.981	0.010	0.013
13	5.518	0.724	–4.794	0.592	0.357	0.700
14	3.645	0.659	–2.986	0.570	0.377	0.760
15	3.642	0.855	–2.787	0.551	0.396	0.824
16	2.282	3.347	1.065	0.275	0.674	2.601
17	19.531	0.727	–18.804	0.923	0.060	0.081
18	2.303	0.744	–1.559	0.535	0.409	0.870
19	4.646	0.942	–3.705	0.564	0.385	0.788
20	1.141	0.902	–0.239	0.487	0.455	1.049
21	4.647	0.895	–3.752	0.566	0.382	0.779
22	16.018	0.726	–15.292	0.972	0.017	0.022
23	3.642	0.855	–2.787	0.551	0.396	0.824
24	4.606	5.652	1.046	0.287	0.663	2.478
25	3.642	0.855	–2.787	0.551	0.396	0.824
26	4.647	0.895	–3.752	0.566	0.382	0.779
27	3.643	0.826	–2.817	0.553	0.393	0.816
28	2.263	0.744	–1.519	0.534	0.410	0.875
29	2.317	3.812	1.496	0.265	0.685	2.734
30	2.263	0.810	–1.452	0.528	0.416	0.897
31	2.263	0.786	–1.477	0.530	0.414	0.889
32	2.302	0.795	–1.507	0.531	0.414	0.887
33	3.643	0.812	–2.830	0.554	0.393	0.814
34	2.260	0.726	–1.534	0.536	0.408	0.868
35	3.643	0.826	–2.817	0.553	0.393	0.816
36	4.647	0.895	–3.752	0.566	0.382	0.779
37	39.276	0.802	–38.474	0.999	0.000	0.000
38	2.302	0.795	–1.507	0.531	0.414	0.887
39	3.643	0.812	–2.830	0.554	0.393	0.814
40	3.644	0.687	–2.957	0.566	0.380	0.773
41	1.141	0.902	–0.239	0.487	0.455	1.049
42	4.645	1.092	–3.553	0.556	0.392	0.812

Codon	α	β	β–α	Prob[α>β]	Prob[α<β]	BayesFactor[α<β]
43	5.518	0.744	–4.773	0.590	0.359	0.706
44	2.263	0.810	–1.452	0.528	0.416	0.897
45	0.884	0.666	–0.218	0.494	0.445	1.010
46	1.727	1.191	–0.536	0.495	0.448	1.023
47	2.263	0.810	–1.452	0.528	0.416	0.897
48	3.644	0.687	–2.957	0.566	0.380	0.773
49	4.646	0.942	–3.705	0.564	0.385	0.788
50	3.577	0.771	–2.806	0.762	0.194	0.302
51	1.727	1.191	–0.536	0.495	0.448	1.023
52	34.992	0.801	–34.191	0.995	0.002	0.003
53	3.643	0.812	–2.830	0.554	0.393	0.814
54	2.262	0.922	–1.340	0.520	0.424	0.926
55	2.303	0.744	–1.559	0.535	0.409	0.870
56	3.642	0.855	–2.787	0.551	0.396	0.824
57	2.263	0.773	–1.490	0.531	0.413	0.885
58	2.303	0.773	–1.530	0.533	0.411	0.880
59	2.303	0.773	–1.530	0.533	0.411	0.880
60	2.263	0.810	–1.452	0.528	0.416	0.897
61	2.263	0.810	–1.452	0.528	0.416	0.897
62	2.262	0.922	–1.340	0.520	0.424	0.926
63	5.518	0.724	–4.794	0.592	0.357	0.700
64	2.263	0.773	–1.490	0.531	0.413	0.885
65	4.647	0.895	–3.752	0.566	0.382	0.779
66	36.488	0.818	–35.670	0.996	0.002	0.002
67	2.262	3.825	1.563	0.262	0.688	2.771
68	4.648	0.786	–3.862	0.574	0.375	0.755
69	1.141	0.902	–0.239	0.487	0.455	1.049
70	4.646	0.942	–3.705	0.564	0.385	0.788
71	4.646	0.942	–3.705	0.564	0.385	0.788
72	20.286	0.739	–19.547	0.926	0.058	0.077
73	3.641	0.996	–2.645	0.543	0.404	0.853
74	3.643	0.826	–2.817	0.553	0.393	0.816
75	20.287	0.803	–19.485	0.923	0.060	0.080
76	3.645	0.659	–2.986	0.570	0.377	0.760
77	4.647	0.895	–3.752	0.566	0.382	0.779
78	0.771	0.811	0.041	0.468	0.471	1.121
79	1.141	0.902	–0.239	0.487	0.455	1.049
80	2.263	0.773	–1.490	0.531	0.413	0.885
81	2.263	0.773	–1.490	0.531	0.413	0.885
82	3.644	0.687	–2.957	0.566	0.380	0.773
83	2.262	0.922	–1.340	0.520	0.424	0.926
84	2.246	3.305	1.060	0.275	0.674	2.600
85	2.263	0.810	–1.452	0.528	0.416	0.897
86	3.641	0.996	–2.645	0.543	0.404	0.853
87	2.263	0.786	–1.477	0.530	0.414	0.889

Codon	α	β	β–α	Prob[α>β]	Prob[α<β]	BayesFactor[α<β]
88	3.643	0.812	–2.830	0.554	0.393	0.814
89	4.645	1.092	–3.553	0.556	0.392	0.812
90	1.727	0.896	–0.831	0.509	0.434	0.964
91	0.771	0.727	–0.043	0.476	0.463	1.083
92	2.263	0.786	–1.477	0.530	0.414	0.889
93	1.727	1.191	–0.536	0.495	0.448	1.023
94	2.303	0.744	–1.559	0.535	0.409	0.870
95	4.647	0.895	–3.752	0.566	0.382	0.779
96	3.643	0.757	–2.886	0.559	0.388	0.797
97	1.141	0.902	–0.239	0.487	0.455	1.049
98	4.648	0.786	–3.862	0.574	0.375	0.755
99	3.641	0.996	–2.645	0.543	0.404	0.853
100	2.263	0.810	–1.452	0.528	0.416	0.897
101	3.643	0.757	–2.886	0.559	0.388	0.797
102	3.641	0.996	–2.645	0.543	0.404	0.853
103	3.643	0.825	–2.818	0.553	0.393	0.816

Tables S14 Types of single nucleotide polymorphism (SNPs) and their locations in the partial fragment of the exon 1 of *Myf5* gene of catfish catfish populations in Thailand compare with the reference sequence (Accession number XM053509129)

No	Haplotype-specific	Variable nucleotide position and nucleotide substitution	Mutation type	Amino acid change (substitution)
1	*Clarias_Myf5*TH1–6,9,10,14,15,17*	44A→G	Silent	Ser to Ser (TCA to TCG)
2	*Clarias_Myf5*TH12,16*	56C→T	Silent	Ser to Ser (TCC to TCT)
3	*Clarias_Myf5*TH2,4–6,15*	60C→T	Silent	Leu to Leu (CTG to TTG)
4	*Clarias_Myf5*TH9,10*	72T→C	Missense	Ser to Pro (TCG to CCG)
5	*Clarias_Myf5*TH6*	77G→T	Silent	Ser to Ser (TCG to TCT)
6	*Clarias_Myf5*TH1–6,9,10,14,15,17*	92G→T	Silent	Ser to Ser (TCG to TCT)
7	*Clarias_Myf5*TH3*	96G→A	Missense	Glu to Lys (GAG to AAG)
8	*Clarias_Myf5*TH4*	111C→T	Missense	Arg to Trp (CGG to TGG)
9	*Clarias_Myf5*TH8,11,16*	137C→T	Silent	Ala to Ala (GCC to GCT)
10	*Clarias_Myf5*TH9*	176T→C	Silent	Arg to Arg (CGT to CGC)
11	*Clarias_Myf5*TH1–6,9,10,13,17*	172G→C	Silent	Ala to Ala (GCG to GCC)
12	*Clarias_Myf5*TH1–6,11,13,17*	224A→G	Silent	Arg to Arg (AGA to AGG)
13	*Clarias_Myf5*TH17*	226G→A	Missense	Arg to His (CGC to CAC)
14	*Clarias_Myf5*TH1–6,9–11,13,17*	242C→G	Silent	Val to Val (GTC to GTG)
15	*Clarias_Myf5*TH1–6,9–11,13,17*	251C→G	Silent	Ala to Ala (GCC to GCG)
16	*Clarias_Myf5*TH5*	227C→T	Missense	Ser to Trp (TCG to TGG)
